# Interactive Toxicogenomics: Gene set discovery, clustering and analysis in Toxygates

**DOI:** 10.1038/s41598-017-01500-1

**Published:** 2017-05-03

**Authors:** Johan Nyström-Persson, Yayoi Natsume-Kitatani, Yoshinobu Igarashi, Daisuke Satoh, Kenji Mizuguchi

**Affiliations:** 1 Level Five Co., Ltd., GYB Akihabara 3F, 2-25, Kanda-Sudacho, Chiyoda-ku, Tokyo, 101-0041 Japan; 2 Bioinformatics Project, National Institutes of Biomedical Innovation, Health and Nutrition (NIBIOHN), 7-6-8, Asagi, Saito, Ibaraki-shi, Osaka 567-0085 Japan; 3 Toxicogenomics-informatics Project, National Institutes of Biomedical Innovation, Health and Nutrition (NIBIOHN), 7-6-8, Asagi, Saito, Ibaraki-shi, Osaka 567-0085 Japan

## Abstract

Toxygates was originally released as a user-friendly interface to enhance the accessibility of the large-scale toxicogenomics database, Open TG-GATEs, generated by the Japanese Toxicogenomics Project. Since the original release, significant new functionality has been added to enable users to perform sophisticated computational analysis with only modest bioinformatics skills. The new features include an orthologous mode for data comparison among different species, interactive clustering and heatmap visualisation, enrichment analysis of gene sets, and user data uploading. In a case study, we use these new functions to study the hepatotoxicity of peroxisome proliferator-activated receptor alpha (PPARα) agonist WY-14643. Our findings suggest that WY-14643 caused hypertrophy in the bile duct by intracellular Ca^2+^ dysregulation, which resulted in the induction of genes in a non-canonical WNT/Ca^2+^ signalling pathway. With this new release of Toxygates, we provide a suite of tools that allow anyone to carry out in-depth analysis of toxicogenomics in Open TG-GATEs, and of any other dataset that is uploaded.

## Introduction

Biomedical research produces ever more data, which are often made publicly available at an early stage of investigation. The increasing popularity of open-access journals is now being matched by the availability of open data. In fact, some publication venues, for example GigaScience^1^, insist on publication of the data itself before any associated results may be published. This trend helps the research process, as important discoveries about a particular dataset can often be made by people who did not originally produce the data.

One example of this trend is the well-studied database Open TG-GATEs^2^, a large transcriptome (as well as associated phenotypes and biological data) database produced by Japanese Toxicogenomics Project (TGP) during 2002–2006 for TGP1^3^ and 2007–2011 for TGP2. The data were collected by a collaboration of the National Institute of Biomedical Innovation (NIBIO), the National Institute of Health Sciences and 15 pharmaceutical companies. This project aimed to create an infrastructure to evaluate the safety/toxicity of compounds, including drug candidates, by using these collected data, an approach referred to as toxicogenomics. It is expected that the public databases in toxicogenomics will support assessment of the safety of drug candidates in the early stage of the drug discovery process. This dataset has now been independently investigated by several different groups^4, 5^ as well as internally at NIBIOHN (formerly NIBIO), as one of the largest databases in toxicogenomics. It consists of approximately 24,000 microarray samples of about 200 different compounds, studied in rat tissues *in vivo* and rat- or human-derived primary cultured hepatocytes *in vitro*. Both single and repeat dose samples are available. Experimental conditions are well-defined and documented, and in theory, this dataset is well suited to the investigation of mechanisms of action in drugs and toxins that have not yet been explained.

However, data availability by itself is not always sufficient. In practice, even when data have been produced and published, considerable effort may be needed on behalf of interested third parties who wish to study them. When Open TG-GATEs had originally been released, it was necessary to perform substantial pre-processing before any investigation could commence. To lower the barrier to entry for new investigators, we developed Toxygates^6^ (http://toxygates.nibiohn.go.jp), the first version of which was made publicly available in 2013. At that time, the main achievement was to allow anybody who was interested to select samples quickly (e.g., compounds, exposure times) and look at expression data, visualise time or dose series, and show their annotations such as Gene Ontology (GO) terms^7^ or KEGG pathways^8^. Even with this basic feature set, Toxygates has seen regular use by researchers from a wide variety of countries and institutions since its release. During the year 2016, a total of 194 unique external users from 16 countries accessed Toxygates 17165 times to look at expression data. At the same time, this original version did not allow for sophisticated analysis and for further comprehensive analysis, such as clustering and enrichment, it was necessary to download the data and use external software. Analytical functions are important in practice to experimental biologists and thus, for user convenience, it is ideal to analyse data without downloading.

In spite of the growing demand for analytical applications to lower this barrier to large-scale databases such as Open TG-GATEs, the number of such applications is still limited. To the best of our knowledge, there are only two public web applications that utilise Open TG-GATEs even today; *LTMap* and *ToxDBScan*. LTMap (http://tcm.zju.edu.cn/ltmap/) is a web tool to compare input gene lists with reference gene lists, and it utilises Open TG-GATEs for reference gene lists to output a ranked list of drugs generated by rank-based pattern-matching algorithm^9^. ToxDBScan is also a web-based application for similarity search, utilising both Open TG-GATEs and DrugMatrix as reference data^10^. This tool calculates the similarity scores based on the extended connectivity fingerprints (ECFP) to compare input gene lists and references, and performs pathway enrichment analysis of input gene lists and visualises their result. In addition, *NFFinder* (http://nffinder.cnb.csic.es) is a web-based application with a similar concept that aims for drug repositioning^11^. NFFinder calculates similarity values using a weighted Kolmogorov-Smirnov-like statistic to compare an input gene list with their references, a database of gene signatures that were tagged with drugs and diseases by utilizing GEO^12^, Connectivity Map^13^ and DrugMatrix^14^, and outputs ranked lists of drugs or diseases whose gene signatures are positively/negatively correlated with the input gene lists. Although these applications have broadened the utility of toxicogenomics databases, their main concepts are common and the analytical methods offered are limited.

To advance beyond this status quo, we release the new Toxygates. With the new version described here, we provide a more sophisticated analysis environment, allowing a variety of analysis scenarios to be performed entirely within Toxygates. In what follows, we first give a brief general overview of Toxygates. We then describe the new and updated functions in this version, which include a set of interlocking functions for easy gene set discovery and management. Finally, in our case study, we bring these functions together to explore the mechanism of hepatotoxicity of WY-14643 to give insights into the complex molecular dynamics that this compound triggers.

## Results

New and improved functions in this version include (1) gene set management, (2) user data uploading, (3) clustering and visualisation, (4) gene set synchronisation and enrichment using TargetMine, and (5) an orthologous display mode, as well as (6) general interface improvements. For the details about how to use these functions, a user guide is available from “Help/feedback” button on the left corner of any pages in Toxygates, or from this URL (http://toxygates.nibiohn.go.jp/toxygates/toxygatesManual.pdf).

### Gene set management

**Figure 1:**
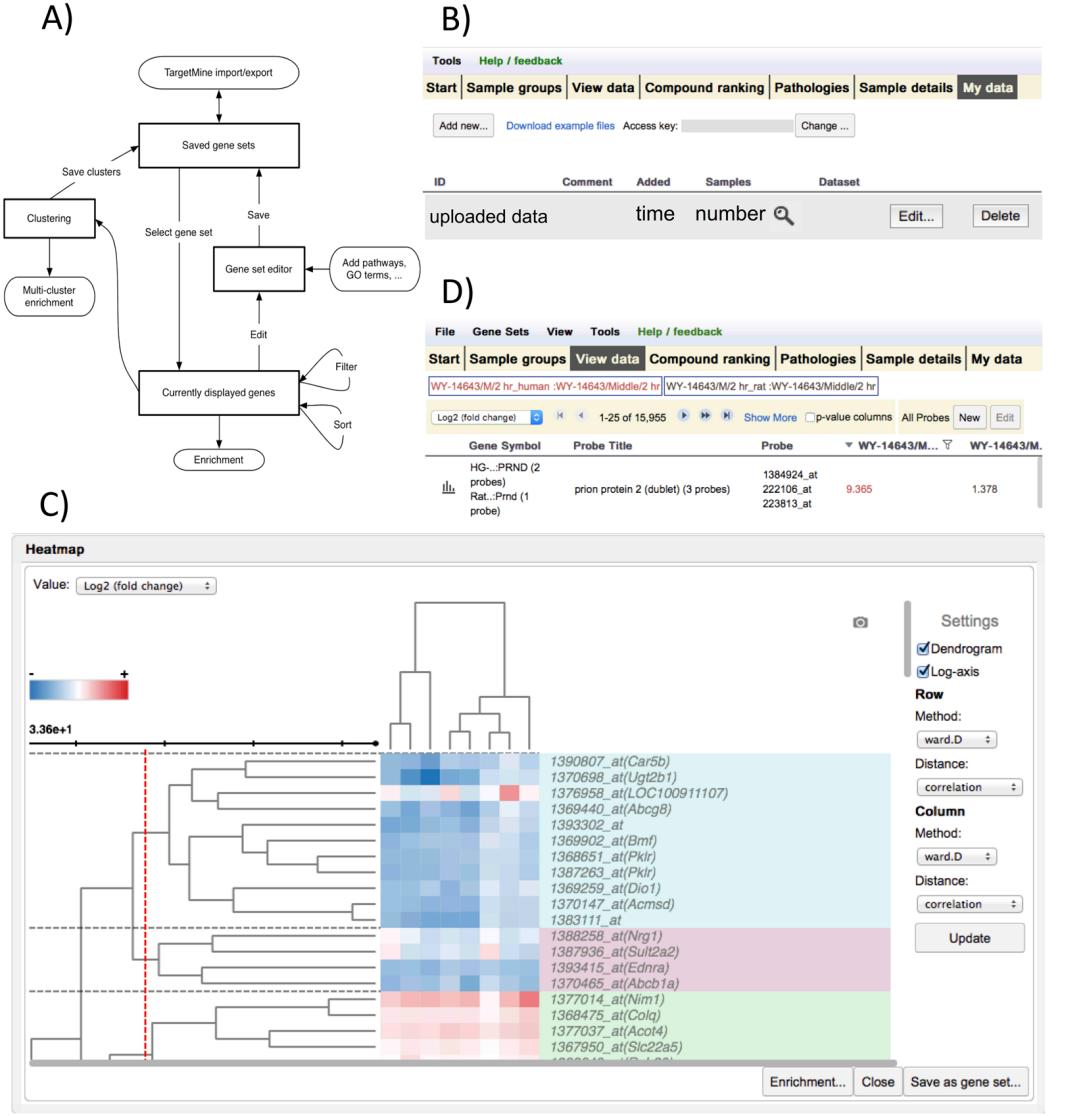
(**A**) Many functions in Toxygates now communicate with each other by modifying or transferring gene sets. Currently displayed genes can be filtered, sorted and tested for enrichment, and saved as a named set. They can also be clustered, and by setting a dendrogram cutoff, new gene sets can be generated, as well as enriched together. Gene sets are saved as local data in the web browser. They may be manually edited, for example by adding pathways and GO terms, and saved gene sets may be synchronised with TargetMine for further analysis and long term storage. (**B**) The *My Data* screen. Here, users can upload, adjust, and delete their own expression data as sets of samples (called *batches*). (**C**) Interactive display of heatmap with dendrogram. After selecting compounds and experimental conditions and filtering genes of interest, a clustering may be performed and displayed as a heatmap in a new pop-up window by selecting “Show heatmap” from “Tools” pull-down menu at the top of the main display. The parameters of the clustering may be changed by using the controls on the right side of the display. (**D**) When the currently defined sample groups contain more than one platform (i.e. more than one species), the orthologous mode is activated. This uses pre-computed groups of probes (based on amino acid sequence similarity) to display orthologous genes together as a single row. This allows for meaningful cross-species analysis, which is especially useful since Open TG-GATEs contains both rat and human data. The high-resolution image file is available at *Scientific Reports* online.

In basic Toxygates usage, first, sample groups are defined in terms of high-level parameters, for example, compounds, dose levels and exposure times. Then, expression data may be inspected on the level of individual probes, and studied together with relevant annotations. This operation is performed in a table that displays sample groups as columns and probes as rows. In this table, it is possible to focus on probes of interest. In the original Toxygates, probe sets were defined and managed on their own, separate screen. We have now eschewed this design in favour of managing them directly on the data screen, referring to them as gene sets. We believe that a major part of typical usage of Toxygates will consist of finding and refining an interesting gene set. Gene sets now have their own menu, from which gene sets may be created, selected or edited. For example, users might initially discover genes by sorting according to upregulation by some compound. They might then filter the genes by upregulation by other compounds, as well as by *p*-values that were calculated by comparison between control and sample-treated groups or between two sample groups of interest, and at each stage save their intermediate result as a new gene set. This function enables users to extract genes of interest according to their expression values (e.g. fold-change difference) or the results of statistical testing (e.g. Welch’s t-test and Mann-Whitney U-test) and pass them to subsequent functions as part of an analytical pipeline. Figure 1A shows how these gene set editing functions reinforce each other and how gene sets flow between the various tools.

### User data uploading

In this version of Toxygates, we allow users to upload their own data. By using this function, users can compare the expression profile of their data with that of a variety of drugs in Open TG-GATEs easily. It should be noteworthy that this function is provided for free, whereas other analytical tools such as Ingenuity® Pathway Analysis (http://www.qiagen.com/ingenuity) and MetaCore® (http://protal.genego.com) that have a similar function provide it as a fare-paid service. Uploaded data can be analysed alongside the existing data, using all of the available analysis functions. User data are managed as batches. One batch is a set of samples, and these data may be uploaded, adjusted and deleted from the new *My Data* screen (Fig. 1B). Currently, only uploading of Affymetrix data - from the prescribed Mouse (GeneChip® Mouse Genome 430 2.0 Array), Rat (Rat Genome 230 2.0) or Human (Human Genome U133 Plus 2.0) platforms - is supported. At least two files must be supplied: a metadata file that describes the samples and their attributes, and a normalised data file. Users are allowed to define parameters such as new tissues, compounds and time points, though some parameters are restricted. We provide downloadable example files for reference; these files describe the acceptable parameters for user data in detail.

By default, user data are private and not shared with other users, protected by a unique user key. While this provides reasonable privacy, Toxygates is not intended to be secure against a sophisticated attack, and users may only upload sensitive data at their own risk. As long as this is acceptable, we believe that our solution provides a good trade-off between convenience and security, as users can easily begin uploading data.

### Clustering and visualization

Once a gene set of interest has been defined, it may be clustered and visualised as a heatmap by using the *show heat map* command on the tools menu. The visualisation functionality comes from InChLib^15^. The clustering itself is carried out using the HClust R function on the server side. For performance reasons, we currently limit clustering to sets of 1000 genes or less. In the clustering interface, various clustering methods and distance metrics, as well as other parameters, may be chosen (Fig. 1C). By clicking on any point in the vertical dendrogram (which displays the hierarchy of genes, whereas the horizontal one displays the hierarchy of sample groups), a cutoff may be set. Such a cutoff partitions the set of clustered genes into some number of subsets (clusters). Such a clustering result may be tested for enrichment analysis (by using TargetMine^16–18^ functionality, which we discuss below). This function is useful when users wish to group genes of interest into several clusters according to the expression patterns, by inspecting both the heatmap and the dendrogram. Furthermore, this heatmap screen allows users to perform a simplified version of enrichment analysis for temporarily obtained clusters by setting a cutoff for the dendrogram. When a certain feature is enriched in the clusters, the single most highly enriched feature (such as pathway or GO term) is displayed for each cluster, along with its associated *p*-value. This result is displayed as a pop-up screen, so that users can refer to this result during the process of tuning a cutoff. As a rough indicator, this function may sometimes help users choose a reasonable cutoff value, and TargetMine supports more detailed enrichment analysis as described below.

### Gene set synchronisation and enrichment using TargetMine

TargetMine is an integrated data warehouse mainly intended for drug target discovery. It is based on the InterMine^19^ framework and has been under development at NIBIOHN for some time. When a single gene set is tested for enrichment in Toxygates, all significantly enriched features and their associated *p*-values will be displayed within a few seconds, by creating an anonymous, temporary gene list in TargetMine. Thus, for basic enrichment testing, no user login details are necessary. Enrichment analysis is available for a variety of biological features such as KEGG pathways^8^, GO terms^7^, GOSlim terms and Integrated Pathway Clusters (IPCs)^17^, and all these results are displayed in one screen without specifying which enrichment analysis to perform in advance. The methodology of these enrichment analyses is discussed in Chen YA *et al*.^16^. This application helps users to find which biological feature is shared among genes in the input list without making any *a priori* assumptions. Since the strength of TargetMine is easy retrieval of a variety of biological knowledge, platform connection with TargetMine adds significant value to Toxygates. In this version of Toxygates, we have used the TargetMine application programming interface (API) to integrate the functionality of the latter into the former. Going beyond the basic enrichment described above, this functionality integration allows users to begin their studies in either of these two tools and proceed to the other by synchronising gene lists. The *import gene lists* and *export gene lists* commands on the Tools/TargetMine data menu perform these functions, once the login details of a TargetMine account (which are freely available to any interested users) have been supplied. Thus, users can perform several types of enrichment analysis all at once for each of the clusters that were obtained by clustering analysis described above by exporting the gene lists of these clusters. TargetMine is under active development and appropriate maintenance to ensure the accessibility of up-to-date annotations. Thus, it is expected that this functionality integration generates further synergistic effects and additional analytical functions may become available in this way in the future.

### Orthologous display mode

Orthologous mode enables users to compare the expression patterns among different species, which can be helpful to extrapolate the knowledge that is obtained in experimental animals to human or vice versa. This mode is automatically activated if the user activates groups from multiple platforms or species simultaneously (Fig. 1D). In the original version of Toxygates, every row of the main data table would display strictly one Affymetrix GeneChip probe. In orthologous mode, a set of orthologous probes from the various selected platforms (Affymetrix GeneChips) for different species is computed, by using orthology information. We currently use SSEARCH^20^ sequence similarity search as a basis for orthologous probe sets, as defined in TargetMine^16^. This operation bases the similarity score on the amino acid sequence of each probe’s corresponding protein, if any. For such an orthologous probe set, the result is then displayed as the median of all expression values in the set. (Since the basic values displayed in the “normal” mode are averages of the probe values for all samples in each group, such a number ends up being a median of averages).

### General interface improvements

In the original version of Toxygates, each column could have a cutoff threshold to filter the data table. However, only one threshold type was available, depending on the column: a lower limit on absolute value for data columns, and an upper limit for *p*-value columns. There was also no visual indication as to whether a column had an active filter or not. We have now added such an indicator - the filter icon above each column becomes blue if a filter is active - as well as different user-selectable filter types for all columns. Filtering can now be an upper bound, an upper bound on absolute value, a lower bound, or a lower bound on absolute value.

We have refined the Toxygates user interface since the first version, with the intention of making it more versatile, expandable and easier to understand. In particular, we removed the six “data sets” icons that used to be the starting point for Toxygates data analysis (e.g., Rat/*In Vivo*/Liver/Single Dose, Rat/*In Vitro*/Liver) as we expect the data content of Toxygates to become too diverse in the near future to be represented by six categories only. A set of drop-down boxes in the top left corner of the group definition screen now perform this function in a more general way, allowing the user to specify a species, test type (“*in vivo*” or “*in vitro*”), tissue and dose class (“Repeat” or “Single”). Finally, the compound ranking function was moved from the group definition screen to its own separate top-level screen, to make this function easier to find.

A number of improvements allow Toxygates to load data much faster than before. In the best case, Toxygates is now able to load 108 samples and 31042 probes in about 8 seconds. These values can then be immediately used for further processing such as filtering and sorting.

Charts (time series or dose series) can now be easily downloaded. Data downloading (as CSV files) has also been improved. We now provide two separate download commands: one for grouped samples and the other for individual samples. This function should be valuable to users who wish to carry out further statistical analyses on their own, for example by computing their own *p*-values.

### Case study

We now show how to use the various functions of the updated Toxygates together in practice to explore the toxicological properties of a specific compound. WY-14643 (pirinixic acid) was originally developed as an anti-hypercholesterolemic agent^21^, but is nowadays solely used for experimental purposes because of its undesired side effects, such as hepatocarcinogenicity in rodents. It has now become known that WY-14643 works as a selective agonist of Peroxisome Proliferator-Activated Receptor alpha (PPARα). The detailed analytical flow of this case study is shown in our Supplementary Methods.

### Observed pathologies as a starting point

Open TG-GATEs data showed that WY-14643 exhibited liver toxicity under certain experimental conditions. Its pathology data (available on the Pathologies screen in Toxygates) showed that single administration of WY-14643 for 6 hrs or more, as well as repeated administration, caused liver pathologies such as “Degeneration, granular, eosinophilic” in hepatocytes. Repeated administration also caused “Necrosis”, “Single cell necrosis”, “Hypertrophy”, “Increased mitosis” and “Cell infiltration” (Supplementary Table S1). Among these, “Increased mitosis” was observed only at early stages (mainly at the 4 day timepoint) and “Hypertrophy” was observed at late stages (8 days or later).

### Cluster analysis

In order to explore the difference between 4 day treatment and 8 day treatment at transcriptional level, which may explain the difference between these time points in pathological observation, we investigated the gene expression profile at each time point for comparison. Based on these pathological findings, we chose the middle dose data (Rat/*in vivo*/liver/repeat), which showed the clearest time-dependency in liver damage development and defined one sample group for each time point (at 4, 8, 15 and 29 days). We used the filtering option to extract the genes that had log-2 (fold change) values greater than or equal to 1.5 against the control group for each time point to see the difference in gene expression profile among these experimental conditions, and then grouped these genes into clusters according to their expression patterns by using the heatmap function (dendrogram cutoff = 4, Supplementary Figs S1–4). The gene list of each cluster was exported to TargetMine for KEGG pathway enrichment analysis. The result showed that genes that were part of “PPAR signalling pathway [rno03320]” were enriched in upregulated clusters in all the middle-dose groups (Supplementary Table S2). Since the PPARα signalling pathway is responsible for the proliferation of organelles such as peroxisome, the observation above suggests that the overactivation of PPARα was the direct cause of the observed “Degeneration, granular, eosinophilic” in hepatocytes.

### Compound ranking to discover similar compounds

It was also found that genes mapped to “Steroid hormone biosynthesis [rno00140]” were downregulated at all time points (Supplementary Table S2). The compound ranking function in Toxygates found that fenofibrate and methapyrilene downregulate genes in a manner similar to that of WY-14643 (See *Methods* for details). Fenofibrate is a well-known PPARα agonist that belongs to fibrates, which have been associated with hepatotoxicity, and methapyrilene is an antihistamine and anticholic drug. It is noteworthy that methapyrilene causes hepatotoxicity via oxidative stress and mitochondrial dysfunction, which is similar to that caused by PPARα agonists^22^, and that this toxicity of methapyrilene was prevented by the administration of verapamil, a Ca^2+^ channel blocker^23^. Thus, intracellular Ca^2+^ appears to be involved in the downregulation of these genes.

### Welch’s t-test filtering

Significant differences were observed between the M4 (4 day) and M8 (8 day) time points. Initially, the “increased mitosis” pathology had been observed at M4, and bile duct hypertrophy had been observed at M8. Consistently with this observation, for WY-14643, genes part of “Cell Cycle [R-RNO-1640170]” were upregulated only in M4 and genes related to “Bile secretion [rno04976]” were downregulated in all data except for M4 (Supplementary Table S2). To investigate the change that appears to have occurred between M4 and M8, we extracted the differentially expressed genes (DEGs) between these two sample groups by adding a t-test column (threshold: *p*-value = 0.01), which resulted in extracting 618 probes. For these genes, we then compared the WY-14643 influenced gene expression profiles with those of amlodipine, a known Ca^2+^ channel blocker, to understand their action in the context of Ca^2+^ signalling.

### Uploading user data to aid the investigation

**Figure 2:**
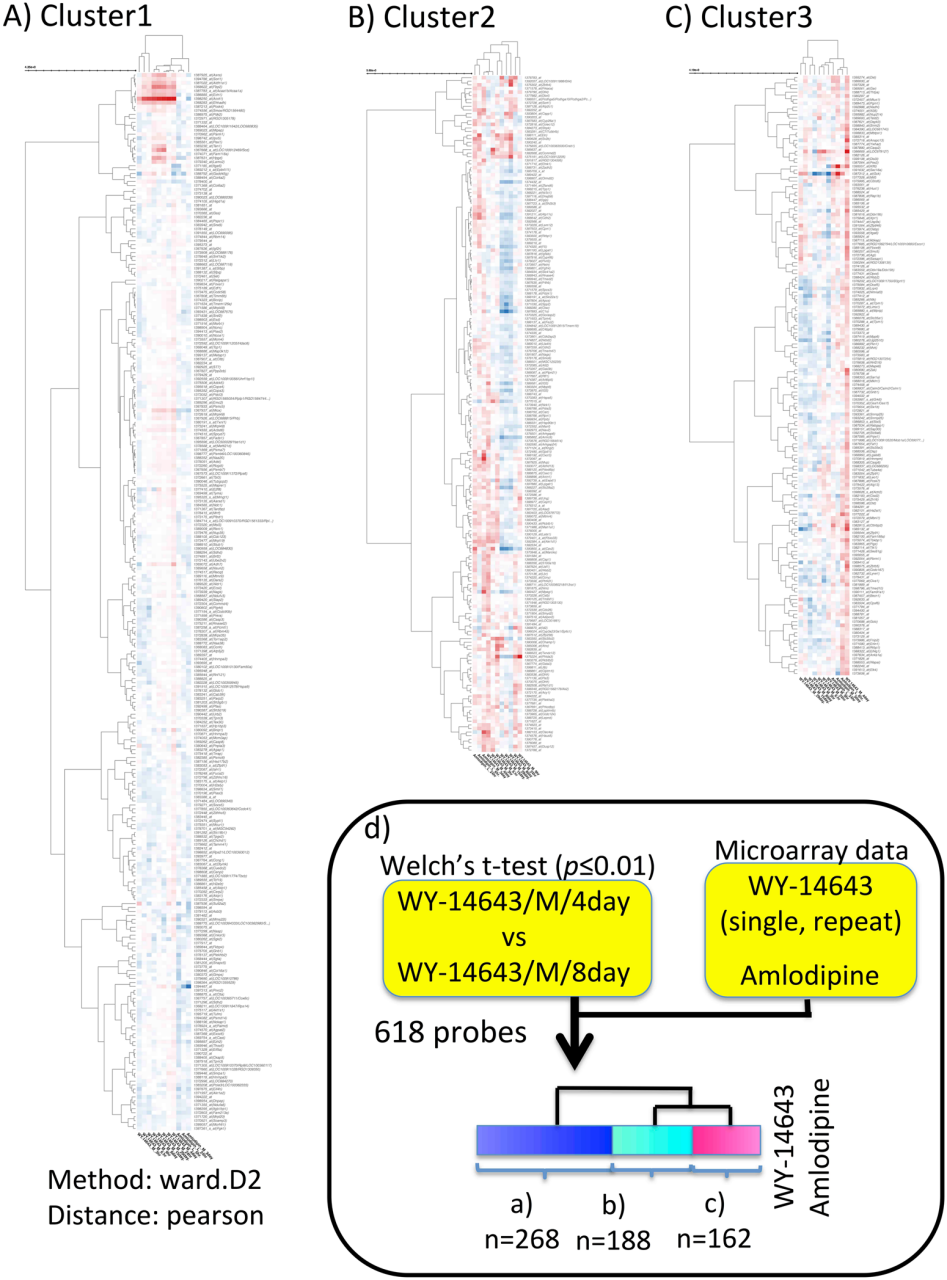
Heatmap of gene expression profiles obtained by treatment of WY-14643. The dendrogram was made without log-transformation of the axis, according to a hierarchical clustering result (method: ward.D2, distance: Pearson). (**A**) Gene expression profile of M4vs8_cluster1 (probe: n = 268). The x-axis represents experimental conditions (from left to right, (1–8) WY-14643 3 hr, 6 hr, 9 hr, 8 day, 15 day, 29 day, 24 hr and 4 day; (9–11) amlodipine 6 hr, 24 hr and 3 day. The y-axis represents probes in M4vs8_cluster1. (**B**) Gene expression profile of M4vs8_cluster2 (probe: n = 188). The x-axis represents experimental conditions (from left to right): (1–3) amlodipine 24 hr, 6 hr and 3 day; (4–11) WY-14643 4 day, 3 hr, 6 hr, 15 day, 29 day, 8 day, 24 hr and 9 hr. The y-axis represents probes in M4vs8_cluster2. (**C**) Gene expression profile of M4vs8_cluster3 (probe: n = 162). The x-axis represents experimental conditions (from left to right, 1–7) WY-14643 29 day, 15 day, 8 day, 3 hr, 6 hr, 24 hr and 9 hr; (8–10) amlodipine 24 hr, 3 day and 6 hr; 11) WY-14643 4 day). The y-axis represents probes in M4vs8_cluster3. (**D**) Analytical workflow for obtaining three M4vs8_clusters in (Fig. 2A–C). A gene set was selected by filtering DEGs between WY-14643/M dose/4 day and WY-14643/M dose/8 day by *Welch*’s t-test (cutoff: *p*-value = 0.01). As columns, Open TG-GATEs samples of WY-14643 (single dose (30 mg/kg bw): 3 hr, 6 hr, 9 hr, 24 hr, repeat dose (30 mg/kg bw): 4 day, 8 day, 15 day, 29 day) and external samples of amlodipine (uploaded to Toxygates by “user data upload function”, L dose (0.2 mg/kg), 6 hr or 24 hr, and M dose (19 mg/kg), 3 day) were used. The clusters were obtained by hierarchical clustering (dendrogram cutoff: 6). The high-resolution image file is available at *Scientific Reports* online.

Amlodipine data are not included in Open TG-GATEs, but the *My Data* function in Toxygates may be used to upload and study such data as a positive control of Ca^2+^ dynamics dysregulation (see *Methods* for details). To compare the gene expression profiles of WY-14643 with amlodipine, we made a heatmap by selecting WY-14643 data (Rat/*in vivo*/liver/single and repeat dose) and amlodipine data for DEGs between M4 and M8. Three clusters were generated by setting the cutoff threshold to 6 (Supplementary Tables S5–7). The genes in M4vs8_cluster1 (n = 268) showed a tendency to be upregulated by WY-14643 (M24hr, M4, 8,15, 29 day) (Fig. 2A). Pathways related to beta-catenin independent WNT signalling were enriched. In M4vs8_cluster2 (n = 188), genes showed a tendency to be downregulated by repeated administration of WY-14643 (M8, 15, 29 day) (Fig. 2B). Also, integrated pathway clusters (IPCs) such as “Protein processing in endoplasmic reticulum” were enriched, and biomarkers of unfolded protein response (UPR) such as Hspa5/Grp78^24^ were included in this cluster. The genes in M4vs8_cluster3 (n = 162) were upregulated only in M4 and amlodipine data, and pathways related to Ca^2+^ signalling were enriched (Fig. 2C). These results are summarized in Supplementary Table S4, and the brief workflow how these three clusters were generated is depicted in Fig. 2D.

### Ca^2+^ dysregulation may influence the balance between PPARα and Wnt/Ca^2+^ signalling pathways

**Figure 3:**
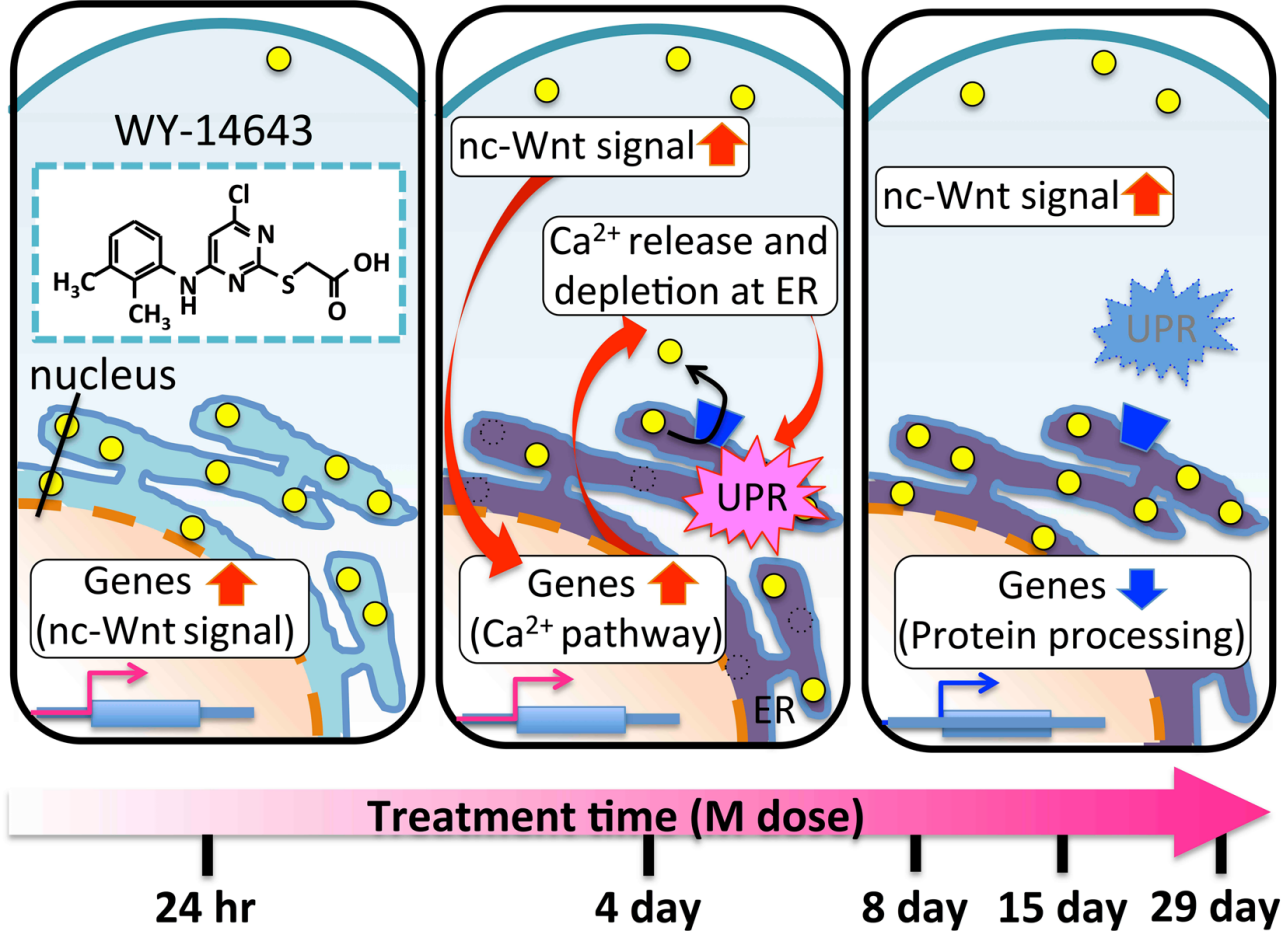
Hypothetical model of the hepatotoxicity caused by WY-14643. Genes in M4vs8_cluster1 were upregulated predominantly in M24hr and M4. This results in elevated sensitivity of beta-catenin independent Wnt signaling and induction of Ca^2+^ pathway-related genes, followed by decreasing intracellular Ca^2+^ and Ca^2+^ release from ER in M4. Upregulation of genes in M4vs8_cluster3, which were also upregulated by amlodipine, implies the Ca^2+^ dynamics dysregulation in M4. Also, temporary upregulation of genes in M4vs8_cluster2 in M4 implies UPR response, which is a survival signalling from ER stress that can be caused by Ca^2+^ depletion at ER^31^. By long-term treatment of WY-14643 (M8, M15, M29), upregulation of genes in M4vs8_cluster1 (enriched pathways: ﻿“beta-catenin independent Wnt signalling﻿”) and downregulation of genes in M4vs8_cluster 2 (enriched IPCs: “Protein processing in ER”) were observed. Since beta-catenin dependent Wnt signaling directly regulates cell cycle^32^ and beta-catenin independent Wnt signalling (non-canonical Wnt signalling, which is represented as ﻿“nc-Wnt signal﻿” in Fig. 3) antagonises beta-catenin dependent Wnt signalling^33, 34^, it is implied that WY-14643 disrupts proper cell cycle regulation by inducing genes in beta-catenin independent Wnt signaling, while causing organelle proliferation by persistently activating PPARα signalling. The high-resolution image file is available at *Scientific Reports* online.

Based on the Toxygates analysis, we have built a hypothetical model of the hepatotoxicity caused by WY-14643 (Fig. 3). In a normally functioning cell, cell growth/cell cycle will be balanced. However, it appears that WY-14643 upsets and unbalances both of these mechanisms through interference with Ca^2+^ signalling, allowing peroxisome proliferation to cause hypertrophy, and thus hepatotoxicity. Since Wnt/Ca^2+^ signalling has been linked to the prognosis of breast and colon cancer^25^, WY-14643 may also be involved in hepatocarcinogenicity through similar mechanisms. Although many details remain to be elucidated, our findings suggest that the condition of treatment with PPARα agonist drugs should be chosen carefully, so as to not influence the balance between the PPARα and Wnt/Ca^2+^ signalling pathways.

In this way, the new functions of Toxygates were used to identify gene sets of interest visually, characterise them, as well as further analyse them in TargetMine, to support an interactive toxicological investigation process. A hypothesis can be formulated and gradually refined, validated or invalidated, as multiple analysis functions reinforce each other.

## Discussion

Although several consortiums and projects such as eTOX^26^ have been developed to promote toxicological data sharing with the aim of effective drug development, safety evaluation and toxicity prediction, toxicogenomics datasets that are open to the general public are still of limited availability. There has been an increasing demand for applications to enhance the availability of such shared data.

Web-based resources in bioinformatics can be broadly classified into *datasets*, *analytical tools*, or a combination of the two. By datasets, we mean an archive of data that has been directly recorded from an assay (such as Affymetrix GeneChip) and undergone only minimal processing, such as normalisation. Open TG-GATEs falls in this category, as does (for the most part) DrugMatrix. Another example is the US FDA-led project called “Sequencing Quality Control (SEQC)”, which generated RNA-seq data for 27 chemicals with three rat livers per chemical (GSE55347)^27^. Janssen (formerly Johnson & Johnson) have released their Toxicogenomics database as well, which stores Codelink and Affymetrix microarray data of rat liver for 124 liver-active compounds, generated between 2004–2008^28^. The data are available at Strand Life Sciences web page (http://pubdata.strandls.com) and NIH CEBS database^29^ (http://www.niehs.nih.gov/research/resources/databases/cebs/). Although the authors also offer an R package for data and connectivity map-based analysis, this resource is an example of a pure dataset, as no analytical interface is provided and thus the barrier to entry may be relatively high for new researchers.

By analytical tool we mean a derived resource, such as a graphical user interface, an algorithm, or a precomputed database with the outputs of an algorithm. In this way, analytical tools add meaningful perspectives to some underlying data. Toxygates falls in this category as it adds a user interface and interactive analysis functions and algorithms on top of the Open TG-GATEs dataset. LTMap, ToxDBScan and NFFinder are also in this category. These three all accept a pair of gene lists as input, one list of up-regulated genes and one of down-regulated genes by a compound of interest. These applications sort reference drugs by computing the similarity between the query and references. Toxygates can search for compounds that up- or downregulate given genes by using the compound ranking function. However, in general, Toxygates is now an open-ended analysis environment with a broad range of functions, while the tools mentioned above have much more specific purposes.

Although ToxDBScan and NFFinder utilise a wider range of data than Toxygates, we believe that our policy to utilise only Open TG-GATEs as a reference database is favourable to obtain biologically informative results at this point in time. Although we successfully obtained informative results in our case study by importing external data into Toxygates, in general, combining different datasets can give rise to problems of data normalisation and standardisation. However, in the long term, we may also insert other well-known datasets, such as DrugMatrix, into Toxygates, to facilitate more comparisons, once we have decided on a suitable solution to this problem.

It is easy to see how the principles of open data give rise to new research possibilities; if datasets are freely available and well documented, and if analytical tools have the ability to accept new data (and not just the dataset that they were constructed for), then analytical perspectives and datasets may be combined freely. We believe that in order to have the greatest value, analytical tools should have functions for user data upload in order to be applicable to the widest possible range of datasets.

We now provide a uniquely integrated environment where many tools can be combined easily and freely, as we demonstrated in our case study. To the best of our knowledge, our close integration with TargetMine and our orthologous display mode are features unique to Toxygates at this time of writing. Moreover, Toxygates is designed to ensure its versatility and extensibility. We have already launched another project to develop a new database with different concepts for human- and mouse-derived data, and the structure of Toxygates can be extended to such an independent database without technical difficulties. In the future, we aim to improve our analysis tools further in terms of their applicability and ease of use. As shown here, Toxygates accelerates computational analysis of transcriptome data by researchers with limited or no bioinformatics skills of their own, and is expected to support a broad range of research activities including toxicogenomics, pharmacogenomics and chemical genomics.

## Methods

The basic methods remain as they were in the original version of Toxygates, with some minor changes and updates.

### Microarray data and pathological observations

All the data stored in Toxygates were obtained by the Toxicogenomics Project^3^. Toxygates stores the data processed as below, and their raw data are open to public by Open TG-GATEs^2^.

### Data normalization

Normalisation was carried out in R by using Bioconductor’s *affy* package^30^. To calculate normalised intensities, “absolute value” display (as opposed to log-2 fold change) has changed. Previously this calculation was done using mas5 normalisation, with the parameter normalize = T. However, we now use mas5 with normalize = F and divide by the median of the values. This operation is the same as the method we use for log-2 fold change values. The benefit is added clarity and the possibility for users to reconstruct the log-2 fold change values easily by themselves.

### Annotation data

As in the original version of Toxygates, our data architecture is based on a “hybrid” approach, where the main data tables, which have a static and predictable structure, are stored in a flat key-value store. Annotations of rows and columns (genes and samples) in this table are stored as RDF data, which may be on local or on remote servers. The local annotations, which include GO terms and KEGG pathways, had in fact not been updated for some time in the old version of Toxygates. However, we have now streamlined the annotation update process, and we now update such annotations frequently (currently on a weekly basis).

### Calculation of log-2 mean values

Since the original version of Toxygates, we have changed the calculation of log-2 mean values. Originally, the log-2 of means of expression values for each dose and time combination was computed and stored in the database. However, when users displayed data, the mean of several such logarithms would sometimes need to be computed, for example when users had combined different doses and times, or compounds in the same group. In this version, we simplify this operation by storing fold values prior to log-2 computation in the database. When users define and display a group, we compute the mean of all samples in that group, across all parameter combinations in it, and only then apply the log-2 transformation.

### Source code

Toxygates is now open source, released under the GNU GPL license (v 2.0 or later). A public repository is available at http://bitbucket.org/jtnystrom_nibio/toxygates. We intend to publish build and deployment instructions in the near future, and we hope that other researchers will want to make use of or contribute to the code base.

### Compound ranking

The genes that were downregulated by repeated administration of WY-14643 (defined as “WY-downregulated genes”, n = 73) were extracted by filtering the genes whose middle-dose expression value was half of or less than that of the corresponding control group at all the time points (4, 8, 15, and 29 days). The compound ranking function was used by “total downregulation” mode to Rat/*in vivo*/liver/repeat data, to investigate which compounds in Toxygates downregulate these genes in a manner similar to that of WY-14643.

### External dataset for case study

The microarray data of amlodipine and their control samples, which was obtained from the rat liver samples after the treatment of 0.2 mg/kg of amlodipine (6 hr or 24 hr, which we call L6hr or L24hr, respectively), or 19 mg/kg of amlodipine (3 days, which we call M3day), is part of DrugMatrix^14^. The raw data of these experiments were downloaded as CEL files (L6hr: GSM1392780, GSM1392866, GSM1392977, control for L6hr: GSM1393754, GSM1392778, GSM1392793, GSM1392872, GSM1392876, GSM1392968, GSM1393317, GSM1393325, GSM1393346, GSM1393349, GSM1393355, GSM1393369, GSM1393388, GSM1393400, GSM1394151, GSM1354152, GSM1354154, GSM1354155, GSM1354158, GSM1354164, L24hr: GSM1392548, GSM1353158, GSM1353503, control for L24hr: GSM1392449, GSM1392501, GSM1392934, GSM1392956, GSM1393503, GSM1393051, GSM1393082, GSM1393158, GSM1393186, GSM1393200, GSM1393423, GSM1393479, GSM1394027, GSM1394041, GSM1394043, GSM1394060, M3day: GSM1392528, GSM1392628, GSM1393514, control for M3day: GSM1392575, GSM1392992, GSM1393059, GSM1393085, GSM1393116, GSM1393243, GSM1393424, GSM1393477, GSM1393482, GSM1394040, GSM1394061, GSM1394374). After normalising the data in the same way as Open TG-GATEs (see above), we created input files for user data-upload function on Toxygates.

## Electronic supplementary material

Supplementary Figures and Tables

Supplementary Methods
