# Supplementary material for: Interactive Toxicogenomics: Gene set discovery, clustering and analysis in Toxygates
Source: Sci Rep. 2017 May 3;7:1390. doi: 10.1038/s41598-017-01500-1 (PMC5431224; doi:10.1038/s41598-017-01500-1)
Supplement: Supplementary file 1 — Supplementary Figures and Tables [file 41598_2017_1500_MOESM1_ESM.pdf]

Interactive Toxicogenomics: Gene Set Discovery, Clustering and Analysis in Toxygates

Nyström−Persson, J., Natsume-Kitatani, Y., Igarashi, Y., Satoh, D., and Mizuguchi, K.

Supplementary Figures and Tables

Supplementary Figure S1

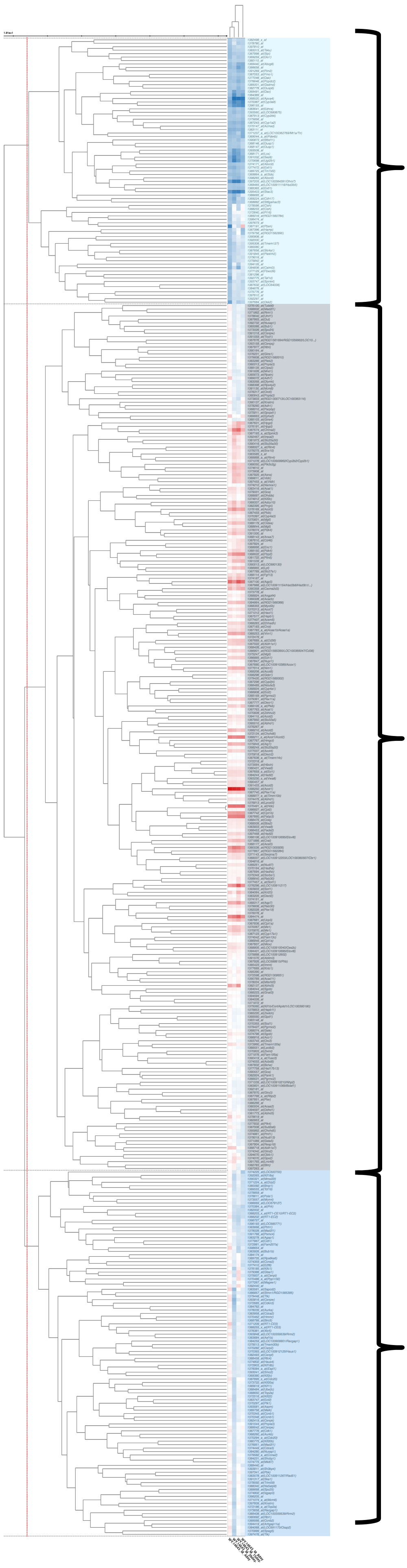

M dose/4 day (434 probes)

Steroid hormone biosynthesis
[rno00140]
77 probes

PPAR signaling pathway
[rno03320]
244 probes

Cell Cycle
[R007]
113 probes

Supplementary Figure S1
Heatmap of gene expression profiles obtained by treatment of WY-14643 in genes whose expression intensities were affected in M dose/4 day
The x-axis represents experimental conditions (from left to right, 1) WY-14643 4day, 2) WY-14643 29day, 3) WY-14643 15day, 4) WY-14643 8day. The y-axis represents probes whose log-2 (fold change) value against the control group was 1.5 or more by 4day treatment of WY-14643.

Supplementary Figure S2

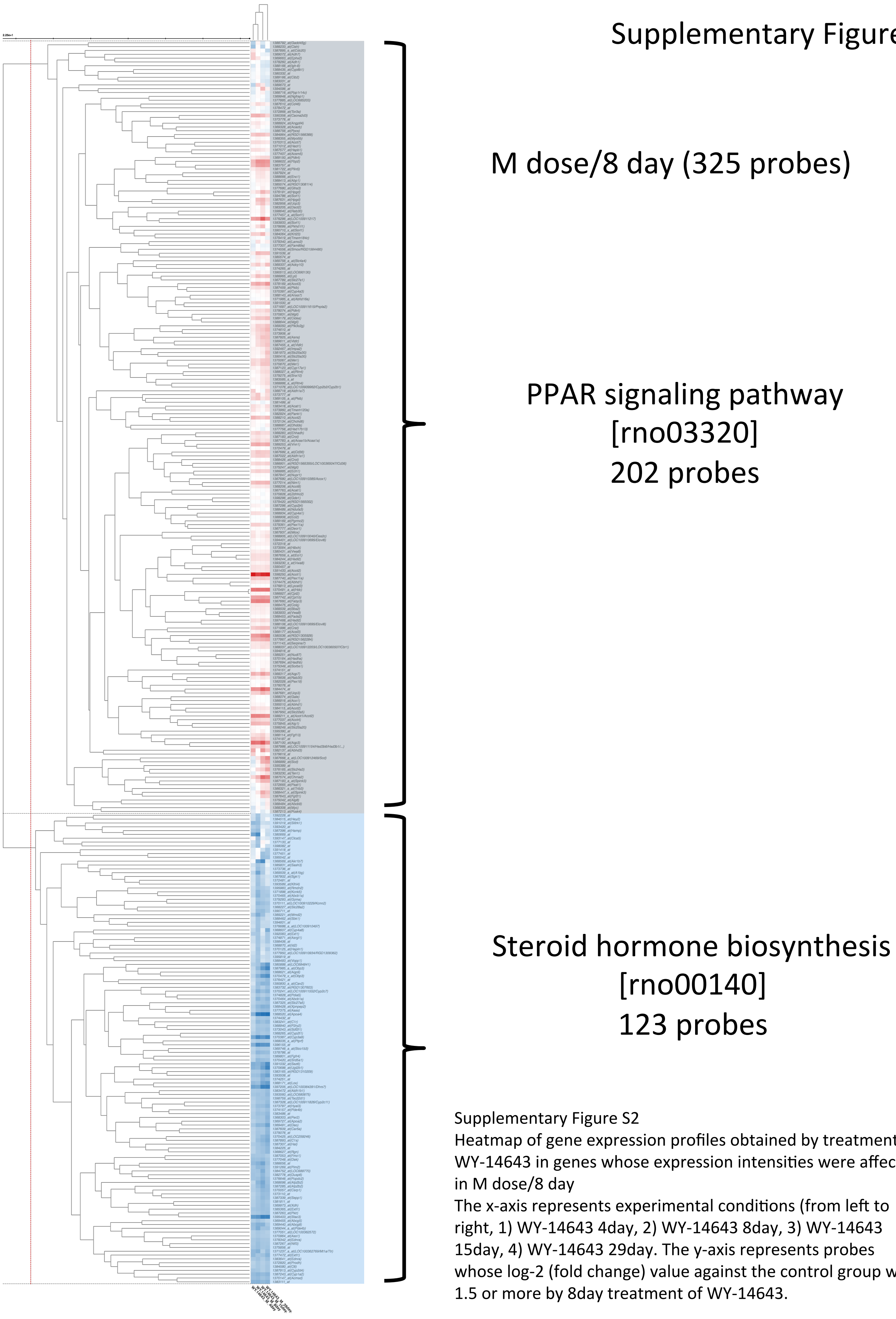

Supplementary Figure S2
Heatmap of gene expression profiles obtained by treatment of WY-14643 in genes whose expression intensities were affected in M dose/8 day
The x-axis represents experimental conditions (from left to right, 1) WY-14643 4day, 2) WY-14643 8day, 3) WY-14643 15day, 4) WY-14643 29day. The y-axis represents probes whose log-2 (fold change) value against the control group was 1.5 or more by 8day treatment of WY-14643.

Supplementary Figure S3

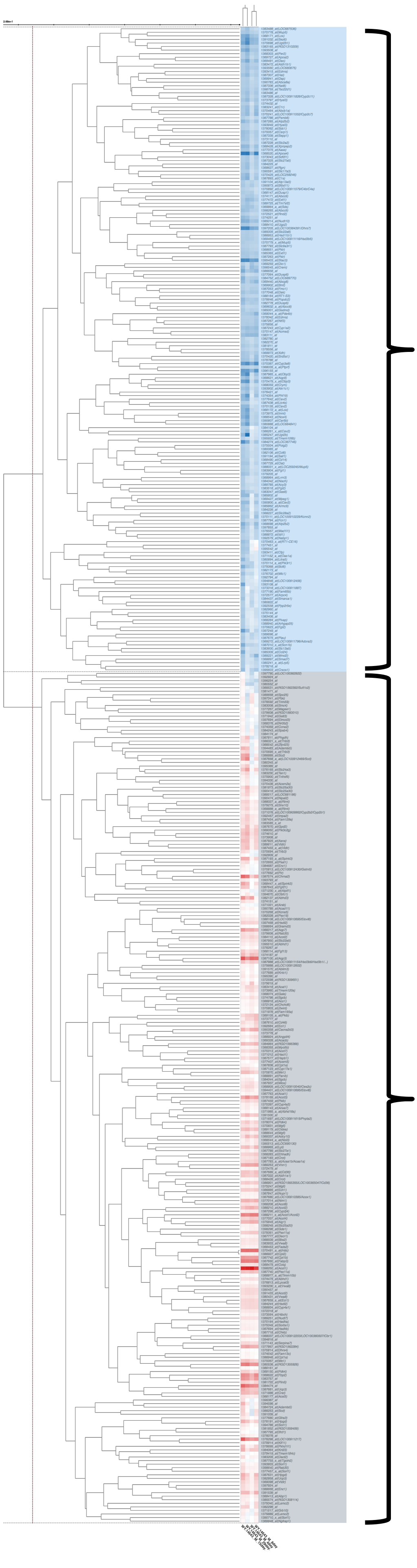

Supplementary Figure S3
Heatmap of gene expression profiles obtained by treatment of WY-14643 in genes whose expression intensities were affected in M dose/15 day
The x-axis represents experimental conditions (from left to right, 1) WY-14643 15day, 2) WY-14643 29day, 3) WY-14643 4day, 4) WY-14643 8day. The y-axis represents probes whose log-2 (fold change) value against the control group was 1.5 or more by 15day treatment of WY-14643.

Supplementary Figure S4

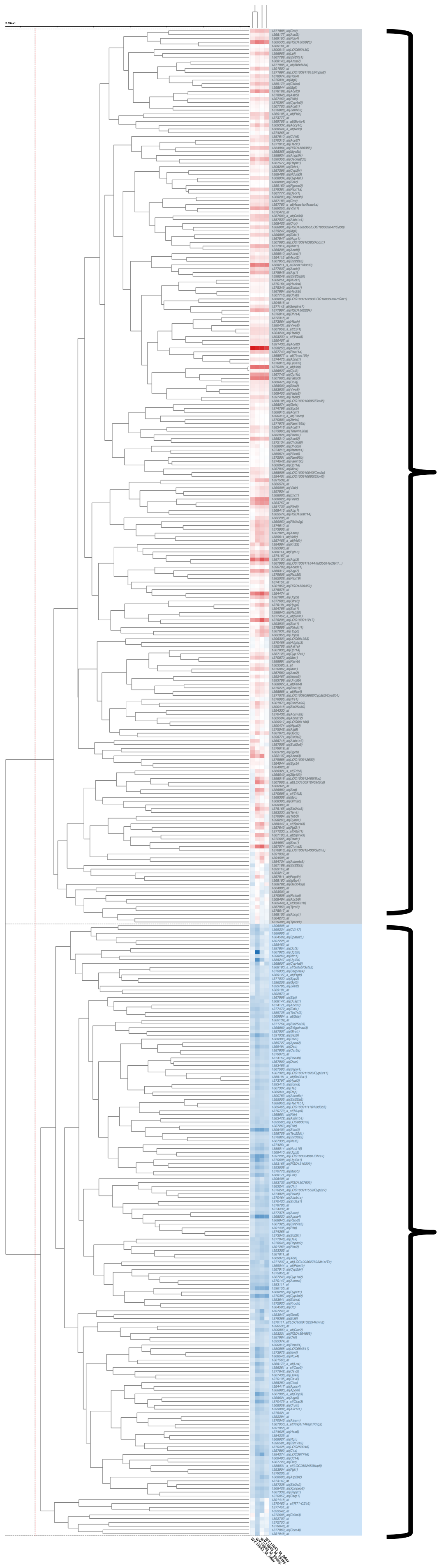

Supplementary Figure S4
Heatmap of gene expression profiles obtained by treatment of WY-14643 in genes whose expression intensities were affected in M dose/29 day
The x-axis represents experimental conditions (from left to right, 1) WY-14643 4day, 2) WY-14643 29day, 3) WY-14643 15day, 4) WY-14643 8day. The y-axis represents probes whose log-2 (fold change) value against the control group was 1.5 or more by 29day treatment of WY-14643.

Supplementary Table 1

L

	Grade	Degeneration, granular, eosinophilic	Necrosis	Single cell necrosis	Hypertrophy	Increased mitosis	Cell infiltration
		Hepatocyte	Hepatocyte	Hepatocyte	Bile duct, interlobular	Hepatocyte	Sinusoid
4 day	Minimal	0	0	0	0	3	0
	Slight	1	0	0	0	0	0
	Moderate	2	0	0	0	0	0
	Severe	0	0	0	0	0	0
8 day	Minimal	0	1	0	1	0	0
	Slight	0	0	0	0	0	0
	Moderate	0	0	0	0	0	0
	Severe	3	0	0	0	0	0
15 day	Minimal	0	1	0	1	0	0
	Slight	0	0	0	0	0	0
	Moderate	0	0	0	0	0	0
	Severe	3	0	0	0	0	0
29 day	Minimal	0	2	0	3	0	0
	Slight	0	0	0	0	0	0
	Moderate	0	0	0	0	0	0
	Severe	3	0	0	0	0	0

M

	Grade	Degeneration, granular, eosinophilic	Necrosis	Single cell necrosis	Hypertrophy	Increased mitosis	Cell infiltration
		Hepatocyte	Hepatocyte	Hepatocyte	Bile duct, interlobular	Hepatocyte	Sinusoid
4 day	Minimal	0	0	0	0	1	0
	Slight	0	0	0	0	2	0
	Moderate	3	0	0	0	0	0
	Severe	0	0	0	0	0	0
8 day	Minimal	0	0	1	2	0	0
	Slight	0	0	0	1	0	0
	Moderate	0	0	0	0	0	0
	Severe	3	0	0	0	0	0
15 day	Minimal	0	1	0	2	0	0
	Slight	0	0	0	1	0	0
	Moderate	0	0	0	0	0	0
	Severe	3	0	0	0	0	0
29 day	Minimal	0	1	0	1	0	0
	Slight	0	1	0	2	0	0
	Moderate	0	0	0	0	0	0
	Severe	3	0	0	0	0	0

H

	Grade	Degeneration, granular, eosinophilic	Necrosis	Single cell necrosis	Hypertrophy	Increased mitosis	Cell infiltration	Granuloma
		Hepatocyte	Hepatocyte	Hepatocyte	Bile duct, interlobular	Hepatocyte	Sinusoid	(−)
4 day	Minimal	0	1	1	0	1	2	0
	Slight	0	0	0	0	1	0	0
	Moderate	3	0	0	0	0	0	0
	Severe	0	0	0	0	0	0	0
8 day	Minimal	0	0	0	2	3	0	1
	Slight	0	0	0	1	0	0	0
	Moderate	0	0	0	0	0	0	0
	Severe	3	0	0	0	0	0	0
15 day	Minimal	0	1	0	1	0	0	0
	Slight	0	0	0	2	0	0	0
	Moderate	0	0	0	0	0	0	0
	Severe	3	0	0	0	0	0	0
29 day	Minimal	0	1	0	3	0	0	1
	Slight	0	0	0	0	0	0	0
	Moderate	0	0	0	0	0	0	0
	Severe	3	0	0	0	0	0	0

Supplementary Table 1 Pathology by repeated administration of WY-14643

WY-14643 was administered to rats (n=3) repeatedly for 4, 8, 15 or 29 days and the pathological observations were summarized. The numerical value in each cell of the table represents the number of rats that showed listed observation. L: 10 mg/kb BW, M: 30 mg/kg BW, H: 100 mg/kb BW. For the detailed information about the experimental conditions or the definitions of “Grade” of observations, refer to Open TG-GATEs (http://toxico.nibiohn.go.jp/english/).

Supplementary Table 2

M4 (434 probes)

cluster1 (244 probes)		
Pathways	p-value	matches
PPAR signaling pathway [rno03320]	3.97E-20	22
Fatty acid degradation [rno00071]	1.48E-19	18
Metabolism of lipids and lipoproteins [R-RNO-556833]	6.30E-17	39
Fatty acid metabolism [rno01212]	7.75E-14	15
Biosynthesis of unsaturated fatty acids [rno01040]	2.37E-13	12
IPC	p-value	matches
Metabolism of lipids and lipoproteins [R005]	1.16E-36	67
Glycerophospholipid metabolism|Arachidonic acid metabolism [R010]	2.12E-09	20
Biological oxidations|Chemical carcinogenesis [R006]	1.59E-04	17
Carbon metabolism [R009]	3.53E-03	11

cluster2 (113 probes)		
Cell Cycle [R-RNO-1640170]	6.93E-24	28
Cell Cycle, Mitotic [R-RNO-69278]	6.93E-24	27
M Phase [R-RNO-68886]	1.38E-15	17
Mitotic Prometaphase [6.17e-15]	6.17E-15	14
Cell Cycle [rno04110]	2.63E-14	15
IPC	p-value	matches
Cell Cycle [R007]	1.62E-25	34
Programmed Cell Death|p53 signaling pathway [R018]	5.88E-06	10
Generic Transcription Pathway [R019]	0.03	8

cluster3 (77 probes)		
Steroid hormone biosynthesis [rno00140]	8.25E-03	5
IPC	p-value	matches
Biological oxidations|Chemical carcinogenesis [R006]	3.38E-02	6
Glycerophospholipid metabolism|Arachidonic acid metabolism [R010]	3.38E-02	5

M8 (325 probes)

cluster1 (202 probes)		
Pathways	p-value	matches
PPAR signaling pathway [rno03320]	5.39E-22	22
Fatty acid degradation [rno00071]	1.62E-17	16
Metabolism of lipids and lipoproteins [R-RNO-556833]	3.03E-15	34
Biosynthesis of unsaturated fatty acids [rno01040]	3.53E-14	12
Metabolic pathways [rno01100]	1.91E-13	49
IPC	p-value	matches
Metabolism of lipids and lipoproteins [R005]	3.00E-36	61
Glycerophospholipid metabolism|Arachidonic acid metabolism [R010]	5.29E-09	18
Biological oxidations|Chemical carcinogenesis [R006]	2.23E-06	18
Carbon metabolism [R009]	6.67E-04	11
Pantothenate and CoA biosynthesis [R008]	1.89E-02	3

cluster2 (123 probes)		
Steroid hormone biosynthesis [rno00140]	3.65E-04	7
Bile secretion [rno04976]	0.000365	7
Metabolic pathways [rno01100]	0.00193	22
Metabolism [R-RNO-1430728]	4.29E-03	22
Retinol metabolism [rno00830]	4.29E-03	6
IPC	p-value	matches
Biological oxidations|Chemical carcinogenesis [R006]	6.99E-06	13
Glycerophospholipid metabolism|Arachidonic acid metabolism [R010]	6.99E-06	11
Metabolism of lipids and lipoproteins [R005]	0.00376	14
Transmembrane transport of small molecules [R015]	0.00376	12
Amimo acid and derivative metabolism [R015]	0.01	8

M15 (420 probes)

cluster1 (239 probes)		
Pathways	p-value	matches
PPAR signaling pathway [rno03320]	2.20E-21	22
Metabolism of lipids and lipoproteins [R-RNO-556833]	5.05E-16	36
Fatty acid degradation [rno00071]	6.14E-14	14
Biosynthesis of unsaturated fatty acids [rno01040]	7.40E-14	12
Metabolism [R-RNO-1430728]	1.44E-13	53
IPC	p-value	matches
Metabolism of lipids and lipoproteins [R005]	3.78E-33	60
Glycerophospholipid metabolism|Arachidonic acid metabolism [R010]	1.77E-09	19
Biological oxidations|Chemical carcinogenesis [R006]	3.19E-08	21
Carbon metabolism [R009]	5.43E-03	10

cluster2 (181 probes)		
Steroid hormone biosynthesis [rno00140]	9.06E-05	9
Chemical carcinogenesis [rno05204]	0.0195	7
Metabolic pathways [rno01100]	0.0263	27
Bile secretion [rno04976]	0.0263	6
ABC transporters [rno02010]	0.0277	5
IPC	p-value	matches
Biological oxidations|Chemical carcinogenesis [R006]	1.04E-04	15
ABC transporters [R062]	3.61E-03	5
Glycerophospholipid metabolism|Arachidonic acid metabolism [R010]	1.08E-02	9
Complement and coagulation cascades [R031]	3.85E-02	5
Amimo acid and derivative metabolism [R015]	4.20E-02	13

M29 (399 probes)

cluster1 (237 probes)		
Pathways	p-value	matches
PPAR signaling pathway [rno03320]	9.83E-21	22
Fatty acid degradation [rno00071]	5.74E-15	15
Metabolism of lipids and lipoproteins [R-RNO-556833]	2.72E-14	35
Biosynthesis of unsaturated fatty acids [rno01040]	1.63E-13	12
Fatty acid metabolism [rno01212]	8.18E-13	14
IPC	p-value	matches
Metabolism of lipids and lipoproteins [R005]	4.21E-30	59
Glycerophospholipid metabolism|Arachidonic acid metabolism [R010]	5.57E-09	19
Biological oxidations|Chemical carcinogenesis [R006]	1.06E-07	21
Carbon metabolism [R009]	9.07E-03	10

cluster2 (162 probes)		
Steroid hormone biosynthesis [rno00140]	1.68E-06	10
Chemical carcinogenesis [rno05204]	5.03E-05	9
Metabolic pathways [rno01100]	6.84E-05	30
Metabolism [R-RNO-1430728]	1.00E-03	29
Retinol metabolism [rno00830]	0.00207	7
IPC	p-value	matches
Biological oxidations|Chemical carcinogenesis [R006]	7.74E-08	18
Glycerophospholipid metabolism|Arachidonic acid metabolism [R010]	1.28E-03	10
Complement and coagulation cascades [R031]	3.88E-03	6
Amimo acid and derivative metabolism [R015]	2.98E-02	9
Metabolism of lipids and lipoproteins [R005]	3.57E-02	15

Test correction: Benjamini Hochberg
cutoff for dendrogram: 4

Supplementary Table S2 Enriched pathways in genes whose expression intensities were affected by repeated administration of WY-14643

Probes whose log2 (fold change) value against the control group was 1.5 or more were filtered, and were divided into clusters by interactive heatmap function on Toxygates (method: ward.D2, distance: pearson, cutoff for dendrogram: 4, test correction for enrichment analysis: Benjamini Hochberg, cutoff for enrichment analysis: p=0.05). Only the top 5 enriched pathways or integrative pathway clusters (IPCs) are listed. The expression profiles of these probes are shown in Supplementary Figure S1.

Supplementary Table S3

Gene list of “WY-downregulated genes”
Genes whose middle-dose expression value was half of or less than that of the corresponding control group
at all the time points (4, 8, 15, and 29 days) were used for compound ranking to discover similar compounds
to WY-14643 in terms of the downregulation of genes.

100362572	LOC100362572	Mpv17 transgene, kidney disease mutant-like (predicted)-like	Rattus norvegicus
100362769	Mt1f	metallothionein 1F	Rattus norvegicus
100364391	LOC100364391	dehydrogenase/reductase (SDR family) member 7-like	Rattus norvegicus
100911116	LOC100911116	3 beta-hydroxysteroid dehydrogenase type 5-like	Rattus norvegicus
114027	Dao	D-amino-acid oxidase	Rattus norvegicus
116663	Dusp6	dual specificity phosphatase 6	Rattus norvegicus
117522	Xpnpep2	X-prolyl aminopeptidase 2	Rattus norvegicus
155192	Abcg8	ATP binding cassette subfamily G member 8	Rattus norvegicus
171352	Cyp3a9	cytochrome P450, family 3, subfamily a, polypeptide 9	Rattus norvegicus
171380	Cyp2t1	cytochrome P450, family 2, subfamily t, polypeptide 1	Rattus norvegicus
171385	Acmsd	aminocarboxymuconate semialdehyde decarboxylase	Rattus norvegicus
171522	Cyp2d4	cytochrome P450, family 2, subfamily d, polypeptide 4	Rattus norvegicus
192247	Sez6	seizure related 6 homolog	Rattus norvegicus
24237	C6	complement C6	Rattus norvegicus
24297	Cyp1a2	cytochrome P450, family 1, subfamily a, polypeptide 2	Rattus norvegicus
24326	Ednra	endothelin receptor type A	Rattus norvegicus
24470	Hsd3b5	hydroxy-delta-5-steroid dehydrogenase, 3 beta- and steroid delta-isomerase 5	Rattus norvegicus
24567	Mt1	metallothionein 1	Rattus norvegicus
246142	Bmf	Bcl2 modifying factor	Rattus norvegicus
24626	Pde4b	phosphodiesterase 4B	Rattus norvegicus
24651	Pklr	pyruvate kinase, liver and RBC	Rattus norvegicus
24856	Ttr	transthyretin	Rattus norvegicus
24914	Lox	lysyl oxidase	Rattus norvegicus
25044	Sds	serine dehydratase	Rattus norvegicus
25080	Apoa4	apolipoprotein A4	Rattus norvegicus
25116	Hsd11b1	hydroxysteroid 11-beta dehydrogenase 1	Rattus norvegicus
25256	Fmo1	flavin containing monooxygenase 1	Rattus norvegicus
25559	Abcc8	ATP binding cassette subfamily C member 8	Rattus norvegicus
25560	Abcc9	ATP binding cassette subfamily C member 9	Rattus norvegicus
25698	Ass1	argininosuccinate synthase 1	Rattus norvegicus
286954	Ugt2b1	UDP glucuronosyltransferase 2 family, polypeptide	

B1 Rattus norvegicus
289827 Ugp2 UDP-glucose pyrophosphorylase 2 Rattus norvegicus
29276 Csrp1 cysteine and glycine-rich protein 1 Rattus norvegicus
29277 Cyp2c11 cytochrome P450, subfamily 2, polypeptide 11 Rattus norvegicus
29301 Hal histidine ammonia lyase Rattus norvegicus
293688 Tm7sf2 transmembrane 7 superfamily member 2 Rattus norvegicus
294228 RT1-S3 RT1 class Ib, locus S3 Rattus norvegicus
29758 St6galnac3 ST6 N-acetylgalactosaminide alpha-2,6-sialyltransferase 3 Rattus norvegicus
298079 Aldh1b1 aldehyde dehydrogenase 1 family, member B1 Rattus norvegicus
298107 Mup5 major urinary protein 5 Rattus norvegicus
299131 Dhrs7 dehydrogenase/reductase (SDR family) member 7 Rattus norvegicus
300993 Hyal3 hyaluronoglucosaminidase 3 Rattus norvegicus
303630 Wipi1 WD repeat domain, phosphoinositide interacting 1 Rattus norvegicus
303638 Abca8a ATP-binding cassette, subfamily A (ABC1), member 8a Rattus norvegicus
313610 Extl1 exostosin-like glycosyltransferase 1 Rattus norvegicus
314675 Btbd11 BTB domain containing 11 Rattus norvegicus
315084 Gsdmd gasdermin D Rattus norvegicus
360406 Ptprf protein tyrosine phosphatase, receptor type, F Rattus norvegicus
360718 Popdc2 popeye domain containing 2 Rattus norvegicus
361730 Tkfc triokinase and FMN cyclase Rattus norvegicus
362019 RGD1310209 similar to KIAA1324 protein Rattus norvegicus
362895 Stac3 SH3 and cysteine rich domain 3 Rattus norvegicus
368066 Inmt indolethylamine N-methyltransferase Rattus norvegicus
497811 Xdh xanthine dehydrogenase Rattus norvegicus
498545 Tsc22d1 TSC22 domain family, member 1 Rattus norvegicus
59114 Slc9a3r1 SLC9A3 regulator 1 Rattus norvegicus
63840 Per2 period circadian clock 2 Rattus norvegicus
64570 Nat8 N-acetyltransferase 8 Rattus norvegicus
680875 LOC680875 similar to dystonin isoform 1 Rattus norvegicus
684841 LOC684841 similar to CG31613-PA Rattus norvegicus
83500 Slc22a8 solute carrier family 22 member 8 Rattus norvegicus
84386 Slpi secretory leukocyte peptidase inhibitor Rattus norvegicus
85431 Nox4 NADPH oxidase 4 Rattus norvegicus
89787 Lrp3 LDL receptor related protein 3 Rattus norvegicus

Supplementary Table 4

M4 vs M8 (t-test, p-value<0.01)

cluster1 (268 probes)		
Pathways	p-value	matches
Beta-catenin independent WNT signaling [R-RNO-3858494]	0.000955	9
PCP/CE pathway [R-RNO-4086400]	0.000955	8
Proteasome [mo03050]	0.00128	7
Cross-presentation of soluble exogeneous antigens (endosomes) [R-RNO-1236978]	0.00261	6
Regulation of ornithine decarboxylase (ODC) [R-RNO-350562]	0.00261	6
AUF1 (hnRNP D0) binds and destabilizes mRNA [R-RNO-450408]	0.00261	6
Degradation of GLI1 by the proteasome [R-RNO-5610780]	0.00261	6
GLI3 is processed to GLI3R by the proteasome [R-RNO-5610785]	0.00261	6
Ribosome [rno03010]	0.00261	11
IPC	p-value	matches
Ribosome [R021]	0.00173	15
Organelle biogenesis and maintenance [R046]	0.0114	11
Amino acid and derivative metabolism [R008]	0.0264	12

cluster2 (188 probes)		
Pathways	p-value	matches
–	–	–
IPC	p-value	matches
Protein processing in endoplasmic reticulum [R028]	0.00193	9
Complement and coagulation cascades [R031]	0.00419	6

cluster3 (162 probes)		
Pathways	p-value	matches
Cam-PDE1 activation [R-RNO-111957]	0.000483	3
Ca2+ pathway [R-RNO-4086398]	0.000483	5
Activation of CaMK IV [R-RNO-442745]	0.000483	3
Activation of Kainate Receptors upon glutamate binding [R-RNO-451326]	0.000483	4
Glycogen breakdown (glycogenolysis) [R-RNO-70221]	0.000483	4
IPC	p-value	matches
Carbohydrate metabolism [R029]	0.000372	10

Supplementary Table 4

Enriched pathways in genes whose expression intensities were significantly different between M4 and M8

Probes whose log2 (fold change) value in M4 against that in M8 was significantly different by student's t-test (cutoff o-value: 0.01) were filtered, and were divided into clusters by interactive heatmap function on Toxygates (method: ward.D2, distance: pearson, cutoff for dendrogram: 6, test correction for enrichment analysis: Benjamini Hochberg, cutoff for enrichment analysis: p=0.05). Only the top 5 enriched pathways or integrative pathway clusters (IPCs) are listed. The expression profiles of these probes are shown in Figure 2.

Supplementary Table 5

Gene list of “M4vs8_cluster1”
This cluster was obtained by hierarchical clustering (dendrogram cutoff: 6) of DEGs between
WY-14643/M dose/4day and WY-14643/M dose/8day by student’s t-test (cutoff: p-value=0.01).
The cluster that contained 268 probes was defined as “M4vs8_cluster1”.

100125373 Pnrc2 proline-rich nuclear receptor coactivator 2 Rattus norvegicus
100359945 Rslcan18 regulator of sex-limitation candidate 18 Rattus norvegicus
100360117 LOC100360117 ribosomal protein L8-like Rattus norvegicus
100360522 LOC100360522 ribosomal protein P1-like Rattus norvegicus
100360682 Snrpe small nuclear ribonucleoprotein polypeptide E Rattus norvegicus
100360846 LOC100360846 proteasome subunit beta type 6-like Rattus norvegicus
100361269 Snrpa1 small nuclear ribonucleoprotein polypeptide A' Rattus norvegicus
100362333 LOC100362333 DNA-directed DNA polymerase epsilon 3 Rattus norvegicus
100362980 LOC100362980 CG3918-like Rattus norvegicus
100363012 Rps21-ps1 ribosomal protein S21, pseudogene 1 Rattus norvegicus
100363642 LOC100363642 coiled-coil domain-containing protein 41-like Rattus norvegicus
100365711 LOC100365711 cytochrome c oxidase, subunit VIc-like Rattus norvegicus
100910056 LOC100910056 UHRF1-binding protein 1-like Rattus norvegicus
100910130 LOC100910130 protein FAM50A-like Rattus norvegicus
100910370 LOC100910370 60S ribosomal protein L8-like Rattus norvegicus
100911372 LOC100911372 40S ribosomal protein S6-like Rattus norvegicus
100911847 LOC100911847 40S ribosomal protein S14-like Rattus norvegicus
100912469 LOC100912469 acyl-CoA desaturase 2-like Rattus norvegicus
100912578 LOC100912578 stress-70 protein, mitochondrial-like Rattus norvegicus
114508 Fbp2 fructose-bisphosphatase 2 Rattus norvegicus
114517 Itga6 integrin subunit alpha 6 Rattus norvegicus
114764 Mapre1 microtubule-associated protein, RP/EB family, member 1 Rattus norvegicus
116561 Cltb clathrin, light chain B Rattus norvegicus
116656 Cdc123 cell division cycle 123 Rattus norvegicus

117557	Tpm3	tropomyosin 3	Rattus norvegicus
140661	Rplp1	ribosomal protein, large, P1	Rattus norvegicus
140922	Txnl1	thioredoxin-like 1	Rattus norvegicus
140932	Bnip1	BCL2/adenovirus E1B interacting protein 1	Rattus norvegicus
140937	Higd1a	HIG1 hypoxia inducible domain family, member 1A	Rattus norvegicus
170900	Rbm14	RNA binding motif protein 14	Rattus norvegicus
171085	Pcsk4	proprotein convertase subtilisin/kexin type 4	Rattus norvegicus
171142	Ehhadh	enoyl-CoA hydratase and 3-hydroxyacyl CoA dehydrogenase	Rattus norvegicus
171178	Adh7	alcohol dehydrogenase 7 (class IV), mu or sigma polypeptide	Rattus norvegicus
171497	Sgk2	serum/glucocorticoid regulated kinase 2	Rattus norvegicus
192362	Lamc2	laminin subunit gamma 2	Rattus norvegicus
24157	Acaa1a	acetyl-CoA acyltransferase 1	Rattus norvegicus
24188	Aldh1a1	aldehyde dehydrogenase 1 family, member A1	Rattus norvegicus
24400	Gnb1	G protein subunit beta 1	Rattus norvegicus
246282	Zfp91	zinc finger protein 91	Rattus norvegicus
24644	Pgk1	phosphoglycerate kinase 1	Rattus norvegicus
24673	Ppp2cb	protein phosphatase 2 catalytic subunit beta	Rattus norvegicus
24818	Tcp1	t-complex 1	Rattus norvegicus
25098	Foxa1	forkhead box A1	Rattus norvegicus
25151	Igf2r	insulin-like growth factor 2 receptor	Rattus norvegicus
252855	Sfpq	splicing factor proline and glutamine rich	Rattus norvegicus
252899	Miox	myo-inositol oxygenase	Rattus norvegicus
25344	Phb	prohibitin	Rattus norvegicus
25402	Casp3	caspase 3	Rattus norvegicus
25403	Cast	calpastatin	Rattus norvegicus
25405	Ccng1	cyclin G1	Rattus norvegicus
25458	Gss	glutathione synthetase	Rattus norvegicus
25579	Map3k12	mitogen activated protein kinase kinase kinase 12	Rattus norvegicus
25604	Pcmt1	protein-L-isoaspartate (D-aspartate) O-methyltransferase 1	Rattus norvegicus
25612	Asns	asparagine synthetase (glutamine-hydrolyzing)	Rattus norvegicus
260321	Fkbp4	FK506 binding protein 4	Rattus norvegicus
266631	Rbm45	RNA binding motif protein 45	Rattus norvegicus
26962	Rpl8	ribosomal protein L8	Rattus norvegicus
282584	Mtmr9	myotubularin related protein 9	Rattus norvegicus
286894	Parm1	prostate androgen-regulated mucin-like protein 1	Rattus norvegicus
287061	Rogdi	rogdi homolog	Rattus norvegicus
287120	Tbl3	transducin (beta)-like 3	Rattus norvegicus
287155	Stub1	STIP1 homology and U-box containing protein 1	Rattus norvegicus
287367	Cops3	COP9 signalosome subunit 3	Rattus norvegicus

287420 Pfas phosphoribosylformylglycinamidine synthase Rattus norvegicus
287444 Eif5a eukaryotic translation initiation factor 5A Rattus norvegicus
287927 Ube2v2 ubiquitin conjugating enzyme E2 V2 Rattus norvegicus
288065 Ccdc58 coiled-coil domain containing 58 Rattus norvegicus
288471 Trrap transformation/transcription domain-associated protein Rattus norvegicus
288599 Eif4h eukaryotic translation initiation factor 4H Rattus norvegicus
289125 Acbd6 acyl-CoA binding domain containing 6 Rattus norvegicus
289150 Cenpl centromere protein L Rattus norvegicus
289990 Psmc6 proteasome 26S subunit, ATPase 6 Rattus norvegicus
290027 Parp2 poly (ADP-ribose) polymerase 2 Rattus norvegicus
290291 Cab39l calcium binding protein 39-like Rattus norvegicus
290303 Spryd7 SPRY domain containing 7 Rattus norvegicus
290401 Esd esterase D Rattus norvegicus
290529 Fam213a family with sequence similarity 213, member A Rattus norvegicus
291005 Gadd45g growth arrest and DNA-damage-inducible, gamma Rattus norvegicus
291034 Mcur1 mitochondrial calcium uniporter regulator 1 Rattus norvegicus
291671 Hspa9 heat shock protein family A member 9 Rattus norvegicus
292087 Urb2 URB2 ribosome biogenesis 2 homolog (S. cerevisiae) Rattus norvegicus
292156 Sh3glb1 SH3 domain -containing GRB2-like endophilin B1 Rattus norvegicus
292306 Rnaset2 ribonuclease T2 Rattus norvegicus
292485 Fuca2 fucosidase, alpha-L- 2, plasma Rattus norvegicus
29261 Tyms thymidylate synthetase Rattus norvegicus
292777 Tbcb tubulin folding cofactor B Rattus norvegicus
29284 Rps14 ribosomal protein S14 Rattus norvegicus
292887 Akt1s1 AKT1 substrate 1 Rattus norvegicus
29304 Rps6 ribosomal protein S6 Rattus norvegicus
293113 Tmem126a transmembrane protein 126A Rattus norvegicus
293149 Mrpl48 mitochondrial ribosomal protein L48 Rattus norvegicus
29317 Csrp2 cysteine and glycine-rich protein 2 Rattus norvegicus
293481 Tufm Tu translation elongation factor, mitochondrial Rattus norvegicus
293613 RGD1309350 similar to transthyretin (4L369) Rattus norvegicus
29384 H2afy H2A histone family, member Y Rattus norvegicus
293862 Fam50a family with sequence similarity 50, member A Rattus norvegicus
293888 Plgrkt plasminogen receptor with a C-terminal lysine Rattus norvegicus
294009 Cuedc2 CUE domain containing 2 Rattus norvegicus
294339 Mcm3ap minichromosome maintenance complex component 3

associated protein	Rattus norvegicus
295088	Gmps	guanine monophosphate synthase	Rattus norvegicus
295171	Sh3d19	SH3 domain containing 19	Rattus norvegicus
295500	Metap1	methionyl aminopeptidase 1	Rattus norvegicus
295692	Nup35	nucleoporin 35	Rattus norvegicus
296570	Edf1	endothelial differentiation-related factor 1
Rattus norvegicus
296658	Ndufa8	NADH:ubiquinone oxidoreductase subunit A8	Rattus norvegicus
29666	Psmb6	proteasome subunit beta 6	Rattus norvegicus
29674	Psma7	proteasome subunit alpha 7	Rattus norvegicus
296911	ST7	suppression of tumorigenicity 7	Rattus norvegicus
296913	Lsm8	LSM8 homolog, U6 small nuclear RNA associated
Rattus norvegicus
29728	Mcm4	minichromosome maintenance complex component 4
Rattus norvegicus
297372	Mrpl19	mitochondrial ribosomal protein L19	Rattus norvegicus
297393	Nagk	N-acetylglucosamine kinase	Rattus norvegicus
297627	Rhno1	RAD9-HUS1-RAD1 interacting nuclear orphan 1
Rattus norvegicus
297694	Plbd1	phospholipase B domain containing 1	Rattus norvegicus
297727	Mrps35	mitochondrial ribosomal protein S35	Rattus norvegicus
298098	Pole3	polymerase (DNA directed), epsilon 3, accessory subunit	Rattus norvegicus
298441	Nasp	nuclear autoantigenic sperm protein	Rattus norvegicus
298648	Tardbp	TAR DNA binding protein	Rattus norvegicus
298914	Itgb1bp1	integrin subunit beta 1 binding protein 1	Rattus norvegicus
298917	Iah1	isoamyl acetate-hydrolyzing esterase 1 homolog
Rattus norvegicus
299041	RGD1565054	similar to 60S acidic ribosomal protein P1
Rattus norvegicus
300120	Fam118a	family with sequence similarity 118, member A
Rattus norvegicus
300447	Ecsit	ECSIT signalling integrator	Rattus norvegicus
300652	Sorl1	sortilin related receptor 1	Rattus norvegicus
300891	Morf4l1	mortality factor 4 like 1	Rattus norvegicus
301337	Plekhb2	pleckstrin homology domain containing B2	Rattus norvegicus
301381	Tex30	testis expressed 30	Rattus norvegicus
301529	Dnpep	aspartyl aminopeptidase	Rattus norvegicus
301622	Dtymk	deoxythymidylate kinase	Rattus norvegicus
302864	Mmgt1	membrane magnesium transporter 1	Rattus norvegicus
304881	Tor1aip2	torsin 1A interacting protein 2	Rattus norvegicus
304919	Dars2	aspartyl-tRNA synthetase 2 (mitochondrial)	Rattus norvegicus
305910	Pspc1	paraspeckle component 1	Rattus norvegicus
306182	Ipo5	importin 5	Rattus norvegicus
306542	Brf2	BRF2, RNA polymerase III transcription initiation factor 50 subunit	Rattus norvegicus

306628	Col4a2	collagen type IV alpha 2 chain	Rattus norvegicus
306994	Yae1d1	Yae1 domain containing 1	Rattus norvegicus
307091	Akr1e2	aldo-keto reductase family 1, member E2	Rattus norvegicus
307947	Set	SET nuclear proto-oncogene	Rattus norvegicus
308113	Cnksr3	Cnksr family member 3	Rattus norvegicus
308607	E2f8	E2F transcription factor 8	Rattus norvegicus
308652	Smox	spermine oxidase	Rattus norvegicus
308820	Ccdc90b	coiled-coil domain containing 90B	Rattus norvegicus
308871	Rnf121	ring finger protein 121	Rattus norvegicus
309098	Tubgcp2	tubulin, gamma complex associated protein 2	Rattus norvegicus
309176	Mrpl49	mitochondrial ribosomal protein L49	Rattus norvegicus
309570	Slc18b1	solute carrier family 18 member B1	Rattus norvegicus
310811	Palmd	palmdelphin	Rattus norvegicus
311078	Psmd14	proteasome 26S subunit, non-ATPase 14	Rattus norvegicus
311130	Prkra	protein activator of interferon induced protein kinase EIF2AK2	Rattus norvegicus
311191	Ckap5	cytoskeleton associated protein 5	Rattus norvegicus
311821	Agpat2	1-acylglycerol-3-phosphate O-acyltransferase 2	Rattus norvegicus
311855	RGD1305178	similar to Hypothetical protein MGC11690	Rattus norvegicus
311903	Mrrf	mitochondrial ribosome recycling factor	Rattus norvegicus
312299	Ezh2	enhancer of zeste 2 polycomb repressive complex 2 subunit	Rattus norvegicus
312824	Recql	RecQ like helicase	Rattus norvegicus
313108	Mms22l	MMS22-like, DNA repair protein	Rattus norvegicus
313647	Hp1bp3	heterochromatin protein 1, binding protein 3	Rattus norvegicus
313929	Ncoa1	nuclear receptor coactivator 1	Rattus norvegicus
314652	RGD1564744	similar to 60S acidic ribosomal protein P1	Rattus norvegicus
315150	Adsl	adenylosuccinate lyase	Rattus norvegicus
315649	Sik2	salt-inducible kinase 2	Rattus norvegicus
316348	Pdcl3	phosducin-like 3	Rattus norvegicus
316611	Agap1	ArfGAP with GTPase domain, ankyrin repeat and PH domain 1	Rattus norvegicus
317259	Nono	non-POU domain containing, octamer-binding	Rattus norvegicus
317464	Msl3	male-specific lethal 3 homolog (Drosophila)	Rattus norvegicus
360664	Ten1	TEN1 CST complex subunit	Rattus norvegicus
360915	Cops4	COP9 signalosome subunit 4	Rattus norvegicus
360950	Wdr1	WD repeat domain 1	Rattus norvegicus
361005	Chchd1	coiled-coil-helix-coiled-coil-helix domain containing 1	Rattus norvegicus
361191	Nsun2	NOP2/Sun RNA methyltransferase family, member 2	Rattus norvegicus
361301	Tpgs2	tubulin polyglutamylase complex subunit 2	Rattus

norvegicus
361452	Ltv1	LTV1 ribosome biogenesis factor	Rattus norvegicus
361510	Sult2a2	sulfotransferase family 2A, dehydroepiandrosterone (DHEA)-preferring, member 2	Rattus norvegicus
361624	Akip1	A-kinase interacting protein 1	Rattus norvegicus
361666	Bccip	BRCA2 and CDKN1A interacting protein	Rattus norvegicus
361821	Col6a2	collagen type VI alpha 2 chain	Rattus norvegicus
361888	Srek1ip1	SREK1-interacting protein 1	Rattus norvegicus
361956	Rsrc1	arginine and serine rich coiled-coil 1	Rattus norvegicus
362129	Gtdc1	glycosyltransferase-like domain containing 1	Rattus norvegicus
362152	Hnrnpa3	heterogeneous nuclear ribonucleoprotein A3	Rattus norvegicus
362156	Zdhhc5	zinc finger, DHHC-type containing 5	Rattus norvegicus
362228	Naa20	N(alpha)-acetyltransferase 20, NatB catalytic subunit	Rattus norvegicus
362419	Tamm41	TAM41 mitochondrial translocator assembly and maintenance homolog	Rattus norvegicus
362557	Ndc1	NDC1 transmembrane nucleoporin	Rattus norvegicus
362626	Rsrp1	arginine and serine rich protein 1	Rattus norvegicus
362905	Emc2	ER membrane protein complex subunit 2	Rattus norvegicus
362943	Adck5	aarF domain containing kinase 5	Rattus norvegicus
362972	Pnpla3	patatin-like phospholipase domain containing 3	Rattus norvegicus
363009	Uhrf1bp1l	UHRF1 binding protein 1-like	Rattus norvegicus
363061	Sdhd	succinate dehydrogenase complex subunit D	Rattus norvegicus
363068	Commd4	COMM domain containing 4	Rattus norvegicus
363334	Stap2	signal transducing adaptor family member 2	Rattus norvegicus
364227	Asb3	ankyrin repeat and SOCS box-containing 3	Rattus norvegicus
366474	Col16a1	collagen type XVI alpha 1 chain	Rattus norvegicus
366595	Sypl1	synaptophysin-like 1	Rattus norvegicus
366872	Cep83	centrosomal protein 83	Rattus norvegicus
406195	Tcf19	transcription factor 19	Rattus norvegicus
494445	Srsf2	serine and arginine rich splicing factor 2	Rattus norvegicus
499374	RGD1561333	similar to 60S ribosomal protein L8	Rattus norvegicus
500006	Pex1	peroxisomal biogenesis factor 1	Rattus norvegicus
500028	LOC500028	hypothetical protein LOC500028	Rattus norvegicus
500616	Socs5	suppressor of cytokine signaling 5	Rattus norvegicus
501072	Acaa1b	acetyl-Coenzyme A acyltransferase 1B	Rattus norvegicus
501620	RGD1564480	similar to polyamine oxidase isoform 2

Rattus norvegicus
50556 Exoc6 exocyst complex component 6 Rattus norvegicus
50559 Acot1 acyl-CoA thioesterase 1 Rattus norvegicus
54322 Cox6c cytochrome c oxidase subunit 6C Rattus norvegicus
56785 Ralgapa1 Ral GTPase activating protein catalytic alpha subunit 1 Rattus norvegicus
58823 Nckap1 NCK-associated protein 1 Rattus norvegicus
58940 H2afz H2A histone family, member Z Rattus norvegicus
59317 Epb41l1 erythrocyte membrane protein band 4.1-like 1 Rattus norvegicus
619440 Aarsd1 alanyl-tRNA synthetase domain containing 1 Rattus norvegicus
64044 Casp8 caspase 8 Rattus norvegicus
64372 Timm8b translocase of inner mitochondrial membrane 8 homolog B Rattus norvegicus
64526 Ech1 enoyl-CoA hydratase 1 Rattus norvegicus
64539 Ndufv3 NADH:ubiquinone oxidoreductase subunit V3 Rattus norvegicus
64667 Sgta small glutamine rich tetratricopeptide repeat containing alpha Rattus norvegicus
65169 Scamp3 secretory carrier membrane protein 3 Rattus norvegicus
654495 Zdhhc16 zinc finger, DHHC-type containing 16 Rattus norvegicus
680039 LOC680039 hypothetical protein LOC680039 Rattus norvegicus
680747 Mrpl20 mitochondrial ribosomal protein L20 Rattus norvegicus
680835 LOC680835 similar to cullin 7 Rattus norvegicus
681062 Slbp stem-loop binding protein Rattus norvegicus
683687 Snx9 sorting nexin 9 Rattus norvegicus
684270 LOC684270 similar to isochorismatase domain containing 2 Rattus norvegicus
684830 Ttc32 tetratricopeptide repeat domain 32 Rattus norvegicus
685059 Msrb1 methionine sulfoxide reductase B1 Rattus norvegicus
686032 Smlr1 small leucine-rich protein 1 Rattus norvegicus
687118 Dedd2 death effector domain containing 2 Rattus norvegicus
687575 U2af1 U2 small nuclear RNA auxiliary factor 1 Rattus norvegicus
688815 LOC688815 similar to prohibitin Rattus norvegicus
689176 Tmem64 transmembrane protein 64 Rattus norvegicus
690085 Bcl7a BCL tumor suppressor 7A Rattus norvegicus
690349 Lbhd1 LBH domain containing 1 Rattus norvegicus
690441 Atp5j2 ATP synthase, H+ transporting, mitochondrial Fo complex, subunit F2 Rattus norvegicus
691501 Snapc5 small nuclear RNA activating complex, polypeptide 5 Rattus norvegicus
79227 Thoc6 THO complex 6 Rattus norvegicus
79242 Hpgd hydroxyprostaglandin dehydrogenase 15 (NAD) Rattus norvegicus
79243 Hsd17b2 hydroxysteroid (17-beta) dehydrogenase 2 Rattus norvegicus

81530	Pdk2	pyruvate dehydrogenase kinase 2	Rattus norvegicus
81684	Mipep	mitochondrial intermediate peptidase	Rattus norvegicus
81775	Rps21	ribosomal protein S21	Rattus norvegicus
81827	Psmc5	proteasome 26S subunit, ATPase 5	Rattus norvegicus
83422	Pias2	protein inhibitor of activated STAT, 2	Rattus norvegicus
83614	Pias3	protein inhibitor of activated STAT, 3	Rattus norvegicus
83792	Scd2	stearoyl-Coenzyme A desaturase 2	Rattus norvegicus
83800	Clta	clathrin, light chain A	Rattus norvegicus
84389	Ccnh	cyclin H	Rattus norvegicus
84575	Fads1	fatty acid desaturase 1	Rattus norvegicus
84581	Hdac6	histone deacetylase 6	Rattus norvegicus
85492	Psmb7	proteasome subunit beta 7	Rattus norvegicus

Supplementary Table 6

Gene list of “M4vs8_cluster2”
This cluster was obtained by hierarchical clustering (dendrogram cutoff: 6) of DEGs between
WY-14643/M dose/4day and WY-14643/M dose/8day by student’s t-test (cutoff: p-value=0.01).
The cluster that contained 188 probes was defined as “M4vs8_cluster2”.

100360218 LOC100360218 interleukin 13 receptor, alpha 1-like Rattus norvegicus
100362495 Haus5 HAUS augmin-like complex, subunit 5 Rattus norvegicus
100363266 Igip IgA-inducing protein Rattus norvegicus
100363500 LOC100363500 hypothetical LOC100363500 Rattus norvegicus
100911986 LOC100911986 zinc finger protein GLI4-like Rattus norvegicus
100912615 LOC100912615 transmembrane protein 19-like Rattus norvegicus
113961 Gorasp2 golgi reassembly stacking protein 2 Rattus norvegicus
114002 Ndst2 N-deacetylase and N-sulfotransferase 2 Rattus norvegicus
116782 Pcdhgc3 protocadherin gamma subfamily C, 3 Rattus norvegicus
117517 C7 complement C7 Rattus norvegicus
117556 Sv2b synaptic vesicle glycoprotein 2b Rattus norvegicus
154985 Cyp26a1 cytochrome P450, family 26, subfamily a, polypeptide 1 Rattus norvegicus
170699 Atp2c1 ATPase secretory pathway Ca2+ transporting 1 Rattus norvegicus
171070 Ptpn21 protein tyrosine phosphatase, non-receptor type 21 Rattus norvegicus
171563 Nav2 neuron navigator 2 Rattus norvegicus
192242 Akr1d1 aldo-keto reductase family 1, member D1 Rattus norvegicus
192262 C1s complement C1s Rattus norvegicus
24184 Ak2 adenylate kinase 2 Rattus norvegicus
24236 C4bpb complement component 4 binding protein, beta Rattus norvegicus
24312 Dhfr dihydrofolate reductase Rattus norvegicus
24413 Nr3c1 nuclear receptor subfamily 3, group C, member 1 Rattus norvegicus
24421 Gsta1 glutathione S-transferase alpha 1 Rattus norvegicus
246284 Atrx ATRX, chromatin remodeler Rattus norvegicus
24718 Reln reelin Rattus norvegicus
24852 Tpm4 tropomyosin 4 Rattus norvegicus
24904 Slc22a1 solute carrier family 22 member 1 Rattus norvegicus
24908 Dnajb9 DnaJ heat shock protein family (Hsp40) member B9 Rattus norvegicus
25043 Eln elastin Rattus norvegicus

25087	Kng2	kininogen 2	Rattus norvegicus
25114	Fgfr4	fibroblast growth factor receptor 4	Rattus norvegicus
252895	Pcdhga9	protocadherin gamma subfamily A, 9	Rattus norvegicus
252897	Pcdhga11	protocadherin gamma subfamily A, 11	Rattus norvegicus
252963	Il13ra1	interleukin 13 receptor subunit alpha 1	Rattus norvegicus
25308	Cstb	cystatin B	Rattus norvegicus
25374	Alad	aminolevulinate dehydratase	Rattus norvegicus
25423	Ctsc	cathepsin C	Rattus norvegicus
25506	P4hb	prolyl 4-hydroxylase subunit beta	Rattus norvegicus
25587	Id2	inhibitor of DNA binding 2, HLH protein	Rattus norvegicus
25596	Rpn1	ribophorin I	Rattus norvegicus
25603	Marcks	myristoylated alanine rich protein kinase C substrate	Rattus norvegicus
25617	Hspa5	heat shock protein family A member 5	Rattus norvegicus
25636	Prkaca	protein kinase cAMP-activated catalytic subunit alpha	Rattus norvegicus
25642	Cyp3a23/3a1	cytochrome P450, family 3, subfamily a, polypeptide 23/polypeptide 1	Rattus norvegicus
266689	Cyp4f6	cytochrome P450, family 4, subfamily f, polypeptide 6	Rattus norvegicus
287441	Zbtb4	zinc finger and BTB domain containing 4	Rattus norvegicus
287479	Shpk	sedoheptulokinase	Rattus norvegicus
287607	Mtmr4	myotubularin related protein 4	Rattus norvegicus
287731	Lsm12	LSM12 homolog	Rattus norvegicus
288124	Nectin3	nectin cell adhesion molecule 3	Rattus norvegicus
288692	Wsb2	WD repeat and SOCS box-containing 2	Rattus norvegicus
288783	Ormdl2	ORMDL sphingolipid biosynthesis regulator 2	Rattus norvegicus
289372	Smyd2	SET and MYND domain containing 2	Rattus norvegicus
290363	Rcbtb2	RCC1 and BTB domain containing protein 2	Rattus norvegicus
290642	Ccdc124	coiled-coil domain containing 124	Rattus norvegicus
291403	Zadh2	zinc binding alcohol dehydrogenase, domain containing 2	Rattus norvegicus
291440	Cxxc1	CXXC finger protein 1	Rattus norvegicus
291635	Pcdhga7	protocadherin gamma subfamily A, 7	Rattus norvegicus
291637	Pcdhga5	protocadherin gamma subfamily A, 5	Rattus norvegicus
293067	Zfand6	zinc finger AN1-type containing 6	Rattus norvegicus
29339	Apcs	amyloid P component, serum	Rattus norvegicus
293774	Dtx4	deltex E3 ubiquitin ligase 4	Rattus norvegicus
29439	Jtb	jumping translocation breakpoint	Rattus norvegicus
294410	Man1a1	mannosidase, alpha, class 1A, member 1	Rattus norvegicus
29468	Pdia3	protein disulfide isomerase family A, member 3

Rattus norvegicus
295231	Glmp	glycosylated lysosomal membrane protein	Rattus norvegicus
295674	Plekha3	pleckstrin homology domain containing A3	Rattus norvegicus
296554	Tubb4b	tubulin, beta 4B class IVb	Rattus norvegicus
298104	Tmem268	transmembrane protein 268	Rattus norvegicus
298370	Txndc12	thioredoxin domain containing 12	Rattus norvegicus
298757	Atl2	atlastin GTPase 2	Rattus norvegicus
298861	Ptrhd1	peptidyl-tRNA hydrolase domain containing 1	Rattus norvegicus
299732	Lta4h	leukotriene A4 hydrolase	Rattus norvegicus
299800	Tmem19	transmembrane protein 19	Rattus norvegicus
300115	Arhgap8	Rho GTPase activating protein 8	Rattus norvegicus
300674	Arcn1	archain 1	Rattus norvegicus
300955	Nck1	NCK adaptor protein 1	Rattus norvegicus
300981	Acy1	aminoacylase 1	Rattus norvegicus
301579	Armc9	armadillo repeat containing 9	Rattus norvegicus
301861	LOC301861	similar to ARP2 actin-related protein 2 homolog (yeast)	Rattus norvegicus
302898	Rsl1d1	ribosomal L1 domain containing 1	Rattus norvegicus
303563	Nags	N-acetylglutamate synthase	Rattus norvegicus
304577	Ung	uracil-DNA glycosylase	Rattus norvegicus
304929	F5	coagulation factor V	Rattus norvegicus
305156	Arhgap24	Rho GTPase activating protein 24	Rattus norvegicus
306630	Abhd13	abhydrolase domain containing 13	Rattus norvegicus
306647	Champ1	chromosome alignment maintaining phosphoprotein 1	Rattus norvegicus
308265	Lats1	large tumor suppressor kinase 1	Rattus norvegicus
310773	Cept1	choline/ethanolamine phosphotransferase 1	Rattus norvegicus
311021	Nmi	N-myc (and STAT) interactor	Rattus norvegicus
311332	Dll4	delta like canonical Notch ligand 4	Rattus norvegicus
311483	Rrbp1	ribosome binding protein 1	Rattus norvegicus
311637	Slc35c2	solute carrier family 35 member C2	Rattus norvegicus
312670	Adipor2	adiponectin receptor 2	Rattus norvegicus
312828	Etnk1	ethanolamine kinase 1	Rattus norvegicus
313961	Smc6	structural maintenance of chromosomes 6	Rattus norvegicus
314157	Fbxo33	F-box protein 33	Rattus norvegicus
315047	Laptm4b	lysosomal protein transmembrane 4 beta	Rattus norvegicus
315500	Eepd1	endonuclease/exonuclease/phosphatase family domain containing 1	Rattus norvegicus
315989	Manf	mesencephalic astrocyte-derived neurotrophic factor	Rattus norvegicus
316916	Clptm1l	CLPTM1-like	Rattus norvegicus
317599	Atp11c	ATPase phospholipid transporting 11C	Rattus norvegicus
361043	Tm9sf1	transmembrane 9 superfamily member 1	Rattus norvegicus
361050	Rcbtb1	RCC1 and BTB domain containing protein 1	Rattus

norvegicus
361213	Sptlc1	serine palmitoyltransferase, long chain base subunit 1	Rattus norvegicus
361261	Ccny	cyclin Y	Rattus norvegicus
361289	Colec12	collectin sub-family member 12	Rattus norvegicus
361749	Il33	interleukin 33	Rattus norvegicus
361991	S100a16	S100 calcium binding protein A16	Rattus norvegicus
362359	Mpp6	membrane palmitoylated protein 6	Rattus norvegicus
362472	Cspp1	centrosome and spindle pole associated protein 1	Rattus norvegicus
362861	Slc41a2	solute carrier family 41 member 2	Rattus norvegicus
362862	Hsp90b1	heat shock protein 90 beta family member 1	Rattus norvegicus
363159	Gpd1l	glycerol-3-phosphate dehydrogenase 1-like	Rattus norvegicus
363425	Cav2	caveolin 2	Rattus norvegicus
363989	Phlda3	pleckstrin homology-like domain, family A, member 3	Rattus norvegicus
364843	Pcdhga8	protocadherin gamma subfamily A, 8	Rattus norvegicus
364844	Pcdhgb7	protocadherin gamma subfamily B, 7	Rattus norvegicus
364845	Pcdhgb8	protocadherin gamma subfamily B, 8	Rattus norvegicus
365466	Cpn1	carboxypeptidase N subunit 1	Rattus norvegicus
366169	Rtf1	Rtf1, Paf1/RNA polymerase II complex component, homolog (S. cerevisiae)	Rattus norvegicus
366381	Cdc26	cell division cycle 26	Rattus norvegicus
474143	Clec4a	C-type lectin domain family 4, member A	Rattus norvegicus
498241	RGD1564614	similar to complement factor H-related protein	Rattus norvegicus
498846	Pcdhga2	protocadherin gamma subfamily A, 2	Rattus norvegicus
498847	Pcdhga3	protocadherin gamma subfamily A, 3	Rattus norvegicus
498849	Pcdhga10	protocadherin gamma subfamily A, 10	Rattus norvegicus
498850	Pcdhga12	protocadherin gamma subfamily A, 12	Rattus norvegicus
500039	RGD1562178	similar to Adenylate kinase 2	Rattus norvegicus
500893	Gli4	GLI family zinc finger 4	Rattus norvegicus
501083	Pdcd6ip	programmed cell death 6 interacting protein	Rattus norvegicus
501569	Tmem47	transmembrane protein 47	Rattus norvegicus
553129	Pcdhga1	protocadherin gamma subfamily A, 1	Rattus norvegicus
56759	Rnase4	ribonuclease A family member 4	Rattus norvegicus
56766	Leprot	leptin receptor overlapping transcript	Rattus norvegicus
58838	Sh2b3	SH2B adaptor protein 3	Rattus norvegicus
60423	Slc28a2	solute carrier family 28 member 2	Rattus norvegicus
64014	Dusp12	dual specificity phosphatase 12	Rattus norvegicus

64185 Cap1 adenylate cyclase associated protein 1 Rattus norvegicus
64202 Calr calreticulin Rattus norvegicus
64367 Ppib peptidylprolyl isomerase B Rattus norvegicus
64552 Mpeg1 macrophage expressed 1 Rattus norvegicus
64619 Zfp238 zinc finger protein 238 Rattus norvegicus
64681 Mvp major vault protein Rattus norvegicus
65144 Pdzk1 PDZ domain containing 1 Rattus norvegicus
65165 Tmed2 transmembrane p24 trafficking protein 2 Rattus norvegicus
66028 Arl6ip5 ADP-ribosylation factor like GTPase 6 interacting protein 5 Rattus norvegicus
678772 Casd1 CAS1 domain containing 1 Rattus norvegicus
679692 Lpgat1 lysophosphatidylglycerol acyltransferase 1 Rattus norvegicus
680782 Spcs3 signal peptidase complex subunit 3 Rattus norvegicus
686179 Ttc39c tetratricopeptide repeat domain 39C Rattus norvegicus
688405 Cdk2ap2 cyclin-dependent kinase 2 associated protein 2 Rattus norvegicus
688478 Commd2 COMM domain containing 2 Rattus norvegicus
79438 Igfals insulin-like growth factor binding protein, acid labile subunit Rattus norvegicus
81646 Creb1 cAMP responsive element binding protein 1 Rattus norvegicus
81748 Pls3 plastin 3 Rattus norvegicus
83501 Cdh2 cadherin 2 Rattus norvegicus
83534 Tpp1 tripeptidyl peptidase 1 Rattus norvegicus
83576 Sort1 sortilin 1 Rattus norvegicus
83586 Usf1 upstream transcription factor 1 Rattus norvegicus
84027 Gsk3b glycogen synthase kinase 3 beta Rattus norvegicus
84475 Cxcr3 C-X-C motif chemokine receptor 3 Rattus norvegicus
85332 Prkcdbp protein kinase C, delta binding protein Rattus norvegicus
94168 Spp2 secreted phosphoprotein 2 Rattus norvegicus
94269 Fez2 fasciculation and elongation protein zeta 2 Rattus norvegicus

Supplementary Table 7

Gene list of “M4vs8_cluster3”
This cluster was obtained by hierarchical clustering (dendrogram cutoff: 6) of DEGs between
WY-14643/M dose/4day and WY-14643/M dose/8day by student’s t-test (cutoff: p-value=0.01).
The cluster that contained 162 probes was defined as “M4vs8_cluster3”.

100125372 Ces1f carboxylesterase 1F Rattus norvegicus
100360302 Sec16a SEC16 homolog A, endoplasmic reticulum export factor Rattus norvegicus
100911750 LOC100911750 N-acetyllactosaminide beta-1,3-N-acetylglucosaminyltransferase-like Rattus norvegicus
114517 Itga6 integrin subunit alpha 6 Rattus norvegicus
114558 Becn1 beclin 1 Rattus norvegicus
116504 Mprip myosin phosphatase Rho interacting protein Rattus norvegicus
116641 Lgals8 galectin 8 Rattus norvegicus
116655 Hnrnpm heterogeneous nuclear ribonucleoprotein M Rattus norvegicus
116686 Gsr glutathione-disulfide reductase Rattus norvegicus
140934 Ikbkap inhibitor of kappa light polypeptide gene enhancer in B-cells, kinase complex-associated protein Rattus norvegicus
171337 Rap1b RAP1B, member of RAS oncogene family Rattus norvegicus
24242 Calm1 calmodulin 1 Rattus norvegicus
24244 Calm3 calmodulin 3 Rattus norvegicus
24385 Gck glucokinase Rattus norvegicus
24400 Gnb1 G protein subunit beta 1 Rattus norvegicus
246282 Zfp91 zinc finger protein 91 Rattus norvegicus
24645 Pgm1 phosphoglucomutase 1 Rattus norvegicus
24851 Tpm1 tropomyosin 1, alpha Rattus norvegicus
25094 Mitf melanogenesis associated transcription factor Rattus norvegicus
25283 Gclc glutamate-cysteine ligase, catalytic subunit Rattus norvegicus
25578 Ywhaz tyrosine 3-monooxygenase/tryptophan 5-monooxygenase activation protein, zeta Rattus norvegicus
266764 Tbkbp1 TBK1 binding protein 1 Rattus norvegicus
282587 Cttnbp2 cortactin binding protein 2 Rattus norvegicus
282635 Mbnl1 muscleblind-like splicing regulator 1 Rattus norvegicus
287170 Snrnp25 small nuclear ribonucleoprotein U11/U12 subunit 25 Rattus norvegicus
287719 Ifi35 interferon-induced protein 35 Rattus norvegicus
287986 Eif4g1 eukaryotic translation initiation factor 4 gamma, 1 Rattus norvegicus
288692 Wsb2 WD repeat and SOCS box-containing 2 Rattus norvegicus
288921 Fbxw9 F-box and WD repeat domain containing 9 Rattus norvegicus

289424	Xpr1	xenotropic and polytropic retrovirus receptor 1	Rattus norvegicus
289820	Actr2	ARP2 actin related protein 2 homolog	Rattus norvegicus
290577	Wapl	WAPL cohesin release factor	Rattus norvegicus
291320	Fam188a	family with sequence similarity 188, member A	Rattus norvegicus
292022	Ddx19a	DEAD-box helicase 19A	Rattus norvegicus
292155	Hs2st1	heparan sulfate 2-O-sulfotransferase 1	Rattus norvegicus
293186	Lyve1	lymphatic vessel endothelial hyaluronan receptor 1	Rattus norvegicus
293489	Slx1b	SLX1 structure-specific endonuclease subunit homolog B (S. cerevisiae)	Rattus norvegicus
29355	Pkn1	protein kinase N1	Rattus norvegicus
293667	B4gat1	beta-1,4-glucuronyltransferase 1	Rattus norvegicus
293939	Erlin1	ER lipid raft associated 1	Rattus norvegicus
293967	Smc5	structural maintenance of chromosomes 5	Rattus norvegicus
294004	Dpcd	deleted in primary ciliary dyskinesia	Rattus norvegicus
29534	Pex2	peroxisomal biogenesis factor 2	Rattus norvegicus
295457	Cisd2	CDGSH iron sulfur domain 2	Rattus norvegicus
29562	Prps1	phosphoribosyl pyrophosphate synthetase 1	Rattus norvegicus
29606	Pcsk7	proprotein convertase subtilisin/kexin type 7	Rattus norvegicus
296634	Nup214	nucleoporin 214	Rattus norvegicus
297387	Mob1a	MOB kinase activator 1A	Rattus norvegicus
298084	Zbtb5	zinc finger and BTB domain containing 5	Rattus norvegicus
298712	Gpalpp1	GPALPP motifs containing 1	Rattus norvegicus
299811	Cpsf6	cleavage and polyadenylation specific factor 6	Rattus norvegicus
300284	Alg13	ALG13, UDP-N-acetylglucosaminyltransferase subunit	Rattus norvegicus
300837	Leo1	LEO1 homolog, Paf1/RNA polymerase II complex component	Rattus norvegicus
302863	Slc9a6	solute carrier family 9 member A6	Rattus norvegicus
302969	Srrm2	serine/arginine repetitive matrix 2	Rattus norvegicus
304294	Rnf216	ring finger protein 216	Rattus norvegicus
304786	Elk4	ELK4, ETS transcription factor	Rattus norvegicus
305264	Ugt2b10	UDP glucuronosyltransferase 2 family, polypeptide B10	Rattus norvegicus
305889	Thtpa	thiamine triphosphatase	Rattus norvegicus
306254	Pbrm1	polybromo 1	Rattus norvegicus
306487	Mtus1	microtubule associated tumor suppressor 1	Rattus norvegicus
306871	Dsp	desmoplakin	Rattus norvegicus
307595	RGD1562794	similar to establishment of cohesion 1 homolog 1	Rattus norvegicus
309262	Nsdhl	NAD(P) dependent steroid dehydrogenase-like	Rattus norvegicus

309639 Anks1a ankyrin repeat and sterile alpha motif domain containing 1A Rattus norvegicus
310538 Fnip2 folliculin interacting protein 2 Rattus norvegicus
311118 Tlk1 tousled-like kinase 1 Rattus norvegicus
311483 Rrbp1 ribosome binding protein 1 Rattus norvegicus
311743 Zak sterile alpha motif and leucine zipper containing kinase AZK Rattus norvegicus
311911 Rabgap1 RAB GTPase activating protein 1 Rattus norvegicus
313139 Slc35a1 solute carrier family 35 member A1 Rattus norvegicus
314273 Dcaf5 DDB1 and CUL4 associated factor 5 Rattus norvegicus
316313 Dst dystonin Rattus norvegicus
316531 Tuba4a tubulin, alpha 4A Rattus norvegicus
360531 Sap30l SAP30-like Rattus norvegicus
360563 Ncbp3 nuclear cap binding subunit 3 Rattus norvegicus
361842 Sar1a secretion associated, Ras related GTPase 1A Rattus norvegicus
362029 Agl amylo-alpha-1, 6-glucosidase, 4-alpha-glucanotransferase Rattus norvegicus
362359 Mpp6 membrane palmitoylated protein 6 Rattus norvegicus
362461 C2cd5 C2 calcium-dependent domain containing 5 Rattus norvegicus
362514 Tstd2 thiosulfate sulfurtransferase like domain containing 2 Rattus norvegicus
362883 Slc35e3 solute carrier family 35, member E3 Rattus norvegicus
363022 Zfp846 zinc finger protein 846 Rattus norvegicus
363029 Swsap1 SWIM-type zinc finger 7 associated protein 1 Rattus norvegicus
363077 Dis3l DIS3-like exosome 3'-5' exoribonuclease Rattus norvegicus
363118 Nmnat3 nicotinamide nucleotide adenylyltransferase 3 Rattus norvegicus
363445 Usp9x ubiquitin specific peptidase 9, X-linked Rattus norvegicus
365410 Osbp oxysterol binding protein Rattus norvegicus
366311 Clvs1 clavesin 1 Rattus norvegicus
497961 Nlk nemo like kinase Rattus norvegicus
498411 Hus1 HUS1 checkpoint clamp component Rattus norvegicus
499443 Lims1 LIM zinc finger domain containing 1 Rattus norvegicus
501232 Cesl1 carboxylesterase-like 1 Rattus norvegicus
50663 Calm2 calmodulin 2 Rattus norvegicus
58840 Mapk6 mitogen-activated protein kinase 6 Rattus norvegicus
58954 Klf6 Kruppel-like factor 6 Rattus norvegicus
59076 Grk6 G protein-coupled receptor kinase 6 Rattus norvegicus
64314 Casp2 caspase 2 Rattus norvegicus
64391 Dapk3 death-associated protein kinase 3 Rattus norvegicus
65134 Stx5 syntaxin 5 Rattus norvegicus
679127 Rrp12 ribosomal RNA processing 12 homolog Rattus norvegicus
680014 Esco1 establishment of sister chromatid cohesion N-

acetyltransferase 1 Rattus norvegicus
680562 LOC680562 similar to Mob4B protein Rattus norvegicus
681740 Jarid2 jumonji and AT-rich interaction domain containing 2 Rattus norvegicus
685029 Anapc13 anaphase promoting complex subunit 13 Rattus norvegicus
686295 Wdr82 WD repeat domain 82 Rattus norvegicus
689116 Ncbp2 nuclear cap binding protein subunit 2 Rattus norvegicus
689134 Sec61g Sec61 translocon gamma subunit Rattus norvegicus
689755 Ccdc167 coiled-coil domain containing 167 Rattus norvegicus
689997 Fam91a1 family with sequence similarity 91, member A1 Rattus norvegicus
690693 Ddx19b DEAD-box helicase 19B Rattus norvegicus
690779 LOC690779 similar to Mob4B protein Rattus norvegicus
81727 Mvk mevalonate kinase Rattus norvegicus
83469 Lrp4 LDL receptor related protein 4 Rattus norvegicus
83536 Mkln1 muskelin 1 Rattus norvegicus
83584 Casp6 caspase 6 Rattus norvegicus
83720 Fat1 FAT atypical cadherin 1 Rattus norvegicus
84599 Tmed10 transmembrane p24 trafficking protein 10 Rattus norvegicus
89842 Mbtps1 membrane-bound transcription factor peptidase, site 1 Rattus norvegicus
