# Supplementary material for: Interactive Toxicogenomics: Gene set discovery, clustering and analysis in Toxygates
Source: Sci Rep. 2017 May 3;7:1390. doi: 10.1038/s41598-017-01500-1 (PMC5431224; doi:10.1038/s41598-017-01500-1)
Supplement: Supplementary file 2 — Supplementary Methods [file 41598_2017_1500_MOESM2_ESM.pdf]

Interactive Toxicogenomics: Gene Set Discovery, Clustering and Analysis in Toxygates

Nyström-Persson, J., Natsume-Kitatani, Y., Igarashi, Y., Satoh, D., and Mizuguchi, K.

Supplementary Methods

This supplementary document includes the details of the analytical flow in our case study in which the new analytical functions in Toxygates were utilized.
For the figures of heatmap and the interpretation of the obtained results, please refer to the main manuscript. For the general tutorial for Toxygates, please refer to the user guide (http://toxygates.nibiohn.go.jp/toxygates/toxygatesManual.pdf)

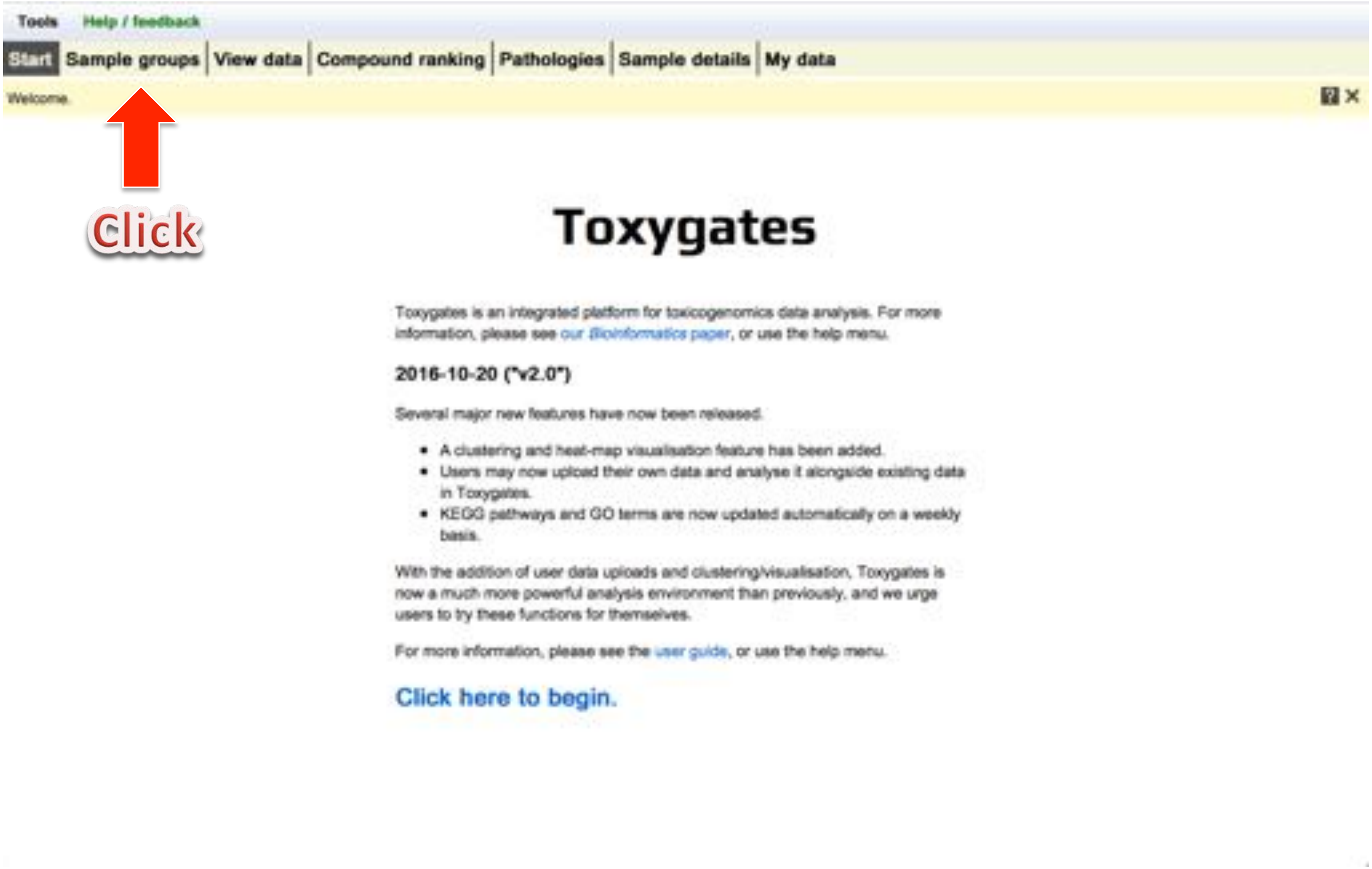

Tools
Help / feedback
Start
Sample groups
View data
Compound ranking
Pathologies
Sample details
My data
Welcome.
Click
Toxygates
Toxygates is an integrated platform for toxicogenomics data analysis. For more information, please see our Bioinformatics paper, or use the help menu.
2016-10-20 ("v2.0")
Several major new features have now been released.
A clustering and heat-map visualisation feature has been added.
Users may now upload their own data and analyse it alongside existing data in Toxygates.
KEGG pathways and GO terms are now updated automatically on a weekly basis.
With the addition of user data uploads and clustering/visualisation, Toxygates is now a much more powerful analysis environment than previously, and we urge users to try these functions for themselves.
For more information, please see the user guide, or use the help menu.
Click here to begin.

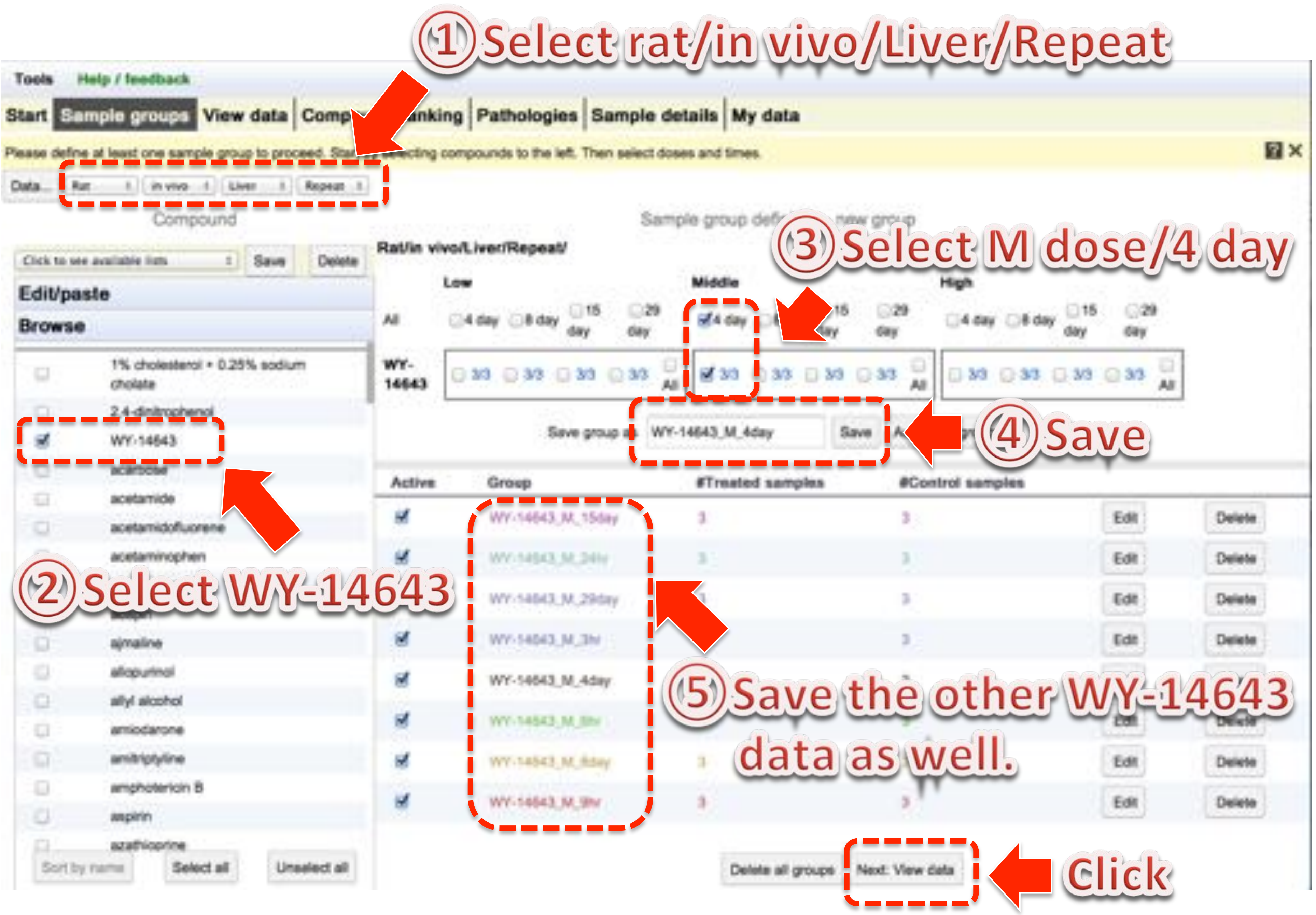

①Select rat/in vivo/Liver/Repeat
Tools
Help / feedback
Start
Sample groups
View data
Pathologies
Sample details
My data
Rat
in vivo
Liver
Repeat
Compound
Save
Delete
Edit/paste
Browse
Rat/in vivo/Liver/Repeat/
Low
Middle
High
③Select M dose/4 day
WY-14643
WY-14643_M_4day
Save
④Save
Active
Group
#Treated samples
#Control samples
Edit
Delete
②Select WY-14643
acetamide
acetaminophen
ajmaline
allopurinol
allyl alcohol
amiodarone
amitriptyline
amphotericin B
aspirin
azathioprine
⑤Save the other WY-14643 data as well.
Select all
Unselect all
Delete all groups
Next: View data
Click

Observed pathologies as starting point

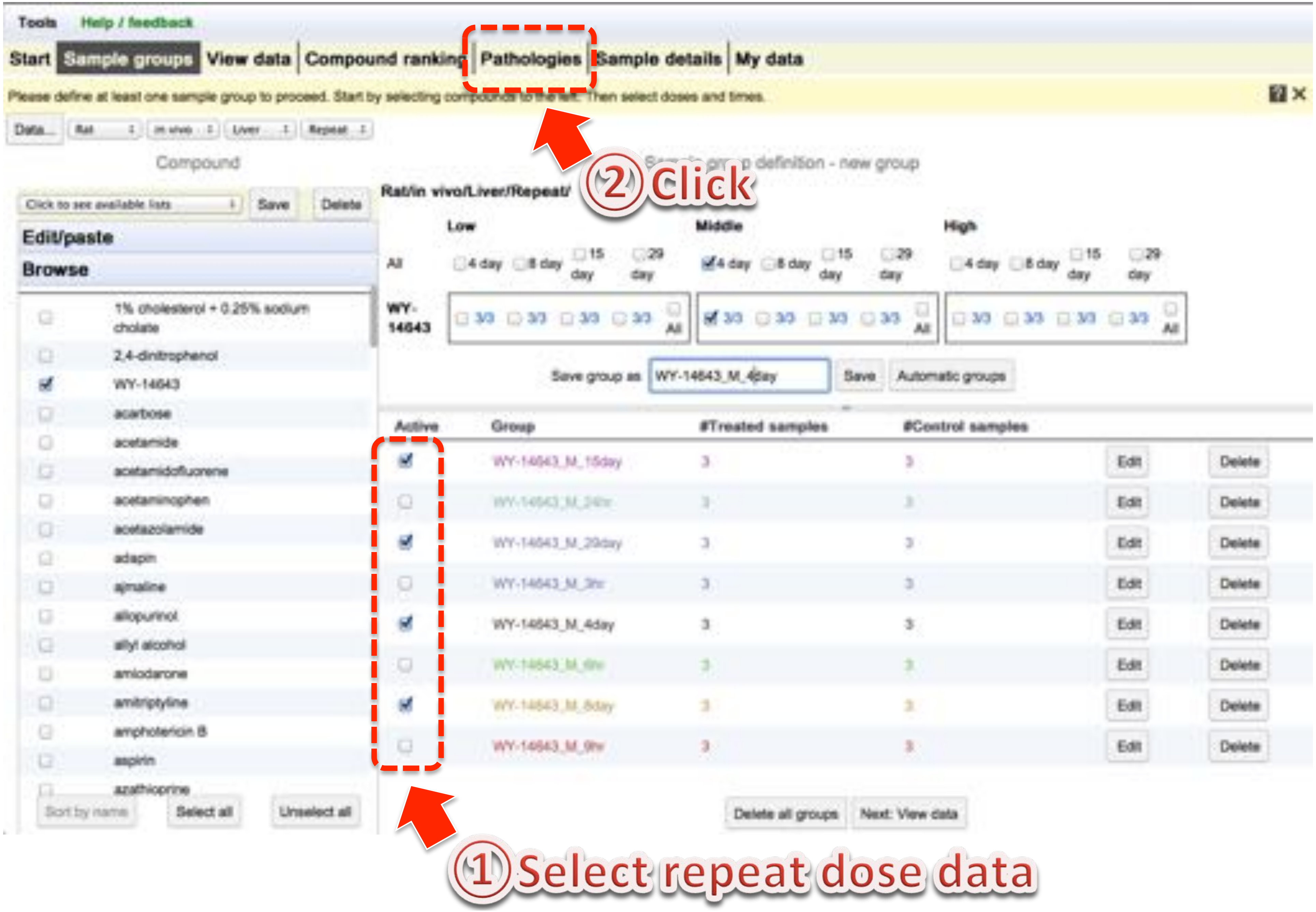

Tools Help / feedback

Start | Sample groups | View data | Compound ranking | Pathologies | Sample details | My data

This is the list of pathologies in the sample groups you have defined. Click on an icon to see detailed sample information.

WY-14643_M_15day :WY-14643/Middle/15 day | WY-14643_M_29day :WY-14643/Middle/29 day | WY-14643_M_4day :WY-14643/Middle/4 day | WY-14643_M_8day :WY-14643/Middle/8 day

Pathology terms reference

Group	Sample		Finding	Topography	Grade	Spontaneous	Digital viewer
WY-14643_M_15day	WY-14643/Middle/15 day/undefined		Degeneration, granular, eosinophilic	Hepatocyte	Severe (+++)	false	Viewer
WY-14643_M_15day	WY-14643/Middle/15 day/undefined		Degeneration, granular, eosinophilic	Hepatocyte	Severe (+++)	false	Viewer
WY-14643_M_15day	WY-14643/Middle/15 day/undefined		Degeneration, granular, eosinophilic	Hepatocyte	Severe (+++)	false	Viewer
WY-14643_M_15day	WY-14643/Middle/15 day/undefined		Necrosis	Hepatocyte	Minimal (±)	false	Viewer
WY-14643_M_15day	WY-14643/Middle/15 day/undefined		Hypertrophy	Bile duct, interlobular	Slight (+)	false	Viewer
WY-14643_M_8day	WY-14643/Middle/8 day/undefined		Single cell necrosis	Hepatocyte	Minimal (±)	false	Viewer
WY-14643_M_8day	WY-14643/Middle/8 day/undefined		Degeneration, granular, eosinophilic	Hepatocyte	Severe (+++)	false	Viewer
WY-14643_M_8day	WY-14643/Middle/8 day/undefined		Degeneration, granular, eosinophilic	Hepatocyte	Severe (+++)	false	Viewer
WY-14643_M_8day	WY-14643/Middle/8 day/undefined		Degeneration, granular, eosinophilic	Hepatocyte	Severe (+++)	false	Viewer
WY-14643_M_8day	WY-14643/Middle/8 day/undefined		Hypertrophy	Bile duct, interlobular	Slight (+)	false	Viewer
WY-14643_M_15day	WY-14643/Middle/15 day/undefined		Hypertrophy	Bile duct, interlobular	Minimal (±)	false	Viewer
WY-14643_M_15day	WY-14643/Middle/15 day/undefined		Hypertrophy	Bile duct, interlobular	Minimal (±)	false	Viewer
WY-14643_M_29day	WY-14643/Middle/29 day/undefined		Necrosis	Hepatocyte	Slight (+)	false	Viewer
WY-14643_M_4day	WY-14643/Middle/4 day/undefined		Increased mitosis	Hepatocyte	Slight (+)	false	Viewer
WY-14643_M_4day	WY-14643/Middle/4 day/undefined		Increased mitosis	Hepatocyte	Slight (+)	false	Viewer
WY-14643_M_29day	WY-14643/Middle/29 day/undefined		Hypertrophy	Bile duct, interlobular	Minimal (±)	false	Viewer
WY-14643_M_29day	WY-14643/Middle/29 day/undefined		Degeneration, granular, eosinophilic	Hepatocyte	Severe (+++)	false	Viewer
WY-14643_M_29day	WY-14643/Middle/29 day/undefined		Hypertrophy	Bile duct, interlobular	Slight (+)	false	Viewer
WY-14643_M_29day	WY-14643/Middle/29 day/undefined		Degeneration, granular, eosinophilic	Hepatocyte	Severe (+++)	false	Viewer
WY-14643_M_29day	WY-14643/Middle/29 day/undefined		Hypertrophy	Bile duct, interlobular	Slight (+)	false	Viewer
WY-14643_M_29day	WY-14643/Middle/29 day/undefined		Degeneration, granular, eosinophilic	Hepatocyte	Severe (+++)	false	Viewer
[illegible]	[illegible]		[illegible]	[illegible]	[illegible]	[illegible]	[illegible]

Supplementary Table 1: summary of this table

Cluster analysis

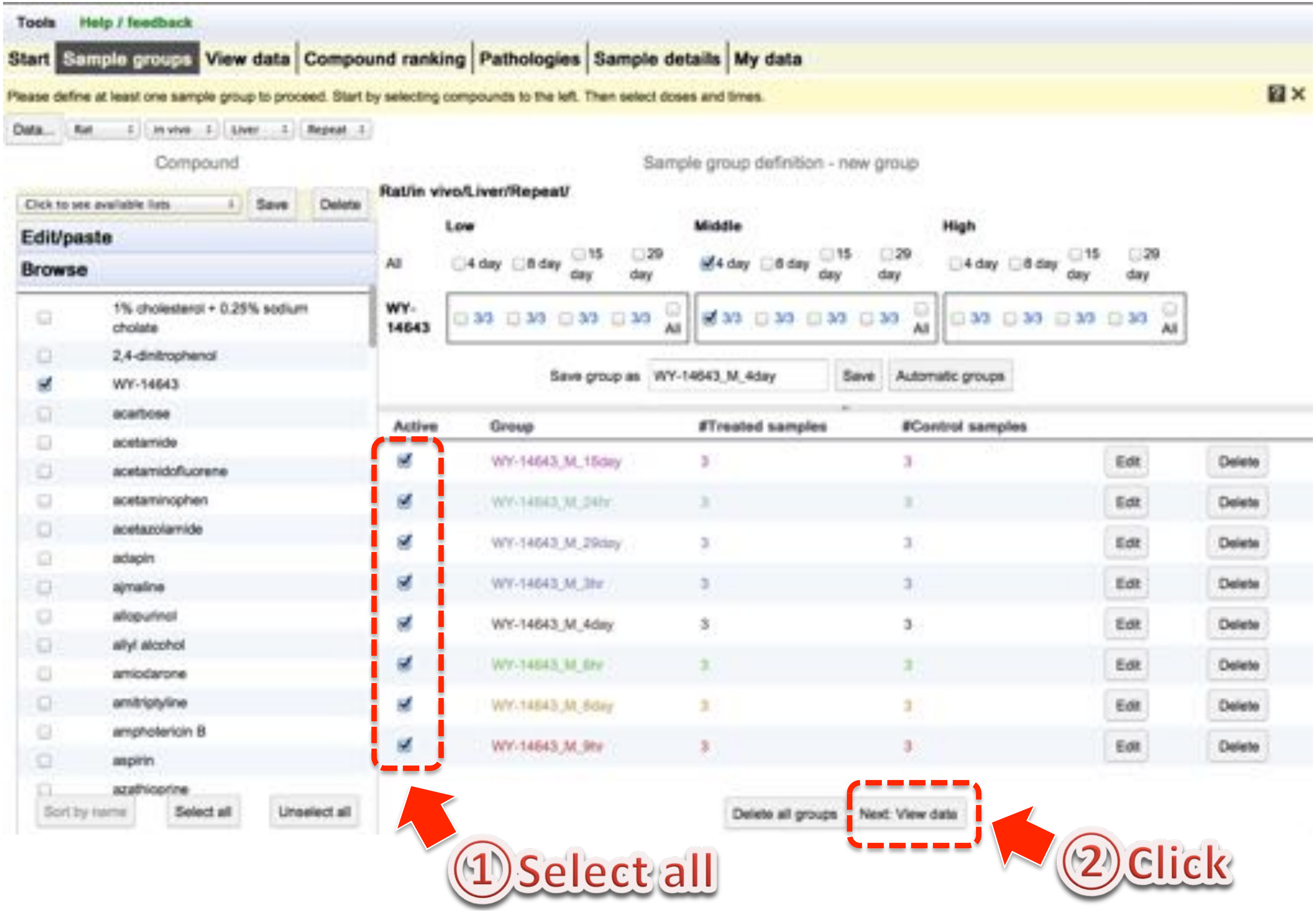

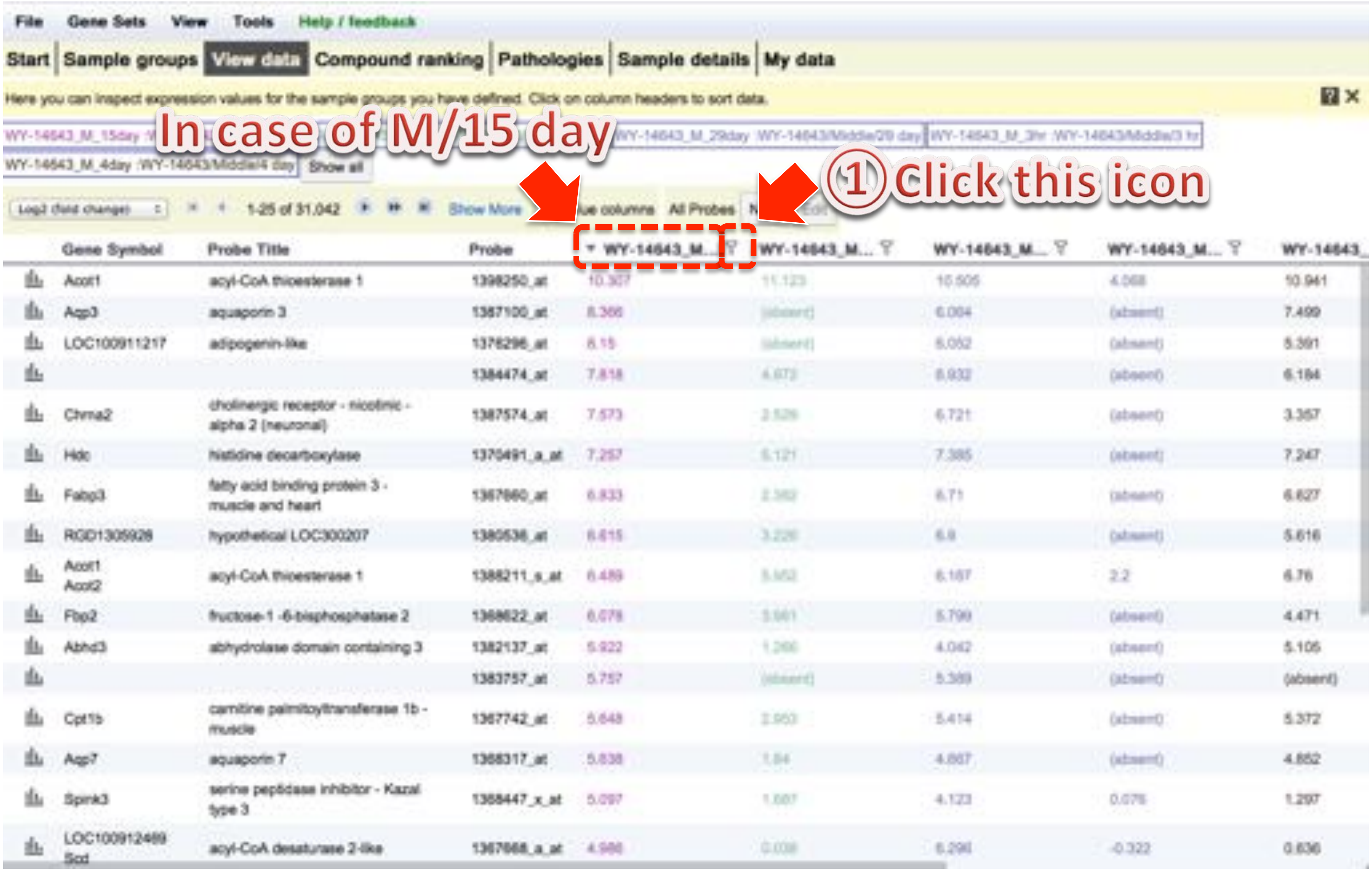
File
Gene Sets
View
Tools
Help / feedback
Start
Sample groups
View data
Compound ranking
Pathologies
Sample details
My data
Here you can inspect expression values for the sample groups you have defined. Click on column headers to sort data.
In case of M/15 day
①Click this icon
Gene Symbol
Probe Title
Probe

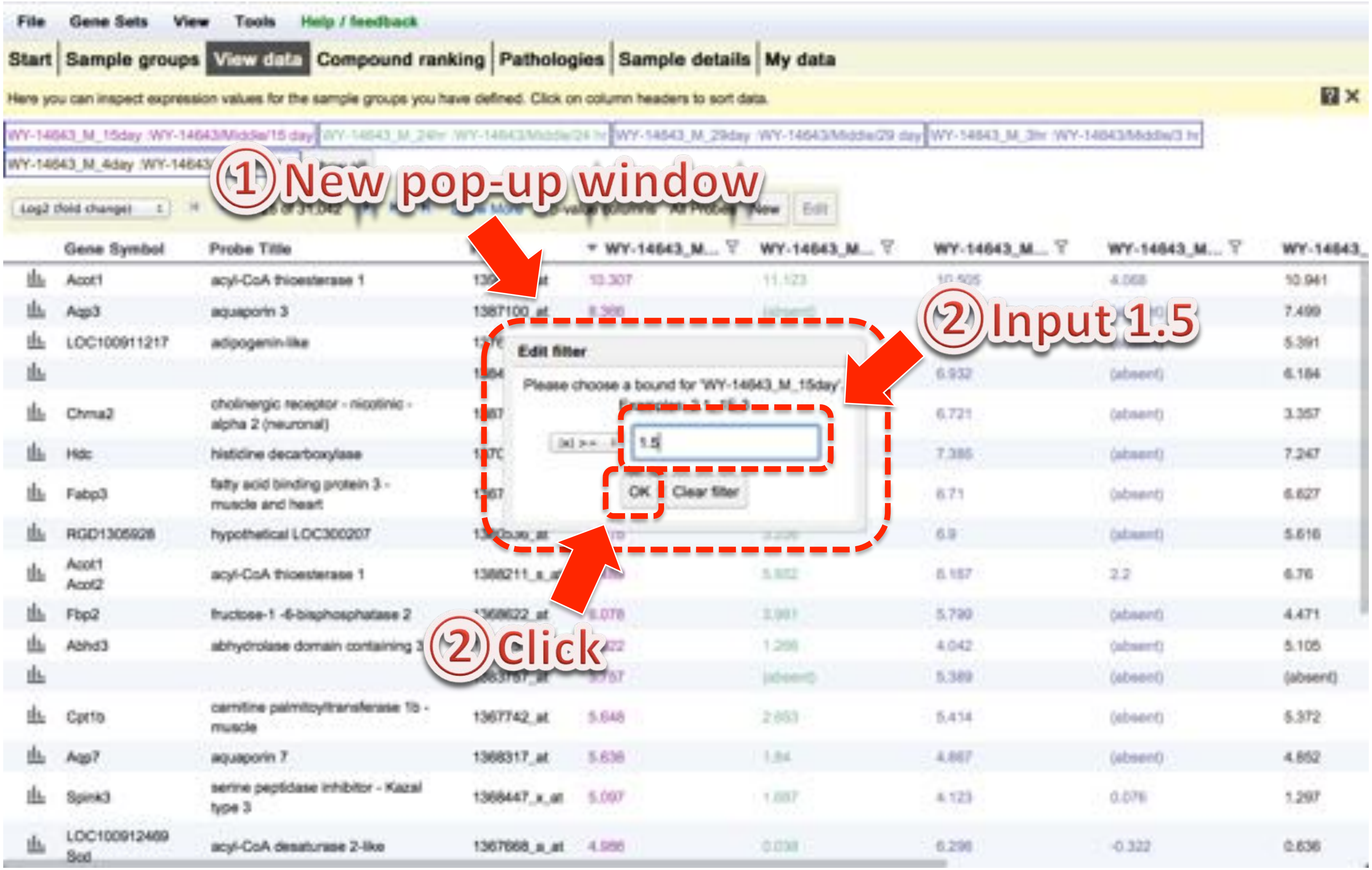
File
Gene Sets
View
Tools
Help / feedback
Start
Sample groups
View data
Compound ranking
Pathologies
Sample details
My data
Here you can inspect expression values for the sample groups you have defined. Click on column headers to sort data.
①New pop-up window
②Input 1.5
Edit filter
OK
Clear filter
②Click
Gene Symbol
Probe Title

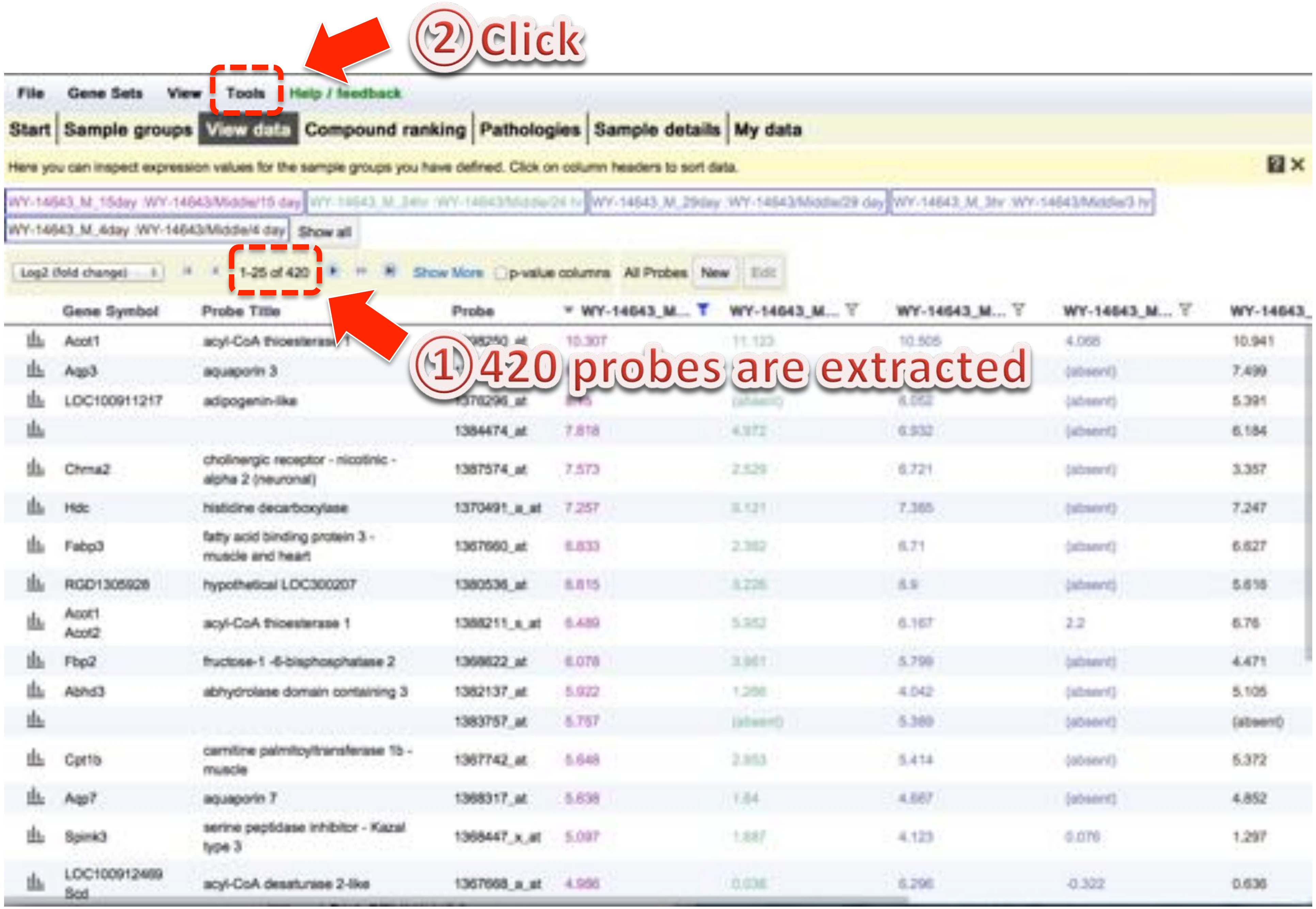
②Click
File
Gene Sets
View
Tools
Help / feedback
Start
Sample groups
View data
Compound ranking
Pathologies
Sample details
My data
Here you can inspect expression values for the sample groups you have defined. Click on column headers to sort data.
Show all
Log2 (fold change)
1-25 of 420
Show More
p-value columns
All Probes
New
Edit
Gene Symbol
Probe Title
Probe
①420 probes are extracted

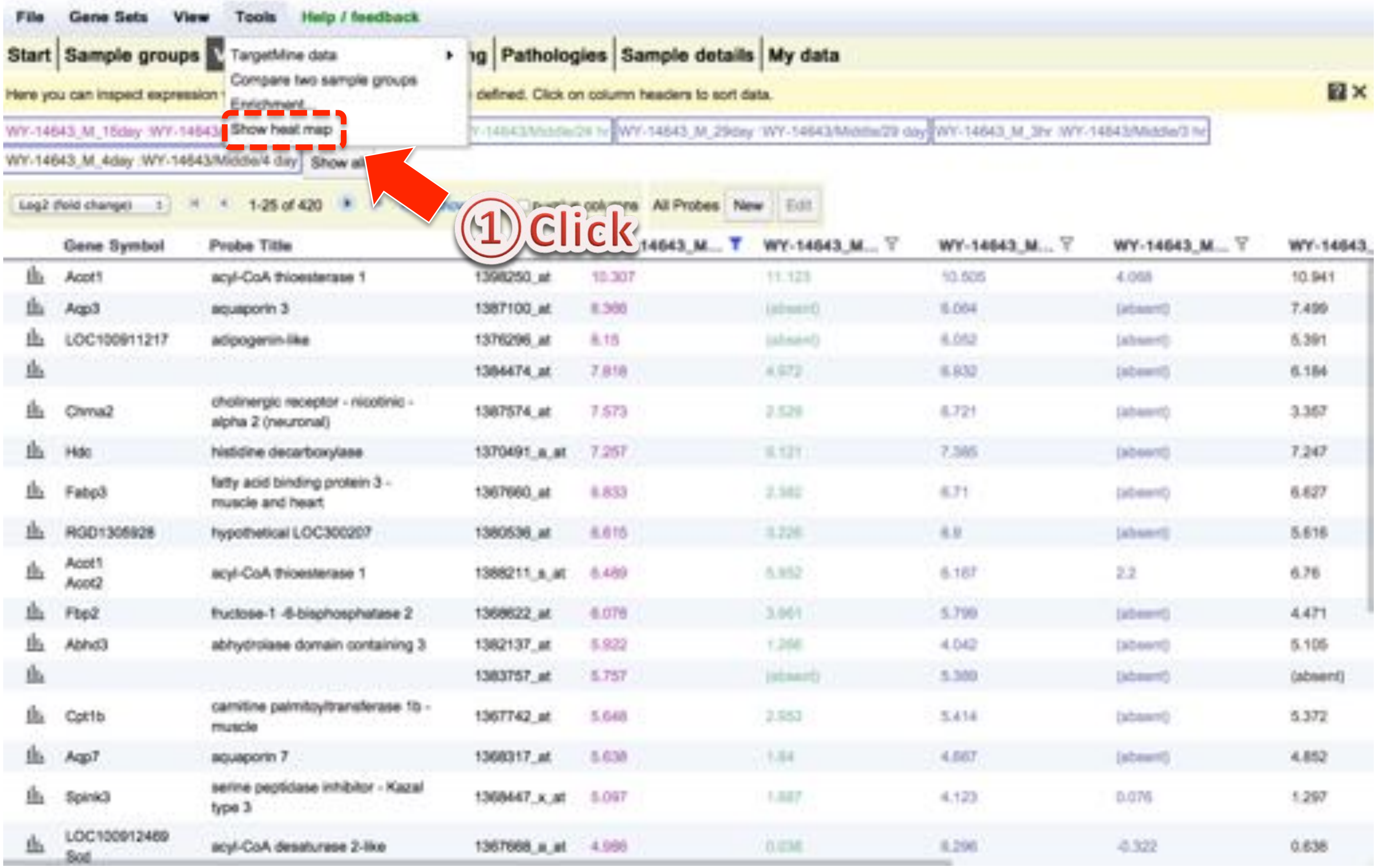
File
Gene Sets
View
Tools
Help / feedback
Start
Sample groups
Pathologies
Sample details
My data
TargetMine data
Compare two sample groups
Enrichment
Show heat map
Here you can inspect expression
defined. Click on column headers to sort data.
Log2 (fold change)
1-25 of 420
All Probes
New
Edit
①Click
Gene Symbol
Probe Title

①New pop-up window appears

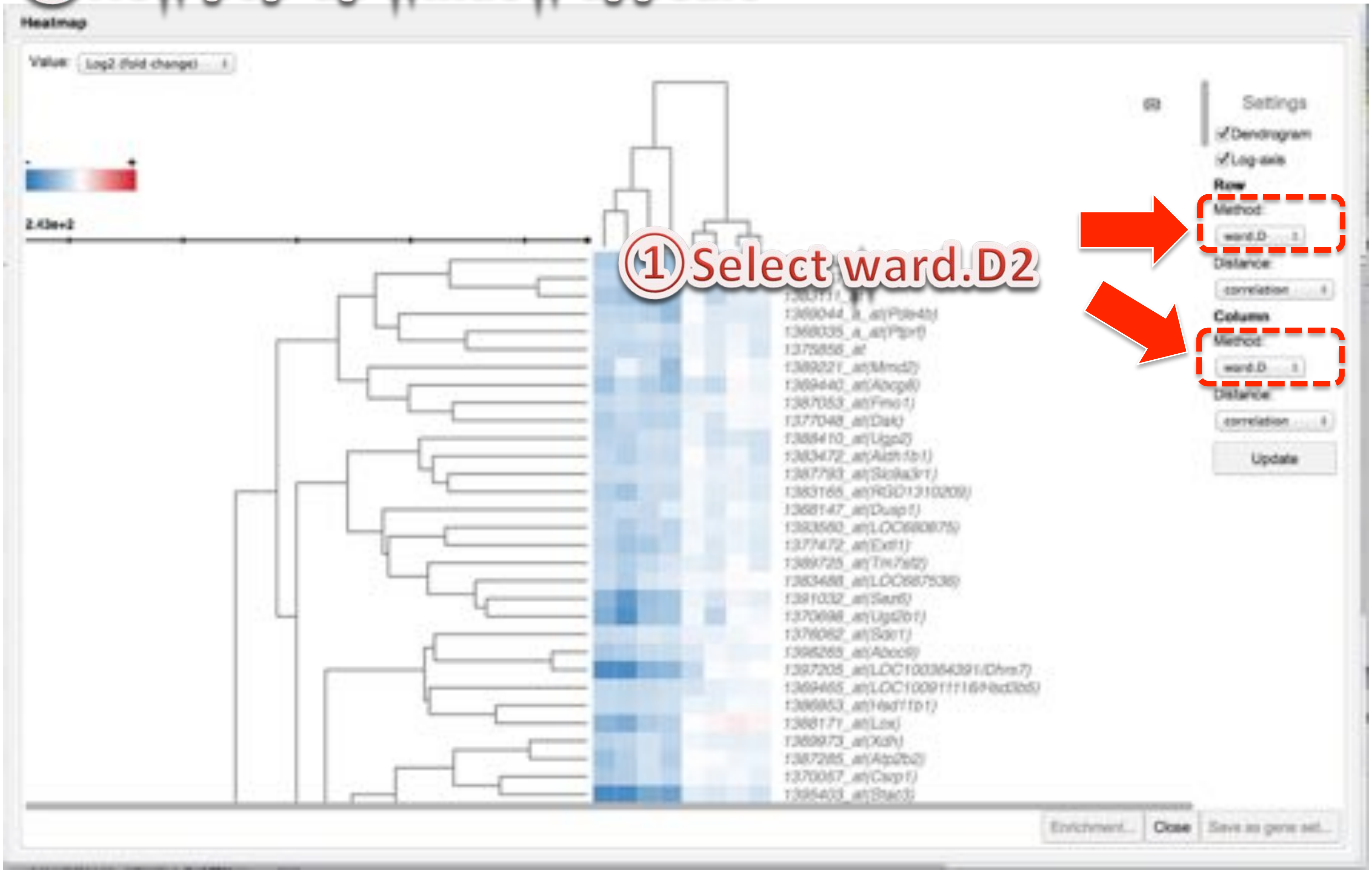

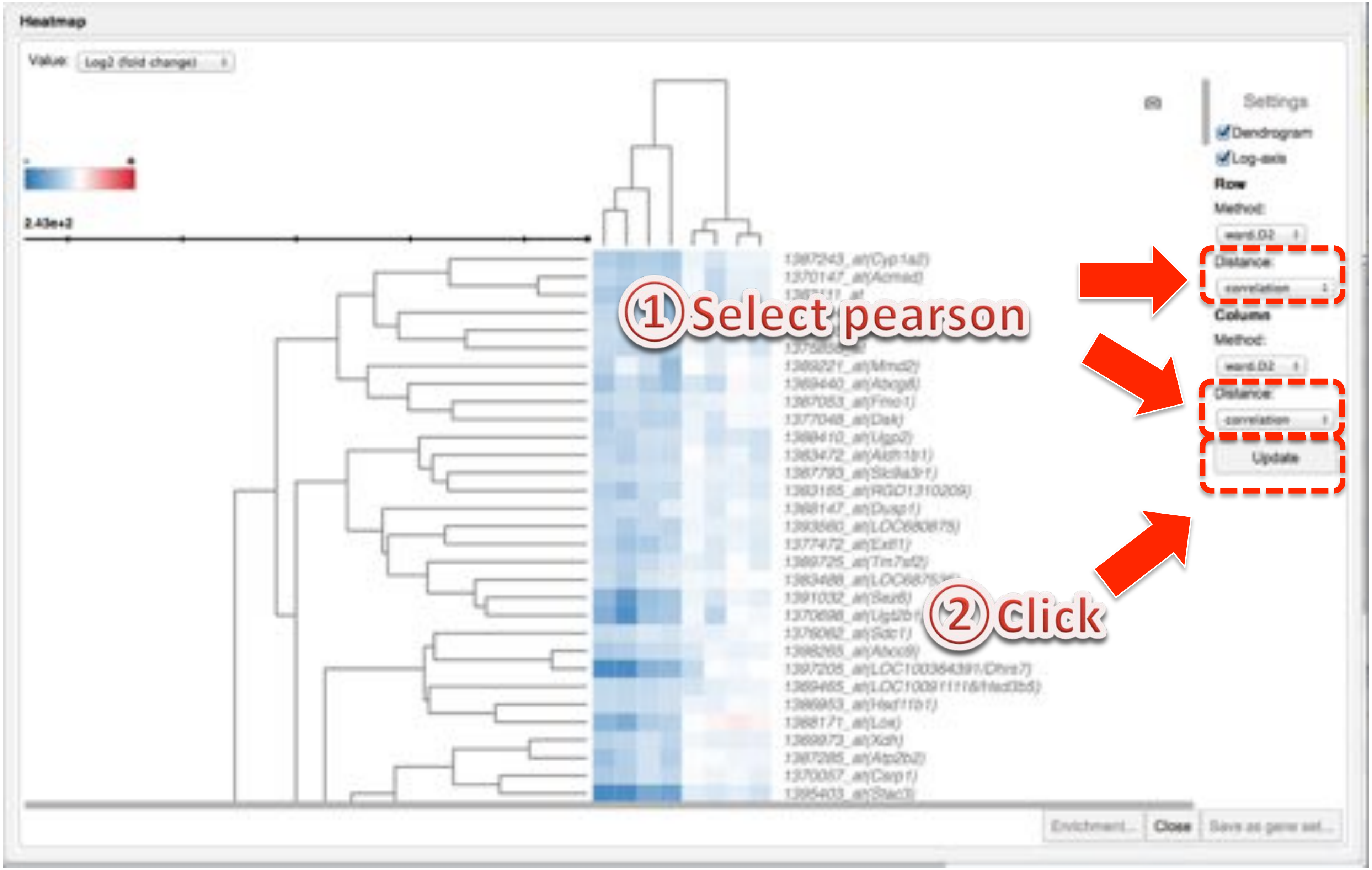
Heatmap
Value:
Log2 (fold change)
2.43e+2
Settings
Dendrogram
Log-axis
Row
Method:
ward.D2
Distance:
correlation
Column
Method:
ward.D2
Distance:
correlation
Update
①Select pearson
②Click
Enrichment...
Close
Save as gene set...

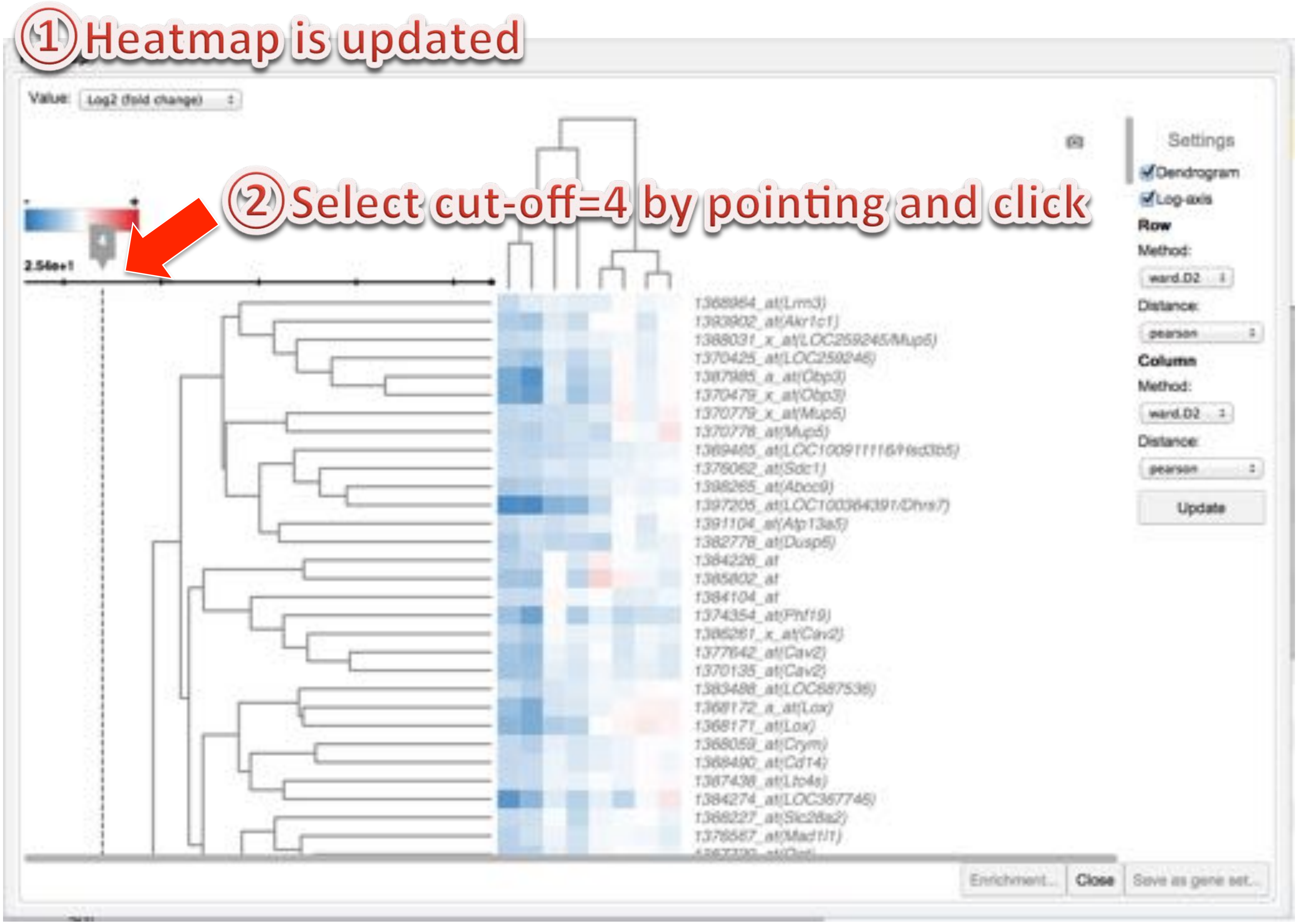
①Heatmap is updated
②Select cut-off=4 by pointing and click
Value:
Log2 (fold change)
Settings
Dendrogram
Log-axis
Row
Method:
ward.D2
Distance:
pearson
Column
Method:
ward.D2
Distance:
pearson
Update
Enrichment...
Close
Save as gene set...

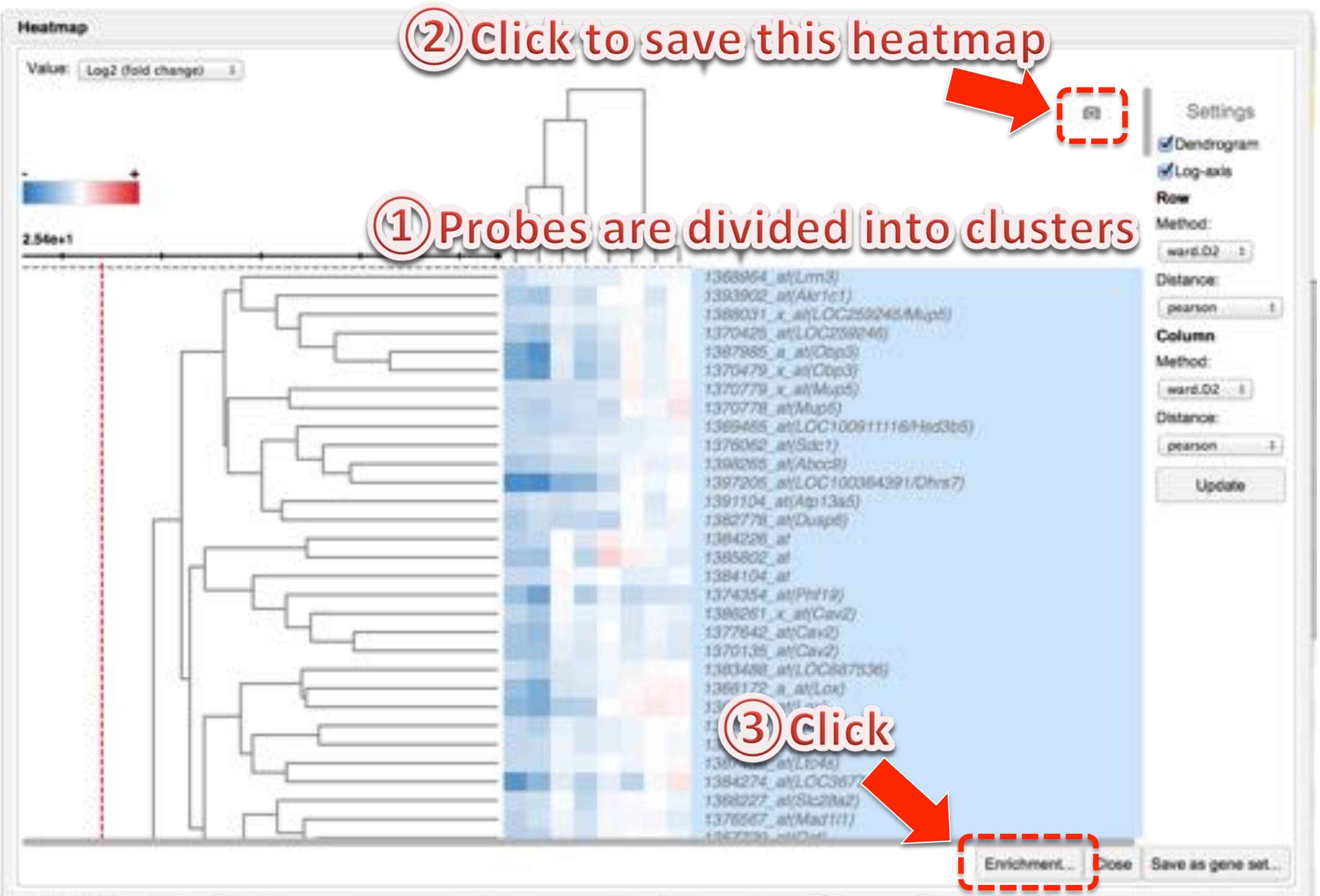

Supplementary Figure 3: image file of this heatmap

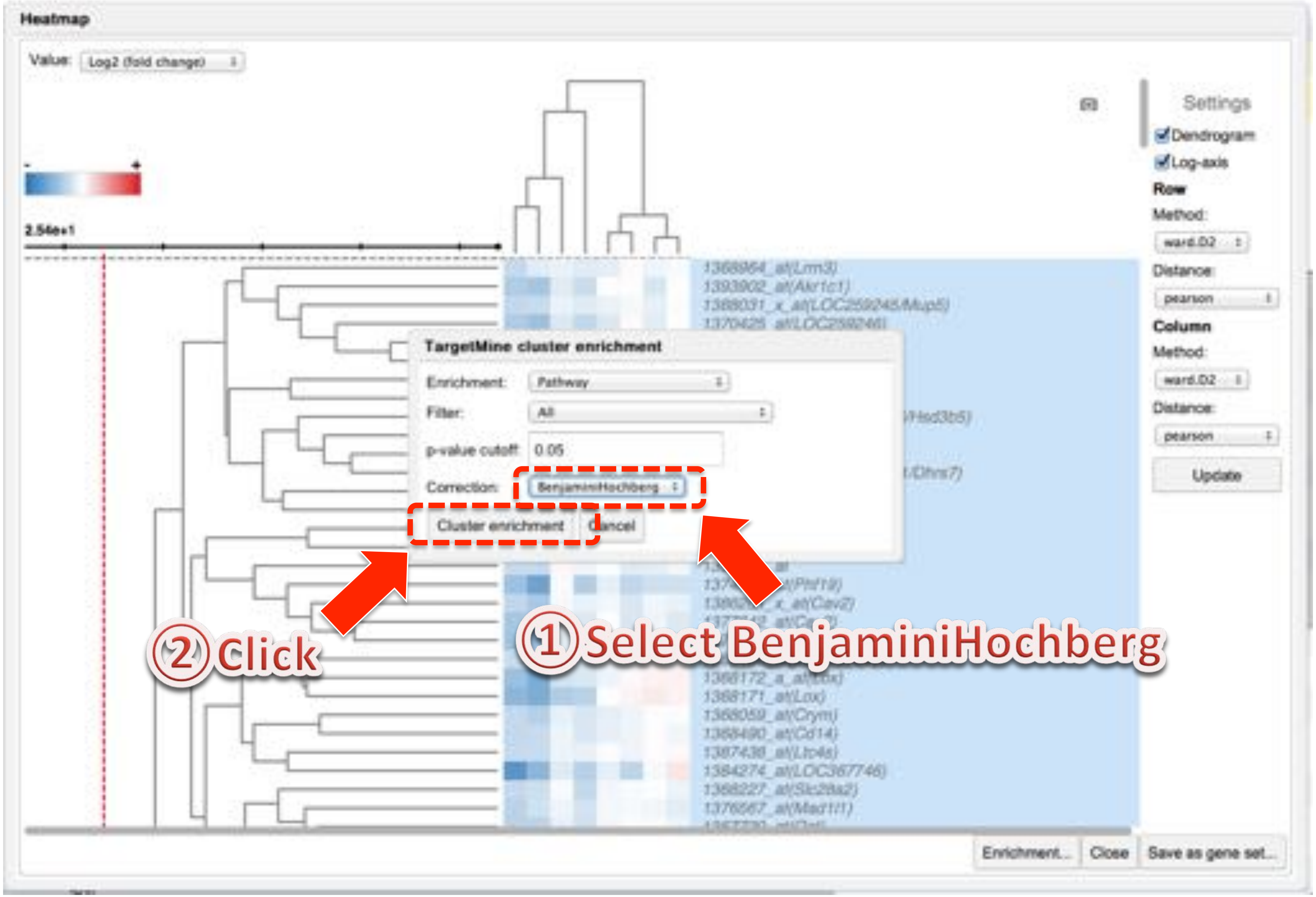
Heatmap
Value: Log2 (fold change)
Settings
Dendrogram
Log-axis
Row
Method:
ward.D2
Distance:
pearson
Column
Method:
ward.D2
Distance:
pearson
Update
TargetMine cluster enrichment
Enrichment: Pathway
Filter: All
p-value cutoff: 0.05
Correction: BenjaminiHochberg
Cluster enrichment
Cancel
Enrichment...
Close
Save as gene set...
②Click
①Select BenjaminiHochberg

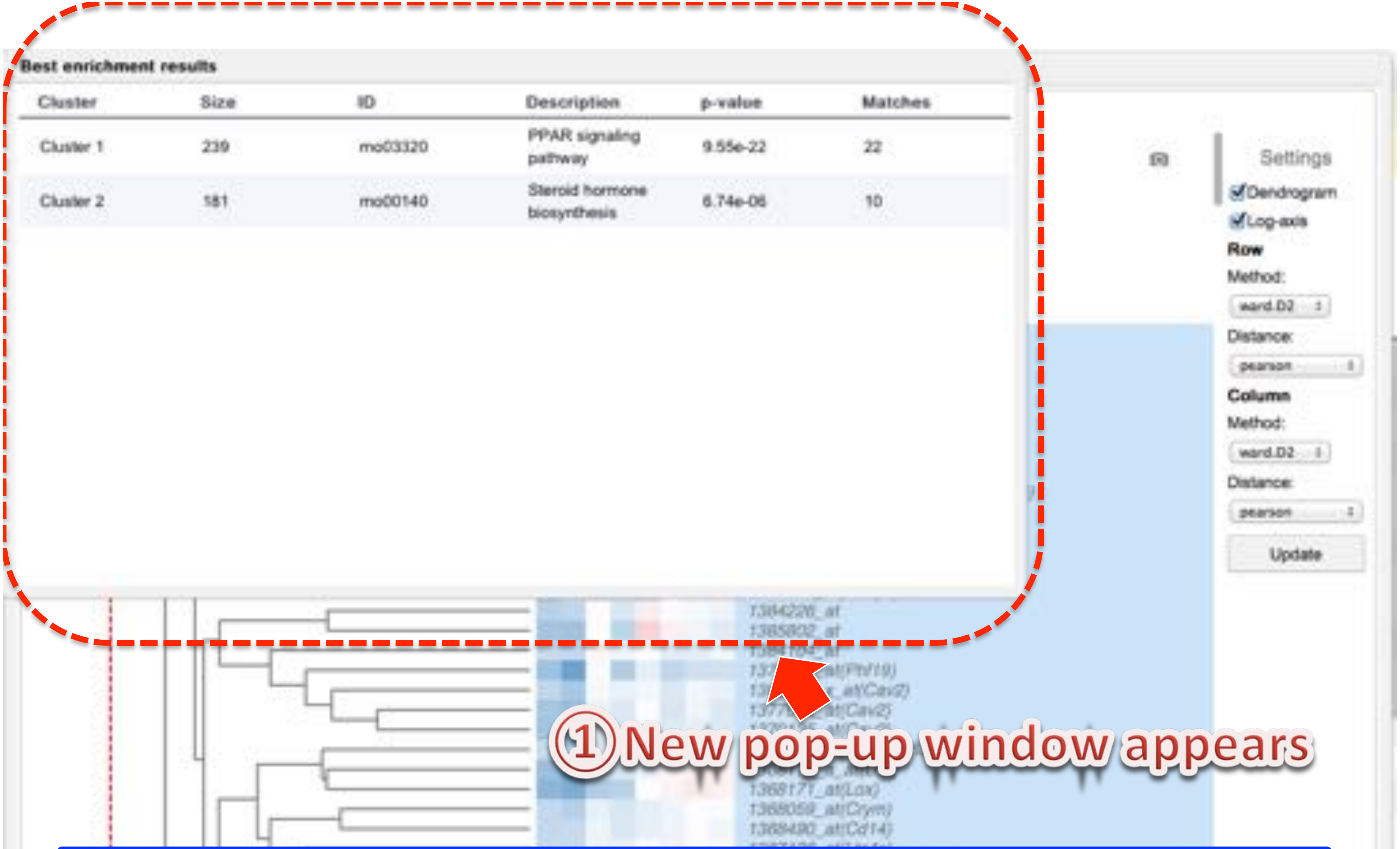

Supplementary Figure 1: Select M/4 day in “View data” page (slide 7)
Supplementary Figure 2: Select M/8 day in “View data” page (slide 7)
Supplementary Figure 4: Select M/29 day in “View data” page (slide 7)
and follow the same procedure to create the image files

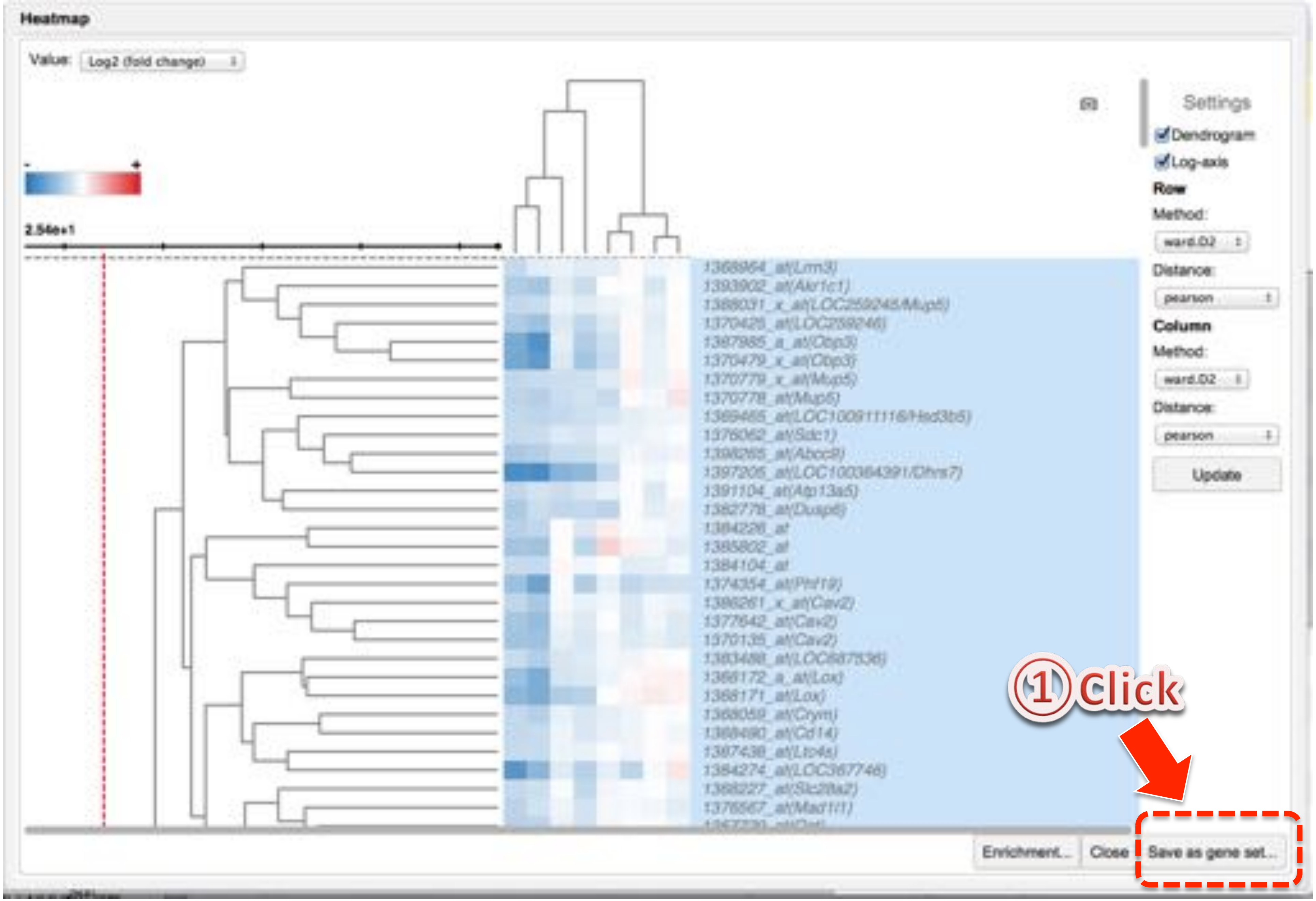
Heatmap
Value: Log2 (fold change)
2.54e+1
Settings
Dendrogram
Log-axis
Row
Method:
ward.D2
Distance:
pearson
Column
Method:
ward.D2
Distance:
pearson
Update
①Click
Enrichment...
Close
Save as gene set...

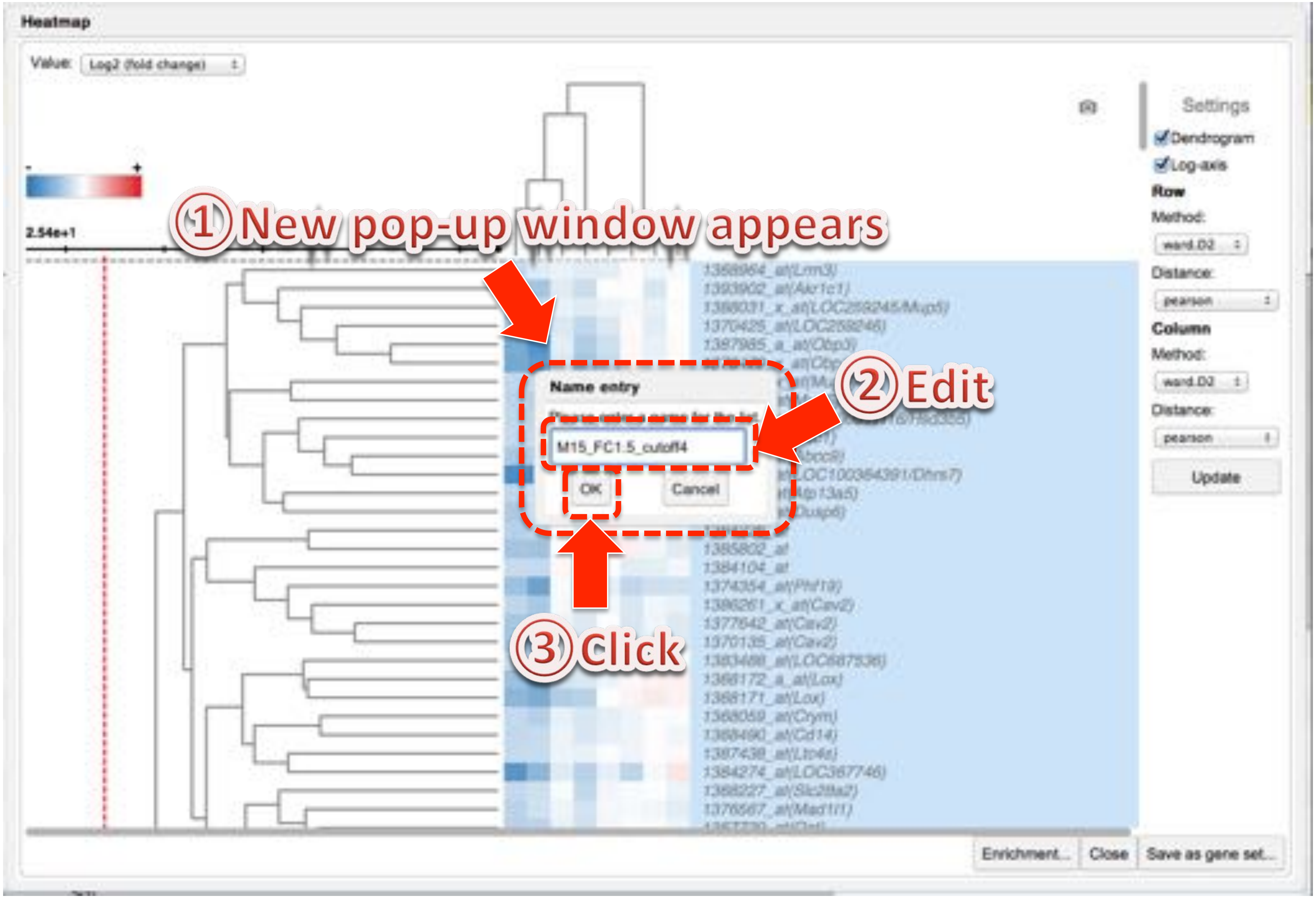
Heatmap
Value: Log2 (fold change)
Settings
Dendrogram
Log-axis
Row
Method:
ward.D2
Distance:
pearson
Column
Method:
ward.D2
Distance:
pearson
Update
2.54e+1
①New pop-up window appears
Name entry
M15_FC1.5_cutoff4
OK
Cancel
②Edit
③Click
Enrichment...
Close
Save as gene set...

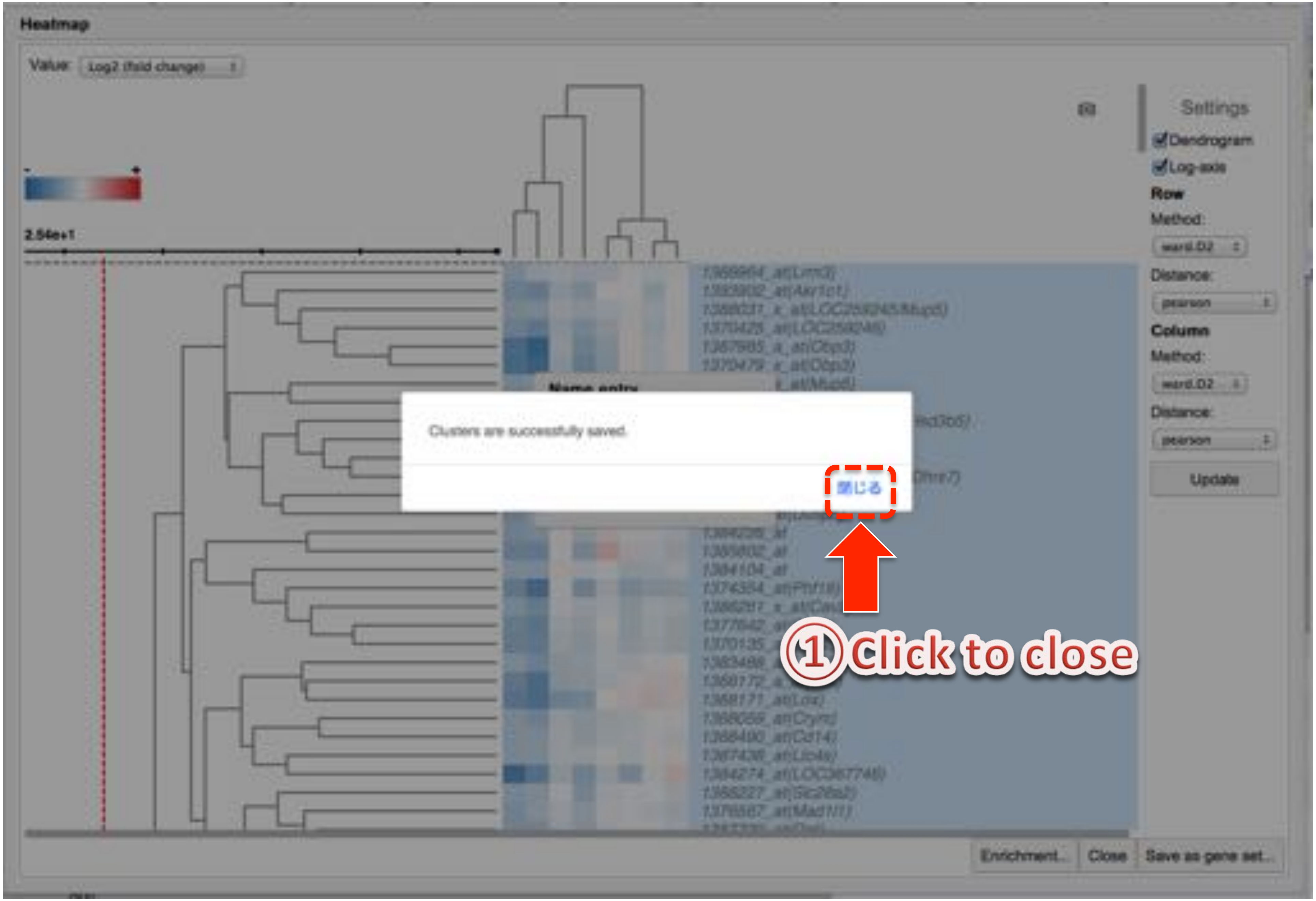
Heatmap
Value:
Log2 (fold change)
Settings
Dendrogram
Log-axis
Row
Method:
ward.D2
Distance:
pearson
Column
Method:
ward.D2
Distance:
pearson
Update
Clusters are successfully saved.
閉じる
①Click to close
Enrichment...
Close
Save as gene set...

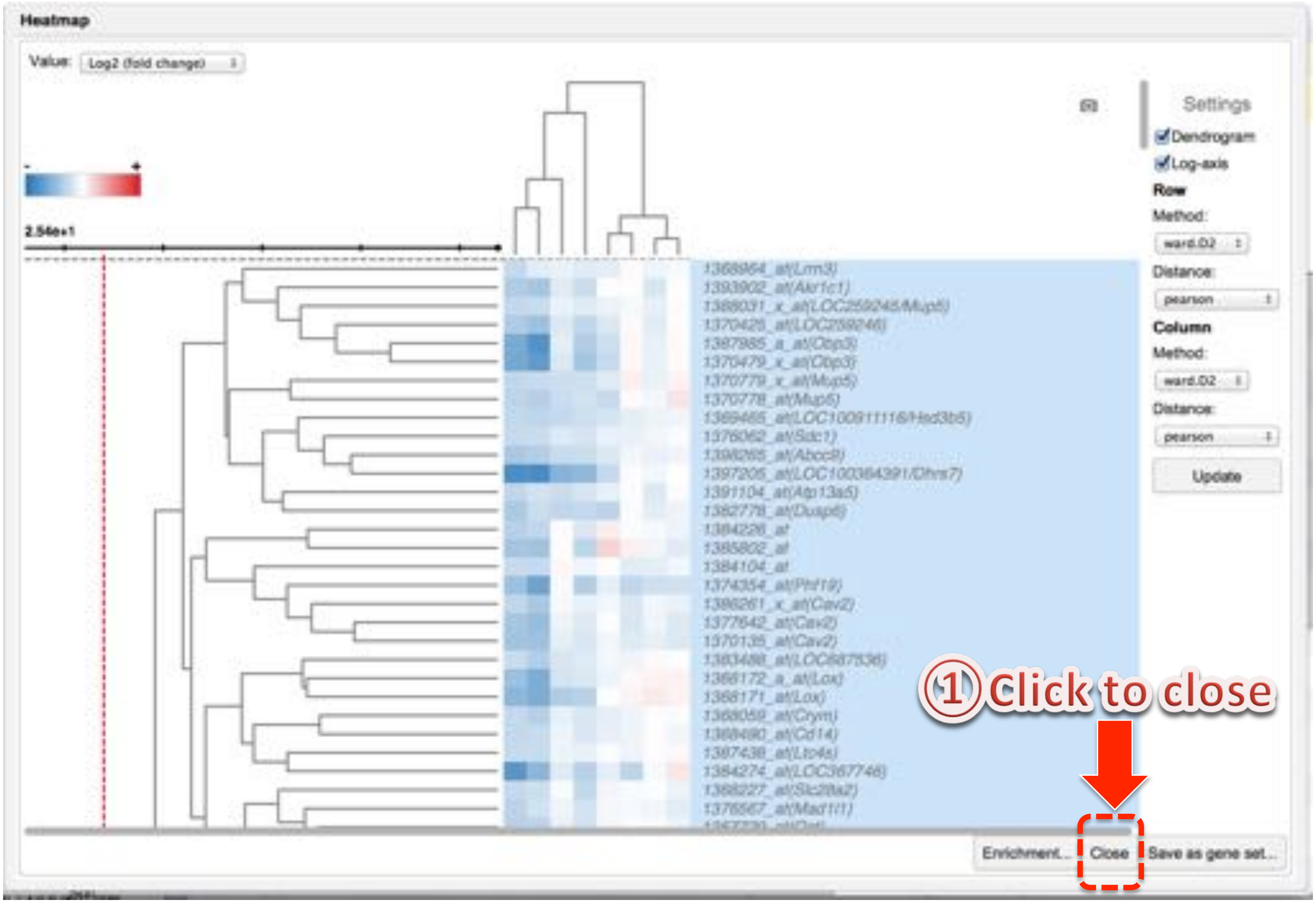
Heatmap
Value:
Log2 (fold change)
2.54e+1
Settings
Dendrogram
Log-axis
Row
Method:
ward.D2
Distance:
pearson
Column
Method:
ward.D2
Distance:
pearson
Update
①Click to close
Enrichment...
Close
Save as gene set...

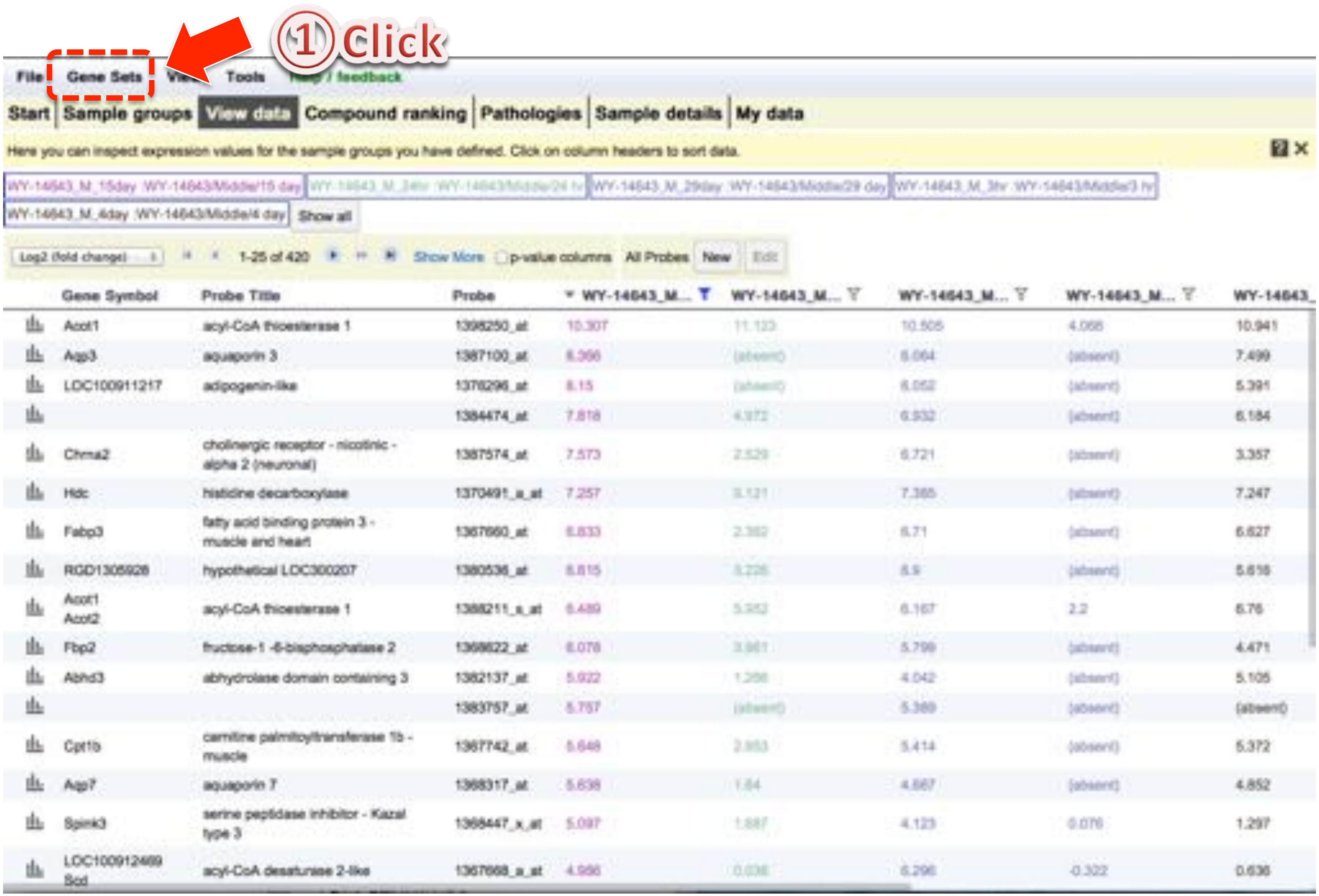
①Click
File
Gene Sets
Tools
Start
Sample groups
View data
Compound ranking
Pathologies
Sample details
My data
Here you can inspect expression values for the sample groups you have defined. Click on column headers to sort data.
Show all
1-25 of 420
Show More
p-value columns
All Probes
New
Edit
Gene Symbol
Probe Title
Probe
Acot1
acyl-CoA thioesterase 1
Aqp3
aquaporin 3
adipogenin-like
Chrna2
cholinergic receptor - nicotinic - alpha 2 (neuronal)
Hdc
histidine decarboxylase
Fabp3
fatty acid binding protein 3 - muscle and heart
Fbp2
Abhd3
abhydrolase domain containing 3
Cpt1b
carnitine palmitoyltransferase 1b - muscle
Aqp7
aquaporin 7
Spink3
serine peptidase inhibitor - Kazal type 3
acyl-CoA desaturase 2-like

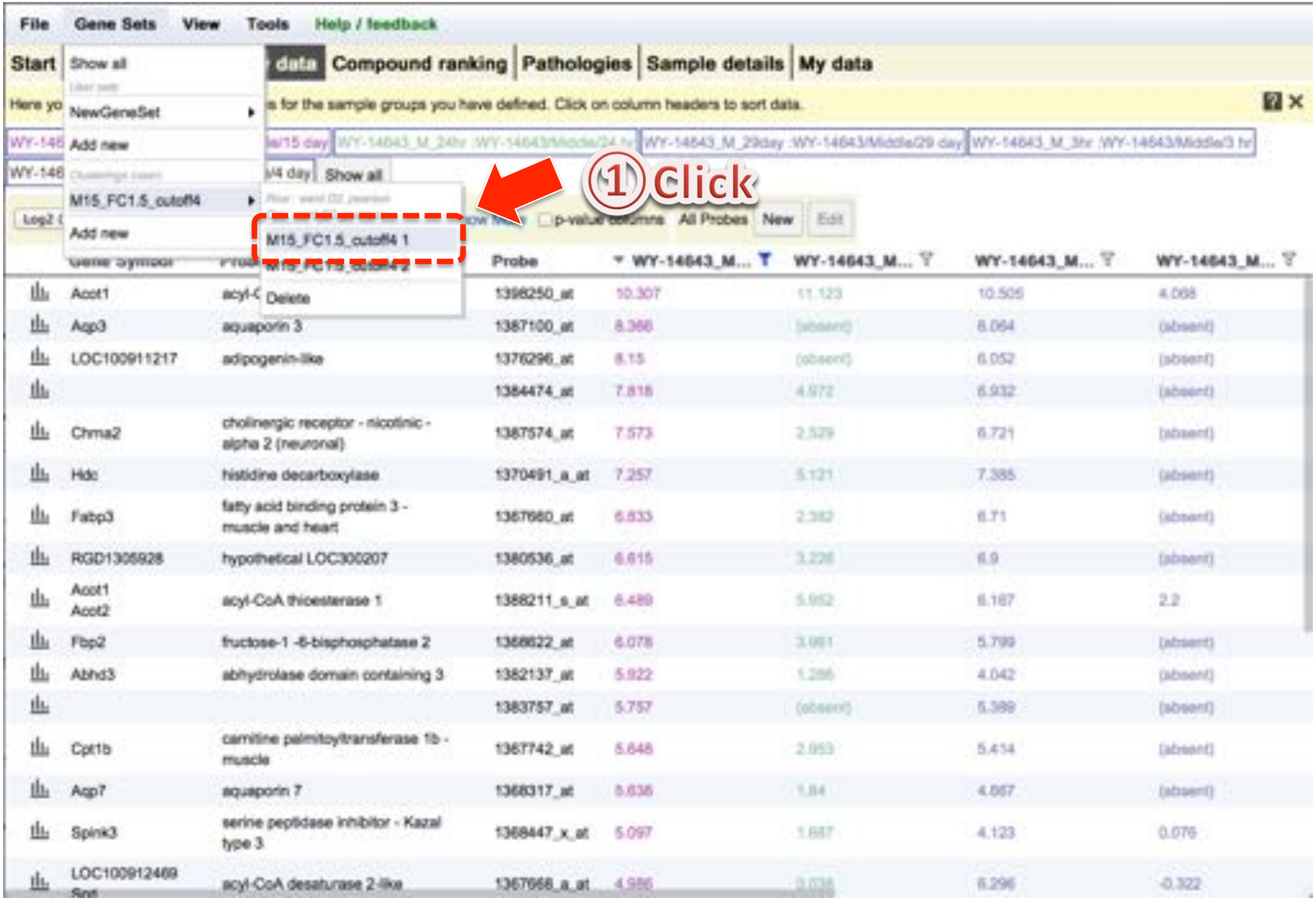
File
Gene Sets
View
Tools
Help / feedback
Start
Show all
Compound ranking
Pathologies
Sample details
My data
NewGeneSet
Add new
M15_FC1.5_cutoff4
M15_FC1.5_cutoff4 1
Delete
①Click
Show all
p-value columns
All Probes
New
Edit
Probe
WY-14643_M...
Acot1
aquaporin 3
adipogenin-like
histidine decarboxylase
fatty acid binding protein 3 - muscle and heart
hypothetical LOC300207
acyl-CoA thioesterase 1
fructose-1 -6-bisphosphatase 2
abhydrolase domain containing 3
aquaporin 7
acyl-CoA desaturase 2-like

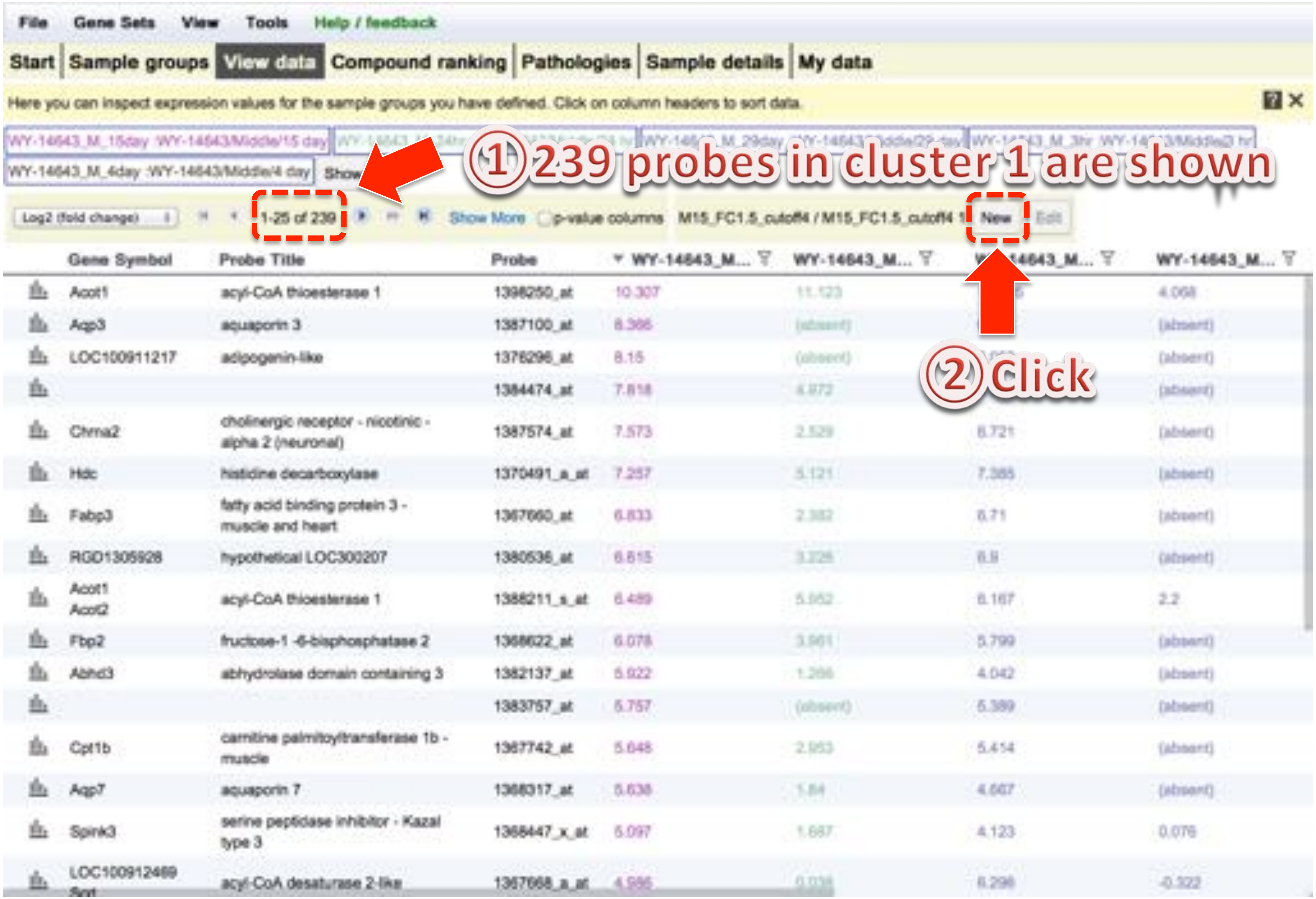
File
Gene Sets
View
Tools
Help / feedback
Start
Sample groups
View data
Compound ranking
Pathologies
Sample details
My data
Here you can inspect expression values for the sample groups you have defined. Click on column headers to sort data.
①239 probes in cluster 1 are shown
Log2 (fold change)
1-25 of 239
Show More
p-value columns
New
Edit
②Click
Gene Symbol
Probe Title
Probe
Acot1
acyl-CoA thioesterase 1
Aqp3
aquaporin 3
LOC100911217
adipogenin-like
Chrna2
cholinergic receptor - nicotinic - alpha 2 (neuronal)
Hdc
histidine decarboxylase
Fabp3
fatty acid binding protein 3 - muscle and heart
RGD1305928
hypothetical LOC300207
Acot1
Acot2
acyl-CoA thioesterase 1
Fbp2
fructose-1 -6-bisphosphatase 2
Abhd3
abhydrolase domain containing 3
Cpt1b
carnitine palmitoyltransferase 1b - muscle
Aqp7
aquaporin 7
Spink3
serine peptidase inhibitor - Kazal type 3
LOC100912469
acyl-CoA desaturase 2-like

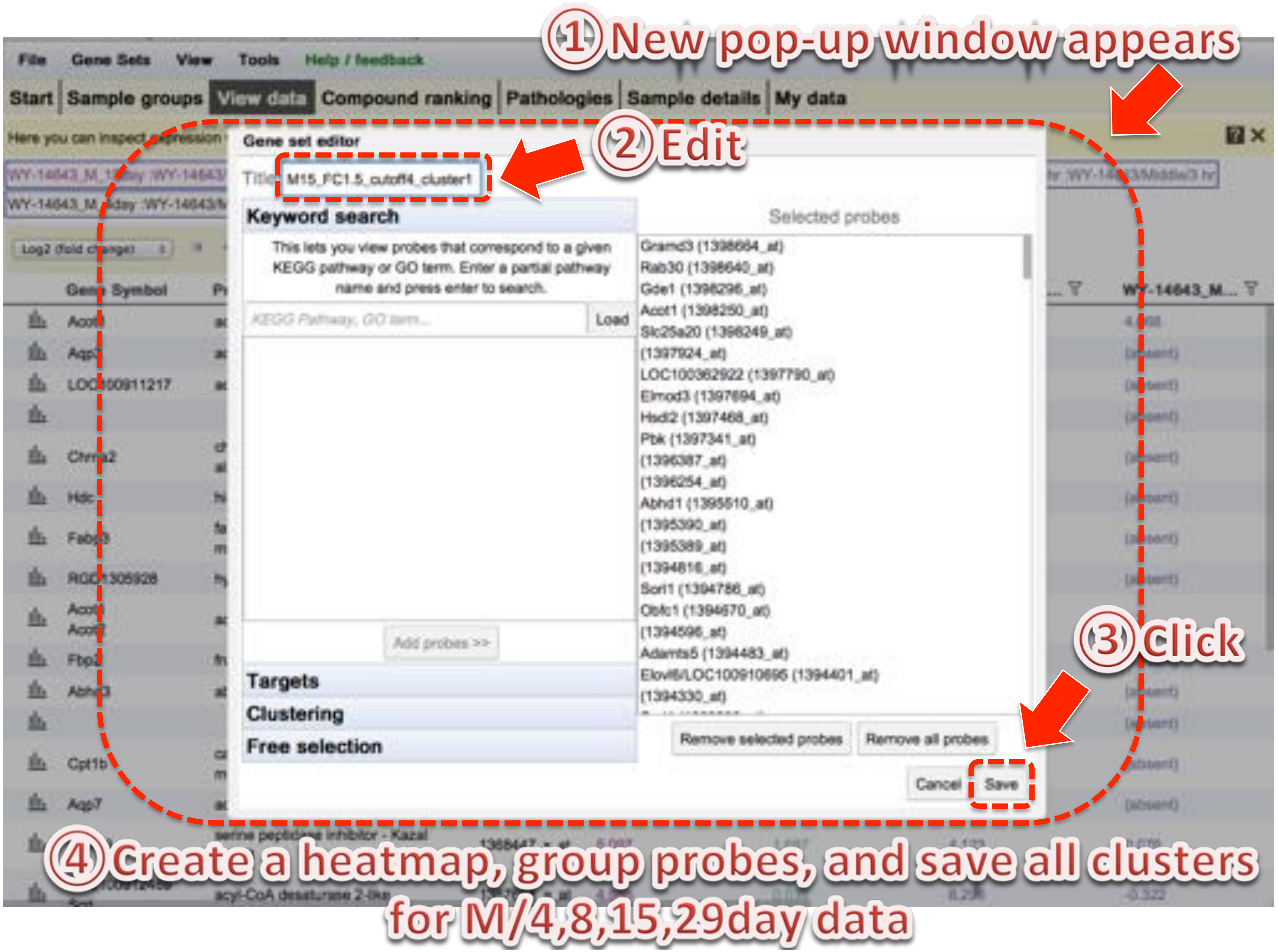
①New pop-up window appears
File
Gene Sets
View
Tools
Help / feedback
Start
Sample groups
View data
Compound ranking
Pathologies
Sample details
My data
②Edit
Gene set editor
Title
M15_FC1.5_cutoff4_cluster1
Keyword search
This lets you view probes that correspond to a given KEGG pathway or GO term. Enter a partial pathway name and press enter to search.
KEGG Pathway, GO term...
Load
Add probes >>
Targets
Clustering
Free selection
Selected probes
Gramd3 (1398664_at)
Rab30 (1398640_at)
Gde1 (1398296_at)
Acot1 (1398250_at)
Slc25a20 (1398249_at)
(1397924_at)
LOC100362922 (1397790_at)
Elmod3 (1397694_at)
Hsd2 (1397468_at)
Pbk (1397341_at)
(1396387_at)
(1396254_at)
Abhd1 (1395510_at)
(1395390_at)
(1395389_at)
(1394816_at)
Sorl1 (1394786_at)
Cbfc1 (1394670_at)
(1394596_at)
Adamts5 (1394483_at)
Elovl6/LOC100910695 (1394401_at)
(1394330_at)
Remove selected probes
Remove all probes
③Click
Cancel
Save
Gene Symbol
Acot1
Aqp7
LOC100911217
Chrna2
Hdc
Fabp3
RGD1305928
Fbp2
Abhd3
Cpt1b
Aqp7
④Create a heatmap, group probes, and save all clusters for M/4,8,15,29day data

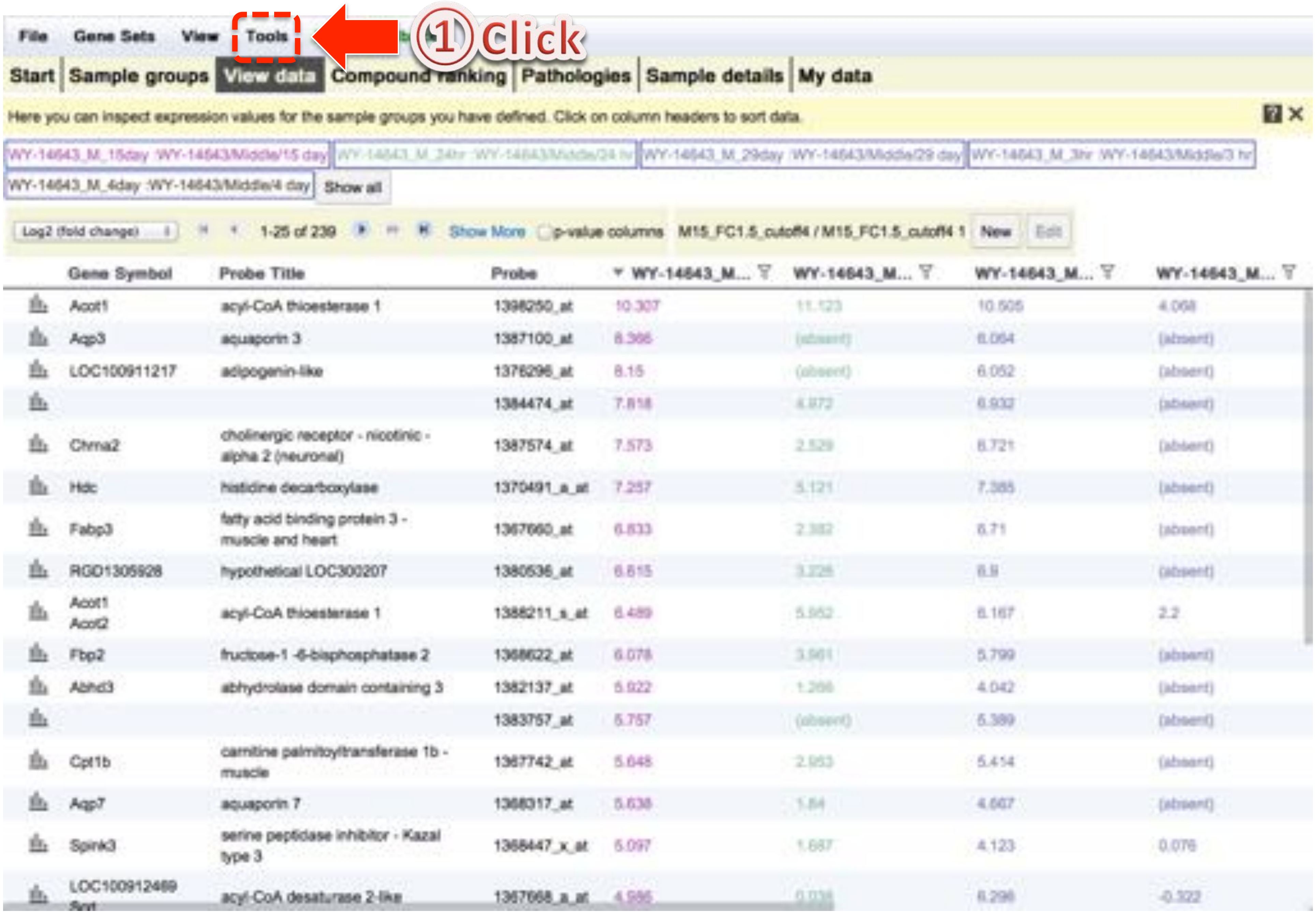

Gene Symbol	Probe Title	Probe	WY-14643_M...	WY-14643_M...	WY-14643_M...	WY-14643_M...
Acot1	acyl-CoA thioesterase 1	1398250_at	10.307	11.123	10.505	4.068
Aqp3	aquaporin 3	1387100_at	8.366	(absent)	8.064	(absent)
LOC100911217	adipogenin-like	1376296_at	8.15	(absent)	6.052	(absent)
		1384474_at	7.818	4.872	6.932	(absent)
Chrna2	cholinergic receptor - nicotinic - alpha 2 (neuronal)	1387574_at	7.573	2.529	6.721	(absent)
Hdc	histidine decarboxylase	1370491_a_at	7.257	5.121	7.385	(absent)
Fabp3	fatty acid binding protein 3 - muscle and heart	1367660_at	6.833	2.382	6.71	(absent)
RGD1305928	hypothetical LOC300207	1380536_at	6.815	3.226	6.8	(absent)
Acot1 Acot2	acyl-CoA thioesterase 1	1388211_s_at	6.489	5.952	6.167	2.2
Fbp2	fructose-1 -6-bisphosphatase 2	1368622_at	6.078	3.961	5.799	(absent)
Abhd3	abhydrolase domain containing 3	1382137_at	5.922	1.266	4.042	(absent)
		1383757_at	5.757	(absent)	5.389	(absent)
Cpt1b	carnitine palmitoyltransferase 1b - muscle	1367742_at	5.648	2.953	5.414	(absent)
Aqp7	aquaporin 7	1368317_at	5.638	1.84	4.667	(absent)
Spink3	serine peptidase inhibitor - Kazal type 3	1368447_x_at	5.097	1.687	4.123	0.076
LOC100912469 Scd	acyl-CoA desaturase 2-like	1367668_a_at	4.986	0.038	6.298	-0.322

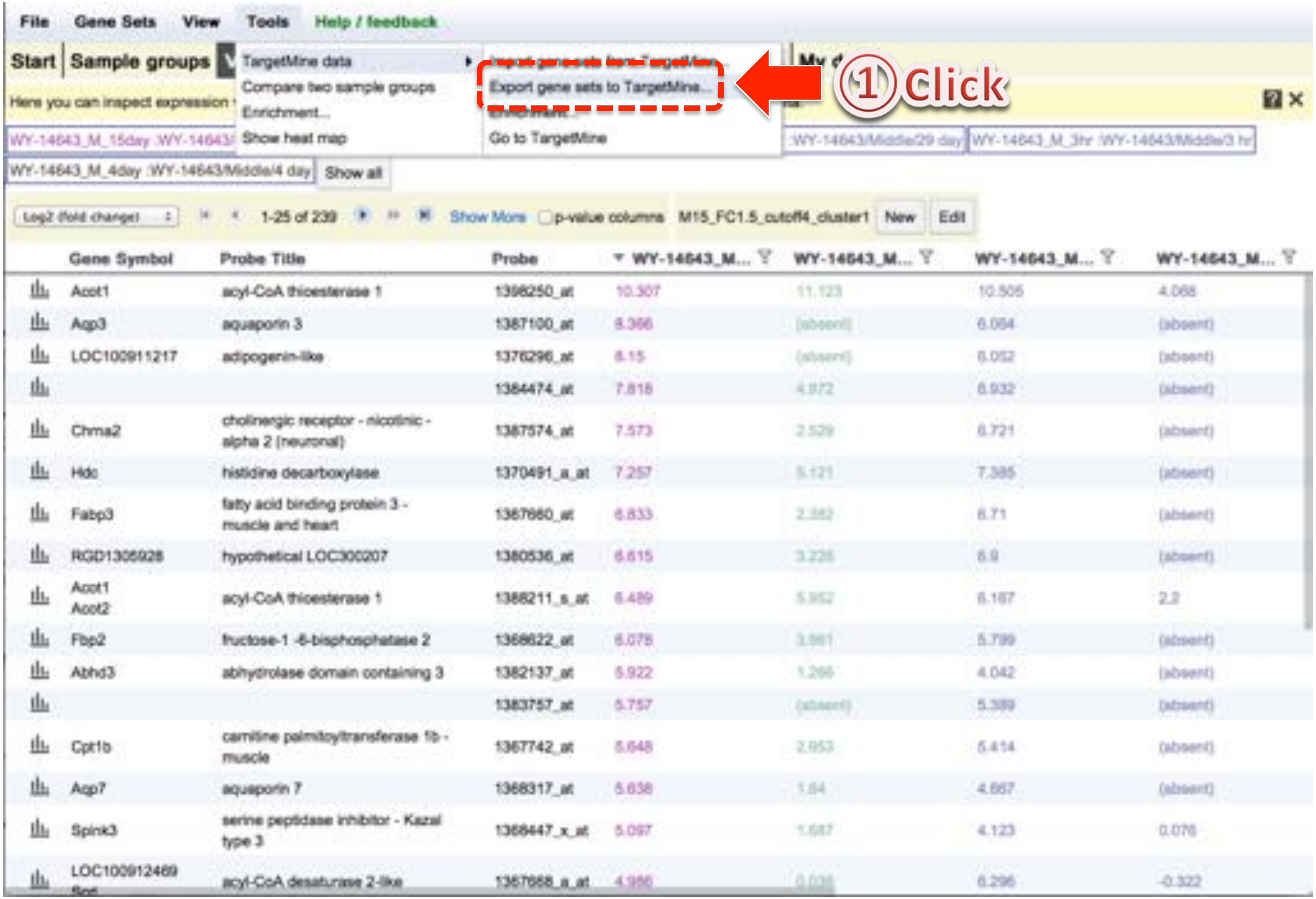
File
Gene Sets
View
Tools
Help / feedback
Start
Sample groups
TargetMine data
Compare two sample groups
Enrichment...
Show heat map
Export gene sets to TargetMine...
Go to TargetMine
①Click
Here you can inspect expression
WY-14643_M_4day :WY-14643/Middle/4 day
Show all
1-25 of 239
Show More
p-value columns
M15_FC1.5_cutoff4_cluster1
New
Edit
Gene Symbol
Probe Title
Probe
Acot1 acyl-CoA thioesterase 1 1398250_at
Aqp3 aquaporin 3 1387100_at
LOC100911217 adipogenin-like 1376296_at
1384474_at
Chrna2 cholinergic receptor - nicotinic - alpha 2 (neuronal) 1387574_at
Hdc histidine decarboxylase 1370491_a_at
Fabp3 fatty acid binding protein 3 - muscle and heart 1367660_at
RGD1306928 hypothetical LOC300207 1380536_at
Acot1 Acot2 acyl-CoA thioesterase 1 1388211_s_at
Fbp2 fructose-1 -6-bisphosphatase 2 1368622_at
Abhd3 abhydrolase domain containing 3 1382137_at
1383757_at
Cpt1b carnitine palmitoyltransferase 1b - muscle 1367742_at
Aqp7 aquaporin 7 1368317_at
Spink3 serine peptidase inhibitor - Kazal type 3 1368447_x_at
LOC100912469 acyl-CoA desaturase 2-like 1367668_a_at

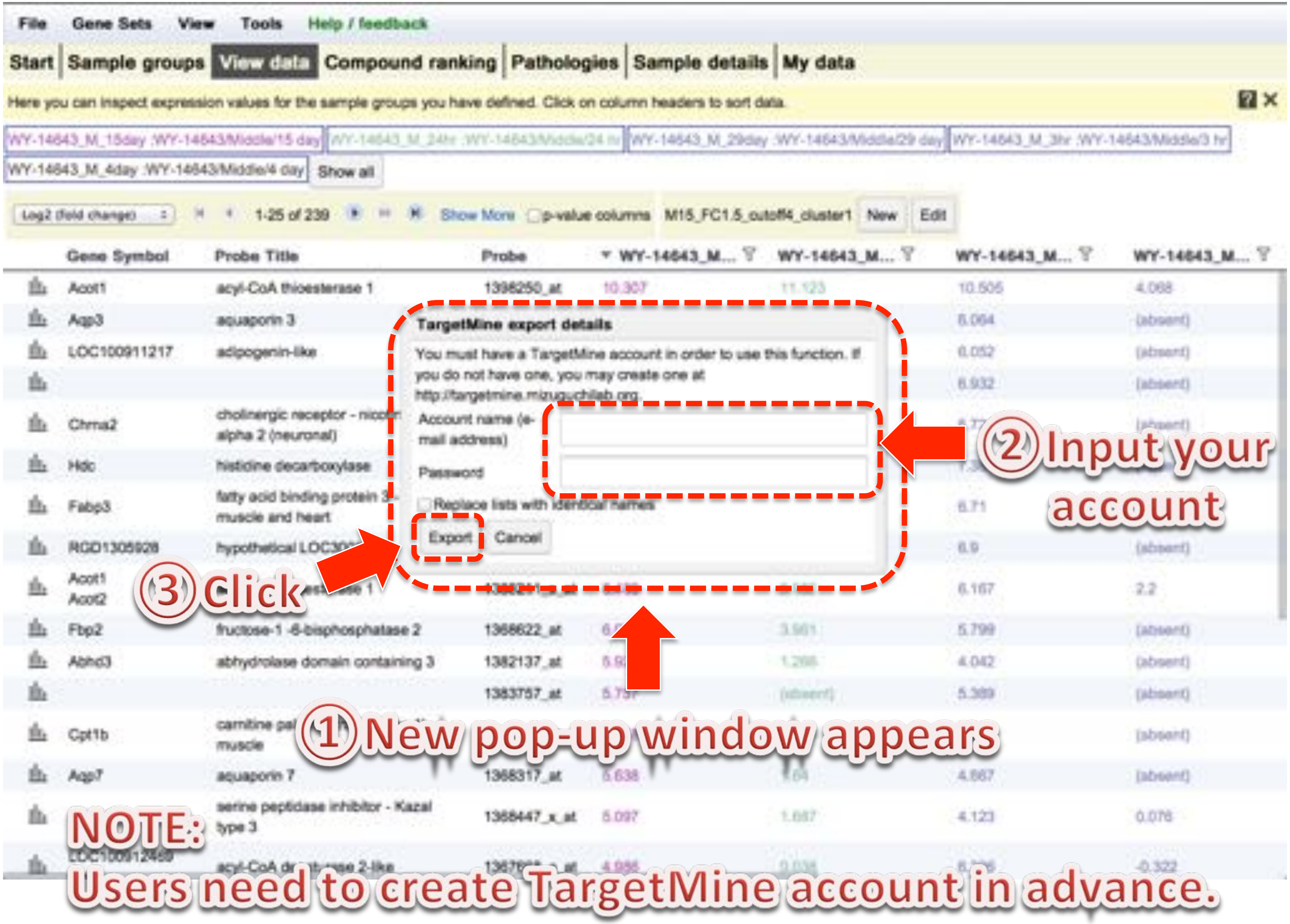
File
Gene Sets
View
Tools
Help / feedback
Start
Sample groups
View data
Compound ranking
Pathologies
Sample details
My data
Here you can inspect expression values for the sample groups you have defined. Click on column headers to sort data.
Show all
1-25 of 239
Show More
p-value columns
M15_FC1.5_cutoff4_cluster1
New
Edit
Gene Symbol
Probe Title
Probe
TargetMine export details
You must have a TargetMine account in order to use this function. If you do not have one, you may create one at http://targetmine.mizuguchilab.org.
Account name (e-mail address)
Password
Replace lists with identical names
Export
Cancel
②Input your account
③Click
①New pop-up window appears
NOTE:
Users need to create TargetMine account in advance.

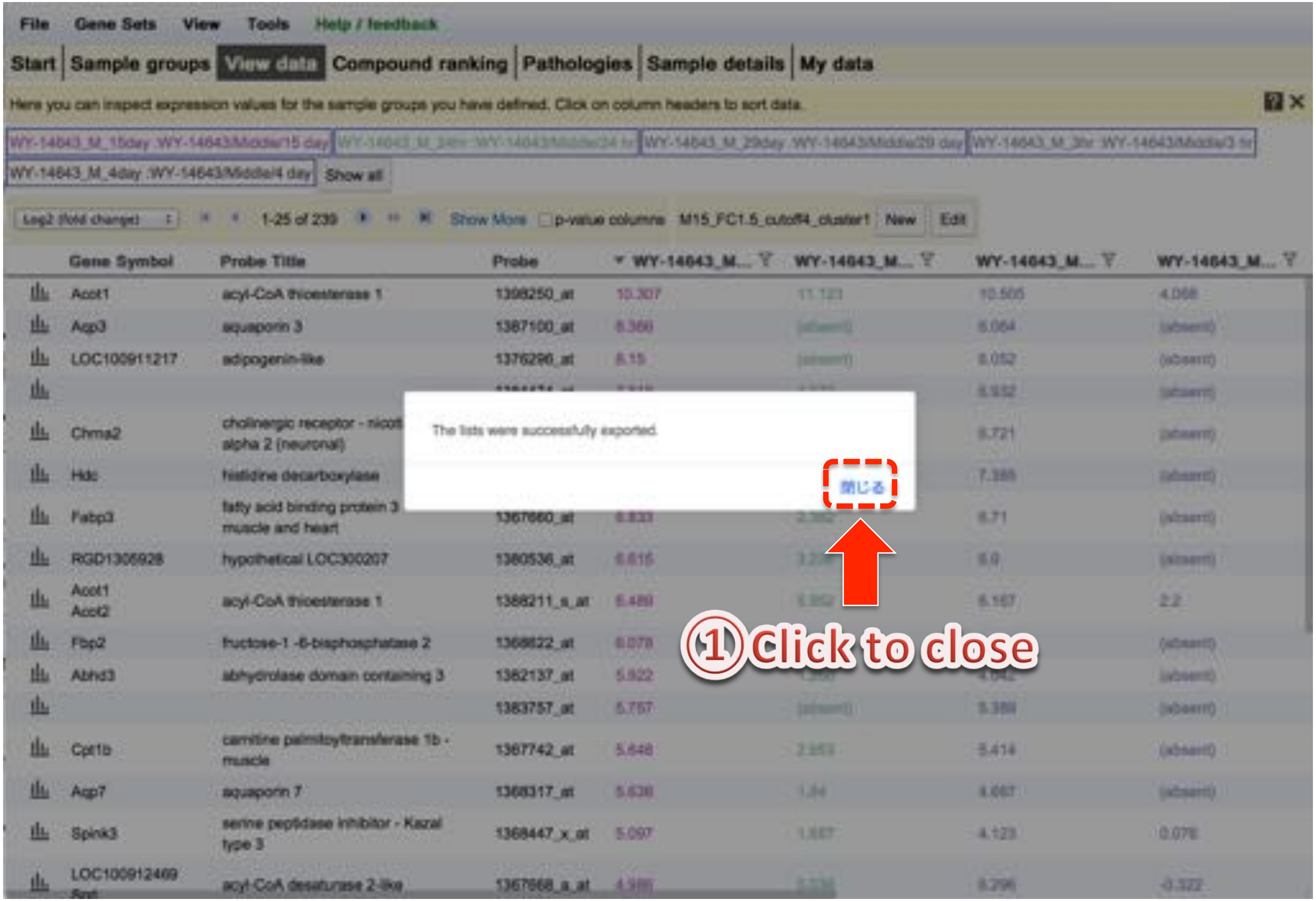
File
Gene Sets
View
Tools
Help / feedback
Start
Sample groups
View data
Compound ranking
Pathologies
Sample details
My data
Here you can inspect expression values for the sample groups you have defined. Click on column headers to sort data.
Show all
1-25 of 239
Show More
p-value columns
M15_FC1.5_cutoff4_cluster1
New
Edit
Gene Symbol
Probe Title
Probe
The lists were successfully exported.
閉じる
① Click to close

①Open TargetMine (targetmine.mizuguchilab.org)

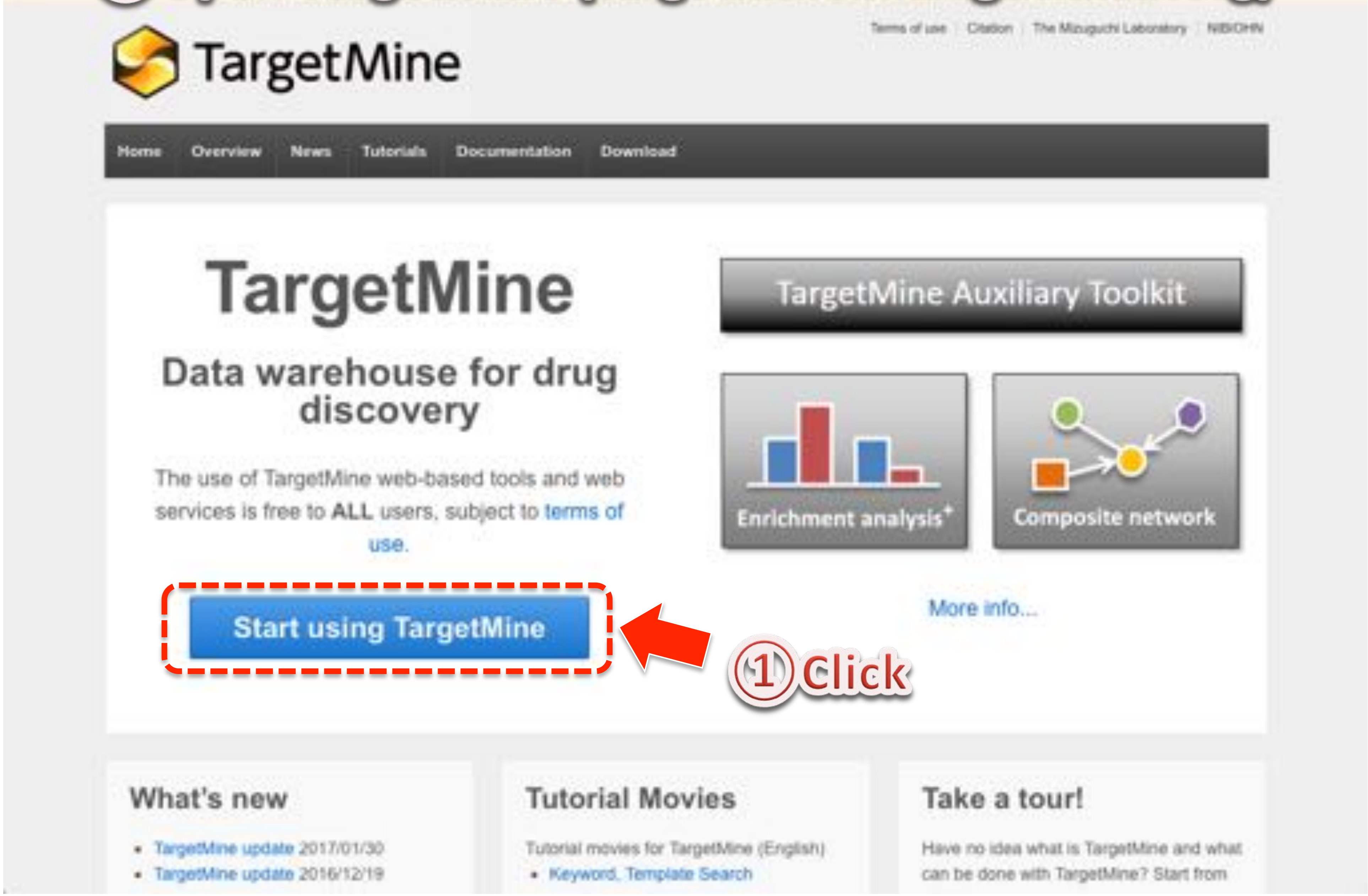

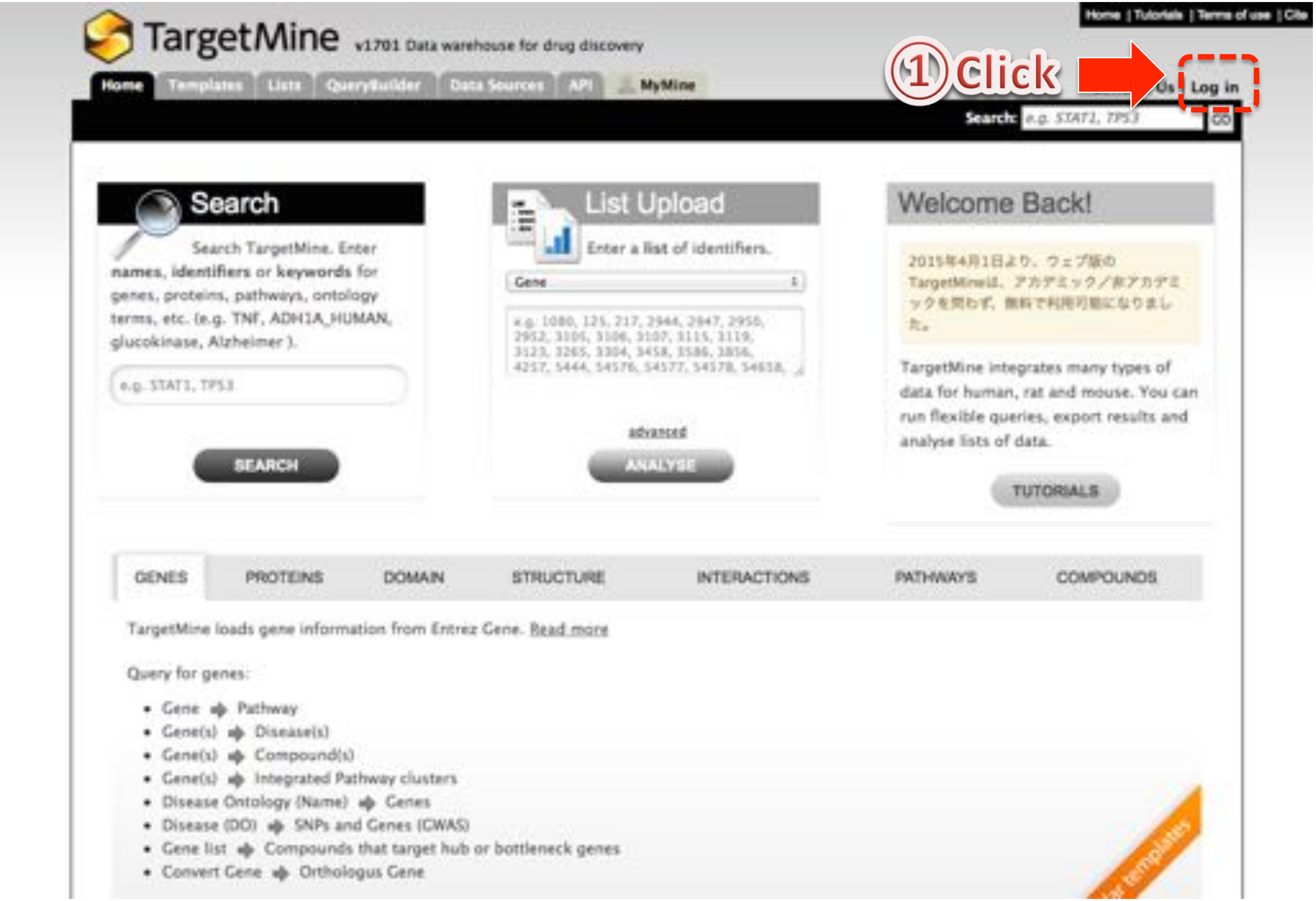

TargetMine v1701 Data warehouse for drug discovery
Home | Tutorials | Terms of use | Cite
①Click
Log in
Home
Templates
Lists
QueryBuilder
Data Sources
API
MyMine
Search:
e.g. STAT1, TP53
GO
Search
Search TargetMine. Enter names, identifiers or keywords for genes, proteins, pathways, ontology terms, etc. (e.g. TNF, ADH1A_HUMAN, glucokinase, Alzheimer).
e.g. STAT1, TP53
SEARCH
List Upload
Enter a list of identifiers.
Gene
e.g. 1080, 125, 217, 2944, 2947, 2950, 2952, 3105, 3106, 3107, 3115, 3119, 3123, 3265, 3304, 3458, 3586, 3856, 4257, 5444, 54576, 54577, 54578, 54658,
advanced
ANALYSE
Welcome Back!
2015年4月1日より、ウェブ版のTargetMineは、アカデミック／非アカデミックを問わず、無料で利用可能になりました。
TargetMine integrates many types of data for human, rat and mouse. You can run flexible queries, export results and analyse lists of data.
TUTORIALS
GENES
PROTEINS
DOMAIN
STRUCTURE
INTERACTIONS
PATHWAYS
COMPOUNDS
TargetMine loads gene information from Entrez Gene. Read more
Query for genes:
Gene ➜ Pathway
Gene(s) ➜ Disease(s)
Gene(s) ➜ Compound(s)
Gene(s) ➜ Integrated Pathway clusters
Disease Ontology (Name) ➜ Genes
Disease (DO) ➜ SNPs and Genes (GWAS)
Gene list ➜ Compounds that target hub or bottleneck genes
Convert Gene ➜ Orthologus Gene

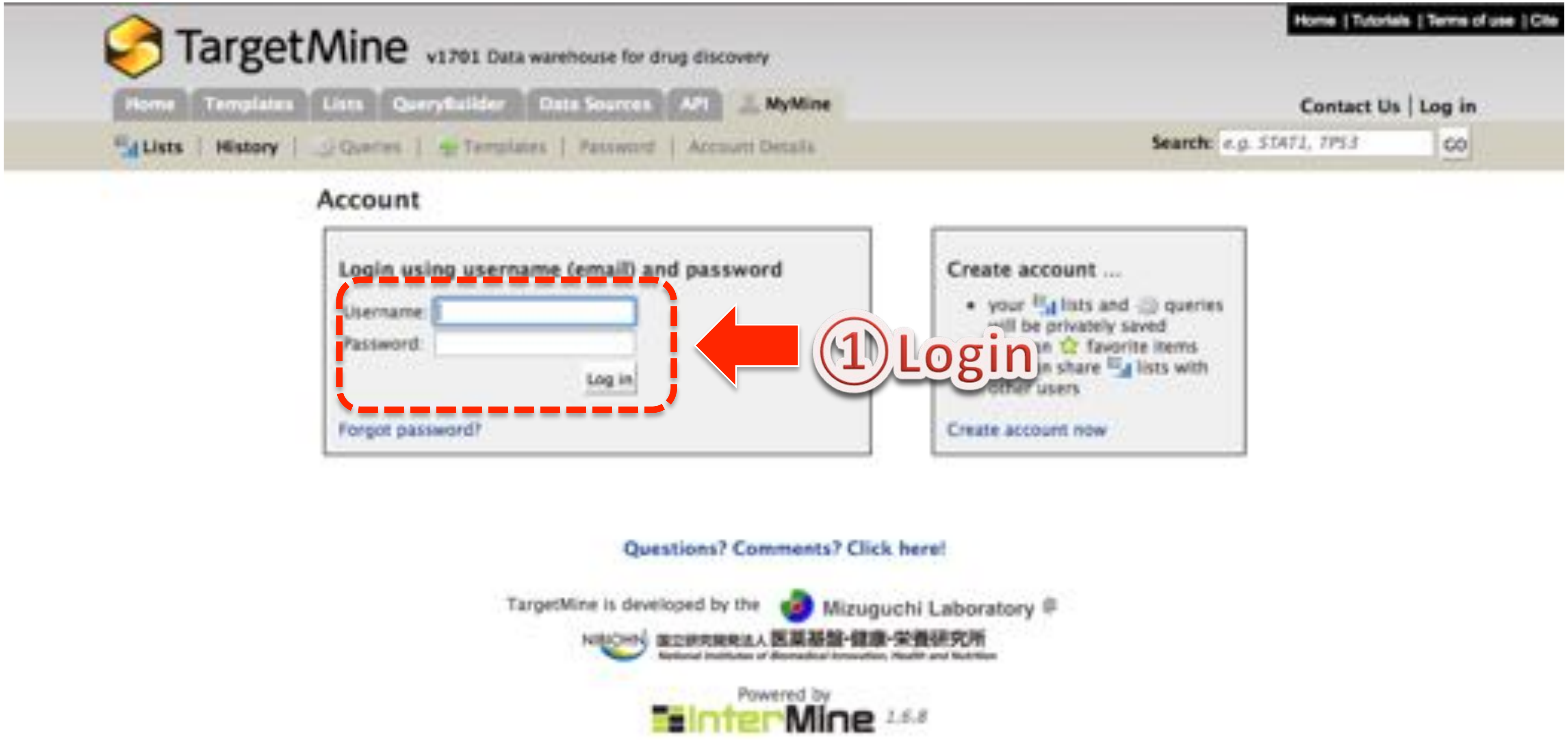
TargetMine v1701 Data warehouse for drug discovery
Home | Tutorials | Terms of use | Cite
Home
Templates
Lists
QueryBuilder
Data Sources
API
MyMine
Contact Us | Log in
Lists | History | Queries | Templates | Password | Account Details
Search: e.g. STAT1, TP53
GO
Account
Login using username (email) and password
Username:
Password:
Log in
Forgot password?
①Login
Create account ...
Create account now
Questions? Comments? Click here!
TargetMine is developed by the Mizuguchi Laboratory
Powered by InterMine 1.6.8

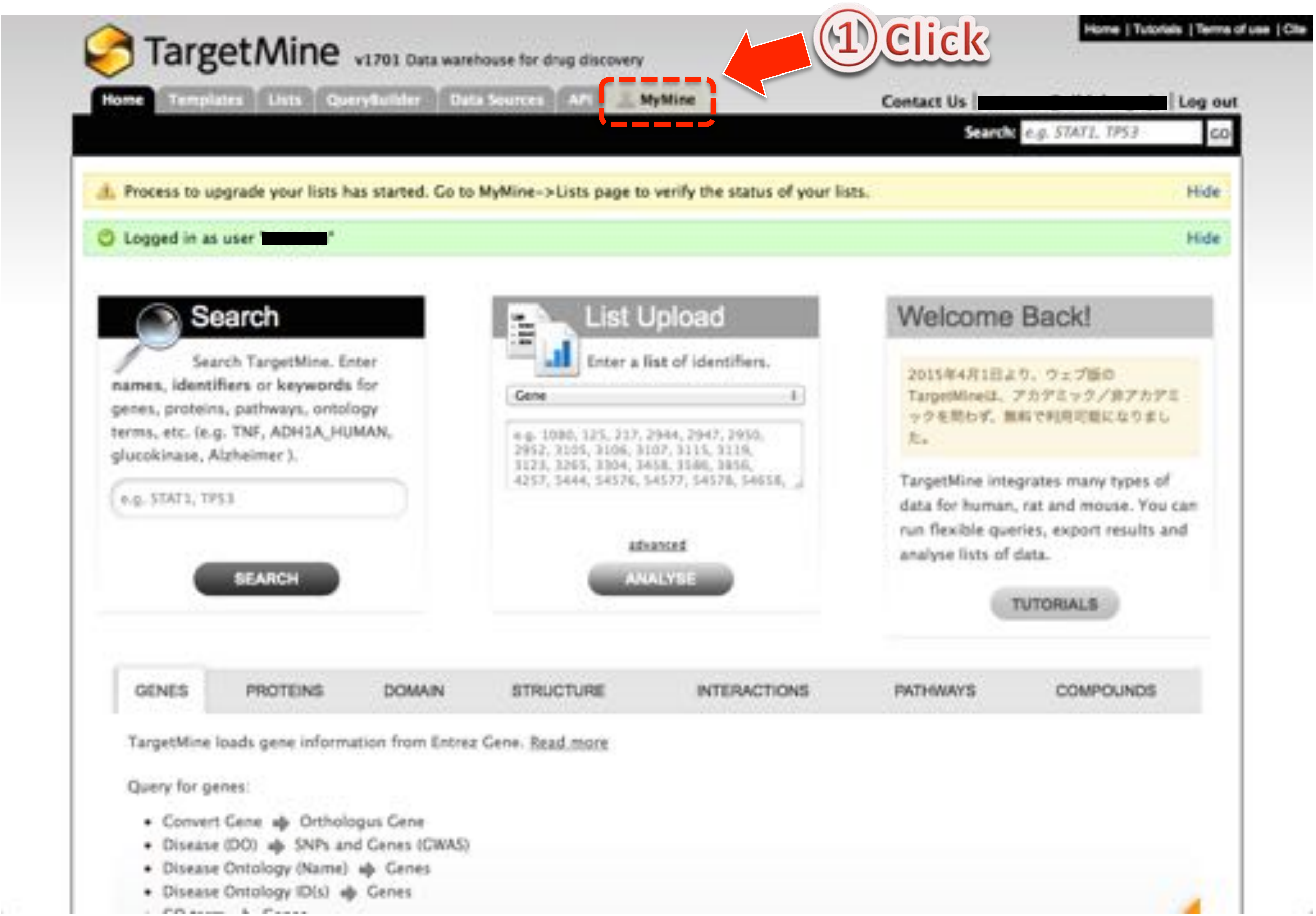
①Click
Home | Tutorials | Terms of use | Cite
TargetMine v1701 Data warehouse for drug discovery
Home
Templates
Lists
QueryBuilder
Data Sources
API
MyMine
Contact Us | [redacted] | Log out
Search: e.g. STAT1, TP53 GO
Process to upgrade your lists has started. Go to MyMine->Lists page to verify the status of your lists.
Hide
Logged in as user "[redacted]"
Hide
Search
Search TargetMine. Enter names, identifiers or keywords for genes, proteins, pathways, ontology terms, etc. (e.g. TNF, ADH1A_HUMAN, glucokinase, Alzheimer).
e.g. STAT1, TP53
SEARCH
List Upload
Enter a list of identifiers.
Gene
e.g. 1080, 125, 217, 2944, 2947, 2950, 2952, 3105, 3106, 3107, 3115, 3119, 3123, 3265, 3304, 3458, 3586, 3856, 4257, 5444, 54576, 54577, 54578, 54658,
advanced
ANALYSE
Welcome Back!
2015年4月1日より、ウェブ版のTargetMineは、アカデミック／非アカデミックを問わず、無料で利用可能になりました。
TargetMine integrates many types of data for human, rat and mouse. You can run flexible queries, export results and analyse lists of data.
TUTORIALS
GENES
PROTEINS
DOMAIN
STRUCTURE
INTERACTIONS
PATHWAYS
COMPOUNDS
TargetMine loads gene information from Entrez Gene. Read more
Query for genes:
Convert Gene → Orthologus Gene
Disease (DO) → SNPs and Genes (GWAS)
Disease Ontology (Name) → Genes
Disease Ontology ID(s) → Genes

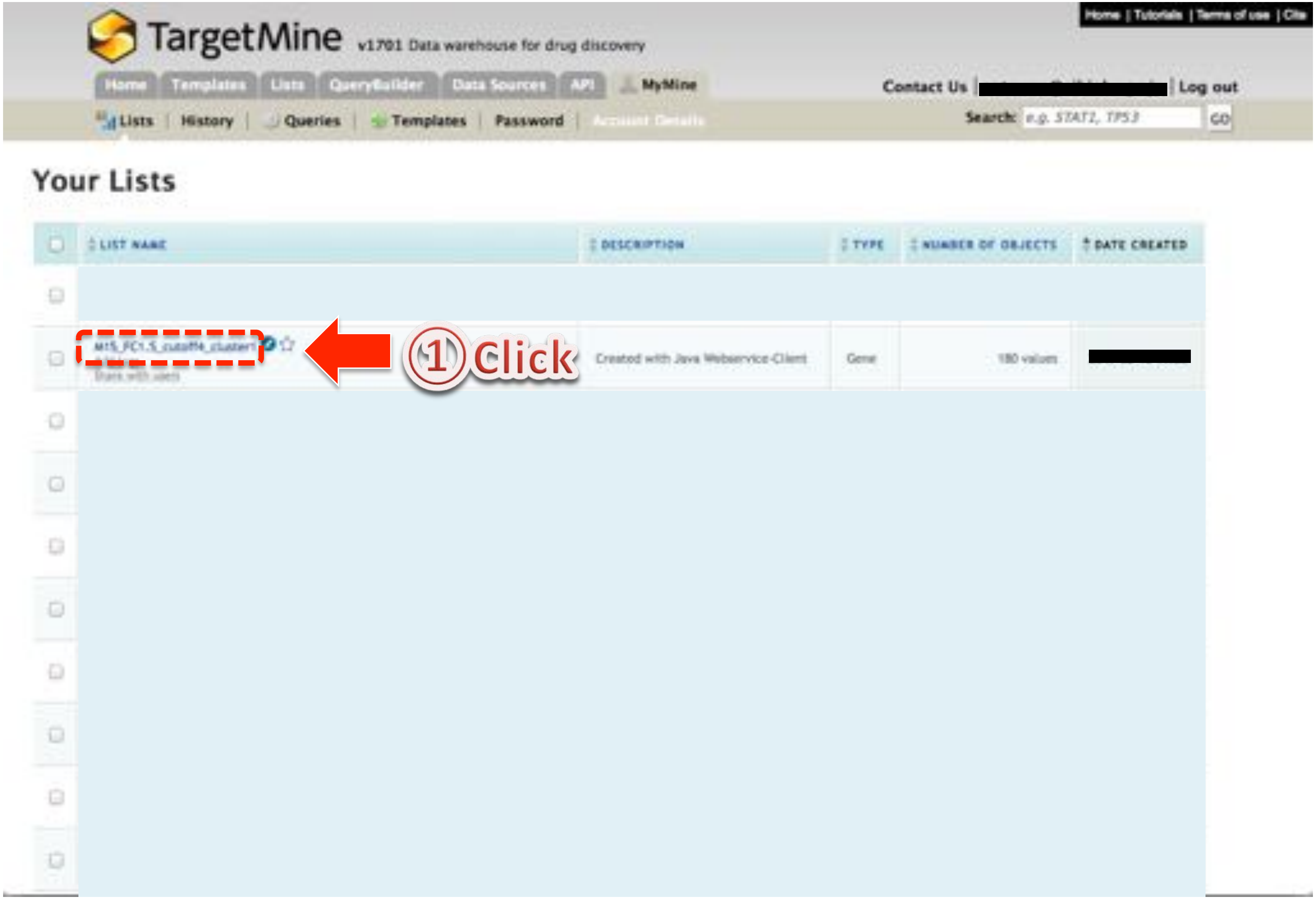
Home | Tutorials | Terms of use | Cite
TargetMine
v1701 Data warehouse for drug discovery
Home
Templates
Lists
QueryBuilder
Data Sources
API
MyMine
Contact Us
[redacted]
Log out
Lists
History
Queries
Templates
Password
Account Details
Search:
e.g. STAT2, TP53
GO
Your Lists
LIST NAME
DESCRIPTION
TYPE
NUMBER OF OBJECTS
DATE CREATED
Created with Java Webservice Client
Gene
180 values
[redacted]
①Click

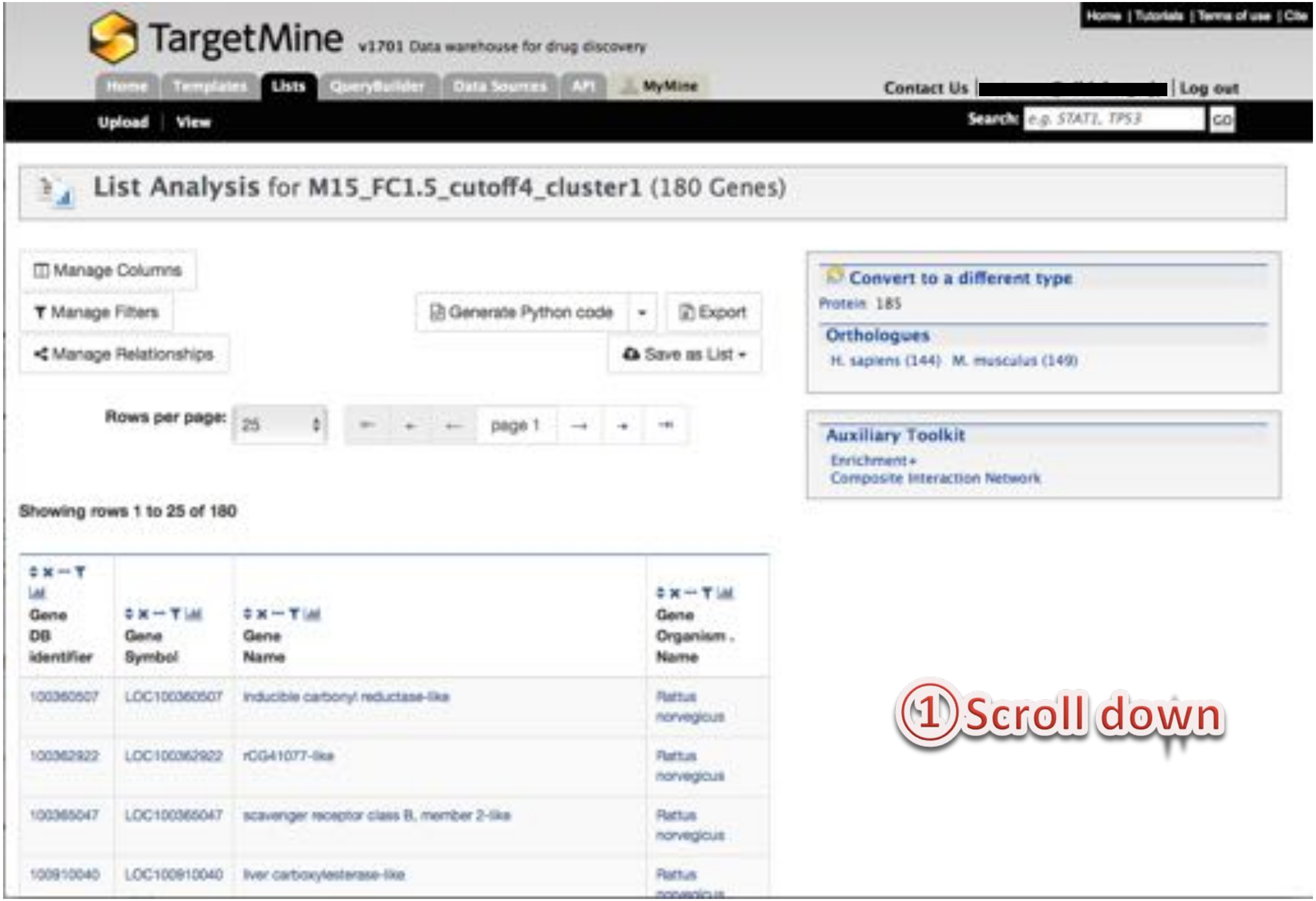
Home | Tutorials | Terms of use | Cite
TargetMine v1701 Data warehouse for drug discovery
Home
Templates
Lists
QueryBuilder
Data Sources
API
MyMine
Contact Us | [redacted] | Log out
Upload | View
Search: e.g. STAT1, TP53
GO
List Analysis for M15_FC1.5_cutoff4_cluster1 (180 Genes)
Manage Columns
Manage Filters
Manage Relationships
Generate Python code
Export
Save as List
Rows per page: 25
page 1
Showing rows 1 to 25 of 180
Gene DB identifier
Gene Symbol
Gene Name
Gene Organism . Name
100360507 LOC100360507 inducible carbonyl reductase-like Rattus norvegicus
100362922 LOC100362922 rCG41077-like Rattus norvegicus
100365047 LOC100365047 scavenger receptor class B, member 2-like Rattus norvegicus
100910040 LOC100910040 liver carboxylesterase-like Rattus norvegicus
Convert to a different type
Protein 185
Orthologues
H. sapiens (144) M. musculus (149)
Auxiliary Toolkit
Enrichment+
Composite Interaction Network
①Scroll down

NOTE:

Since TargetMine updates monthly, the result can be slightly different from that in Suupelemnatry Table 2

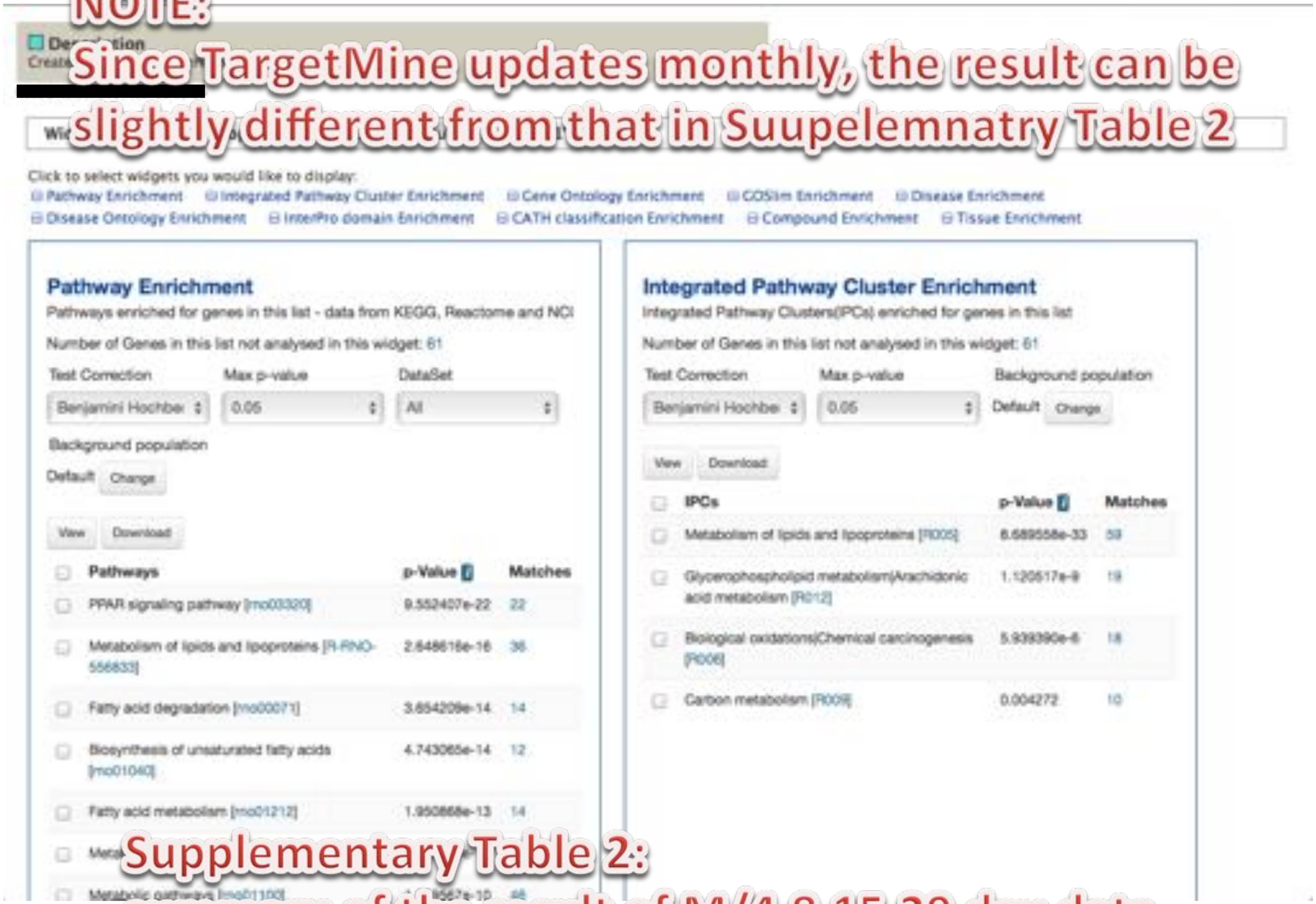

Supplementary Table 2:
summary of the result of M/4,8,15,29 day data.

Compound ranking

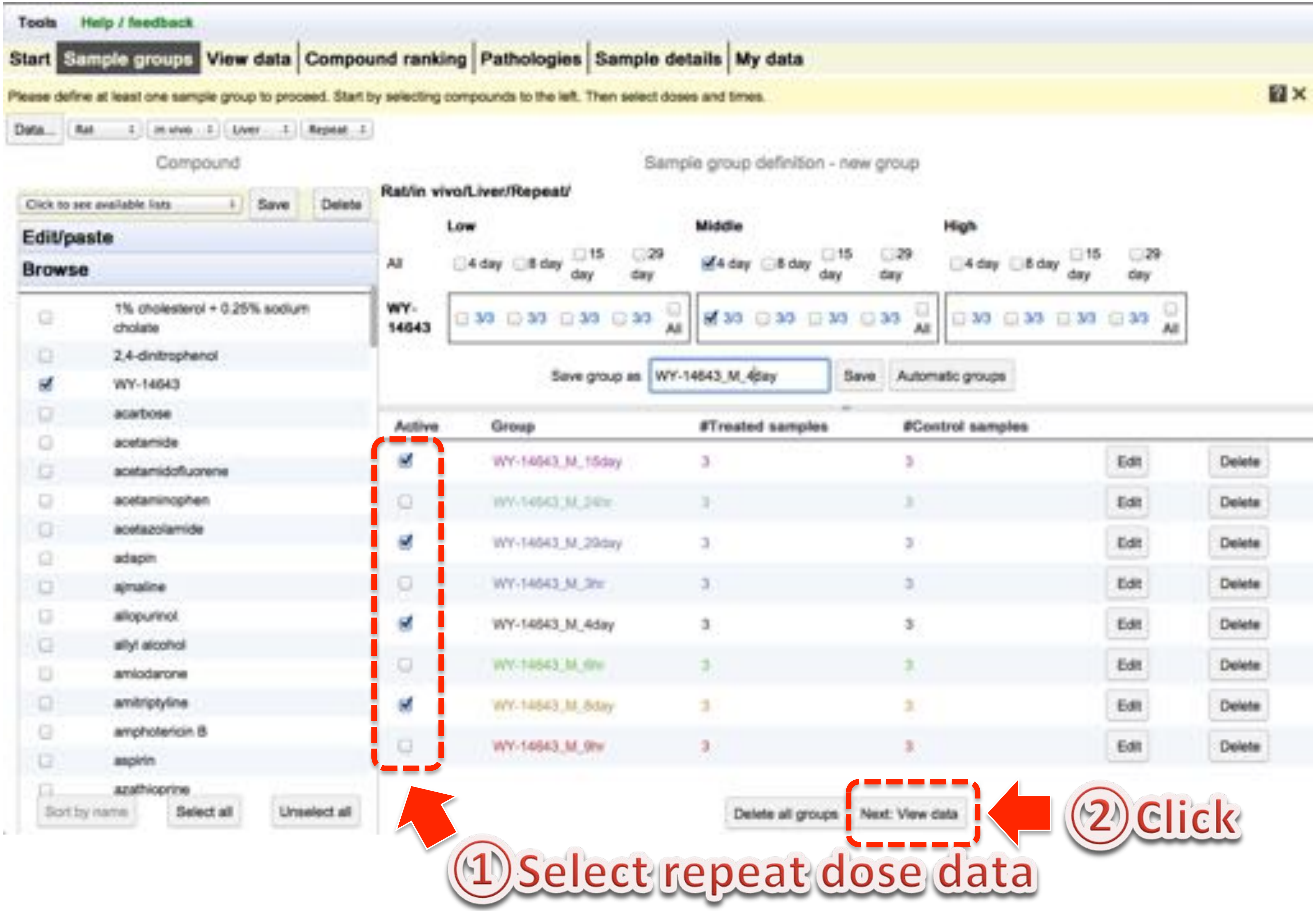

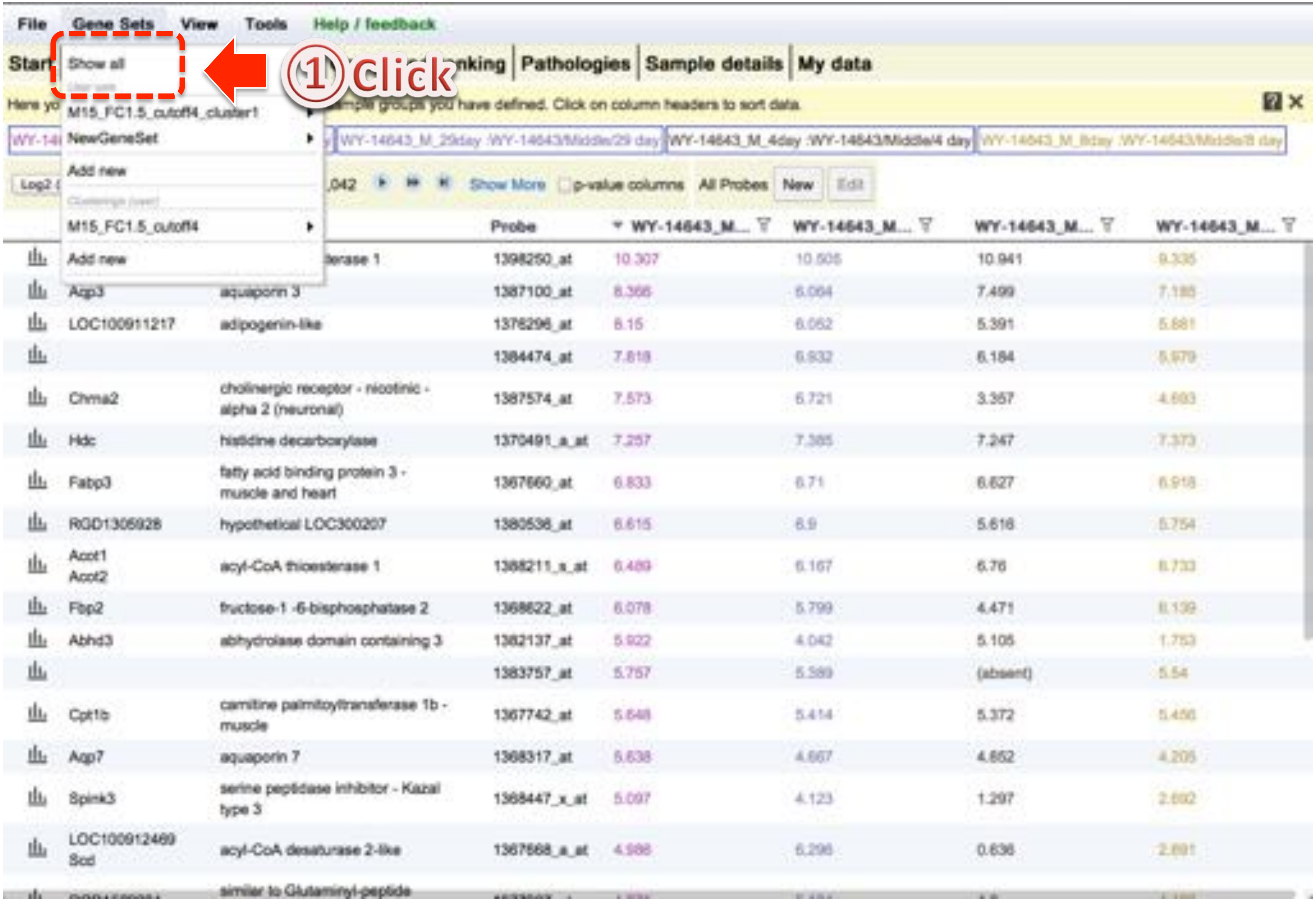
File
Gene Sets
View
Tools
Help / feedback
Start
Pathologies
Sample details
My data
Show all
①Click
M15_FC1.5_cutoff4_cluster1
NewGeneSet
Add new
M15_FC1.5_cutoff4
Add new
Show More
p-value columns
All Probes
New
Edit
Probe
WY-14643_M...
WY-14643_M_29day :WY-14643/Middle/29 day
WY-14643_M_4day :WY-14643/Middle/4 day
WY-14643_M_8day :WY-14643/Middle/8 day
Aqp3 aquaporin 3 1387100_at 8.366 6.064 7.499 7.185
LOC100911217 adipogenin-like 1376296_at 8.15 6.052 5.391 5.881
1384474_at 7.818 6.932 6.184 5.979
Chrna2 cholinergic receptor - nicotinic - alpha 2 (neuronal) 1387574_at 7.573 6.721 3.357 4.693
Hdc histidine decarboxylase 1370491_a_at 7.257 7.385 7.247 7.373
Fabp3 fatty acid binding protein 3 - muscle and heart 1367660_at 6.833 6.71 6.627 6.918
RGD1305928 hypothetical LOC300207 1380536_at 6.615 6.9 5.616 5.754
Acot1 Acot2 acyl-CoA thioesterase 1 1388211_s_at 6.489 6.167 6.76 6.733
Fbp2 fructose-1 -6-bisphosphatase 2 1368622_at 6.078 5.799 4.471 6.139
Abhd3 abhydrolase domain containing 3 1382137_at 5.922 4.042 5.105 1.753
1383757_at 5.757 5.389 (absent) 5.54
Cpt1b carnitine palmitoyltransferase 1b - muscle 1367742_at 5.648 5.414 5.372 5.456
Aqp7 aquaporin 7 1368317_at 5.638 4.667 4.652 4.205
Spink3 serine peptidase inhibitor - Kazal type 3 1368447_x_at 5.097 4.123 1.297 2.692
LOC100912469 Scd acyl-CoA desaturase 2-like 1367668_a_at 4.986 6.296 0.636 2.691

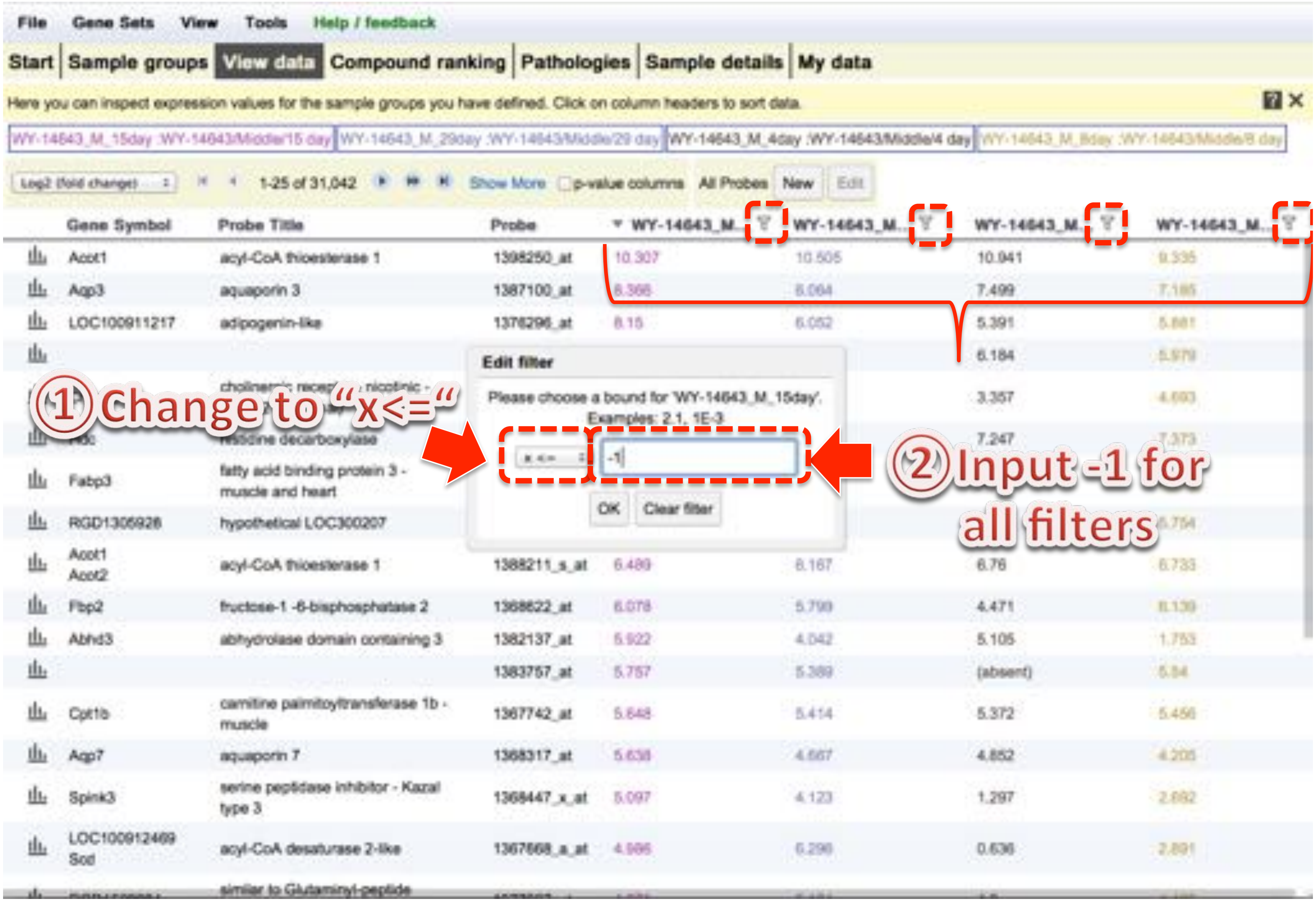
File
Gene Sets
View
Tools
Help / feedback
Start
Sample groups
View data
Compound ranking
Pathologies
Sample details
My data
Here you can inspect expression values for the sample groups you have defined. Click on column headers to sort data.
1-25 of 31,042
Show More
p-value columns
All Probes
New
Edit
Gene Symbol
Probe Title
Probe
Edit filter
OK
Clear filter
①Change to "x<="
②Input -1 for all filters

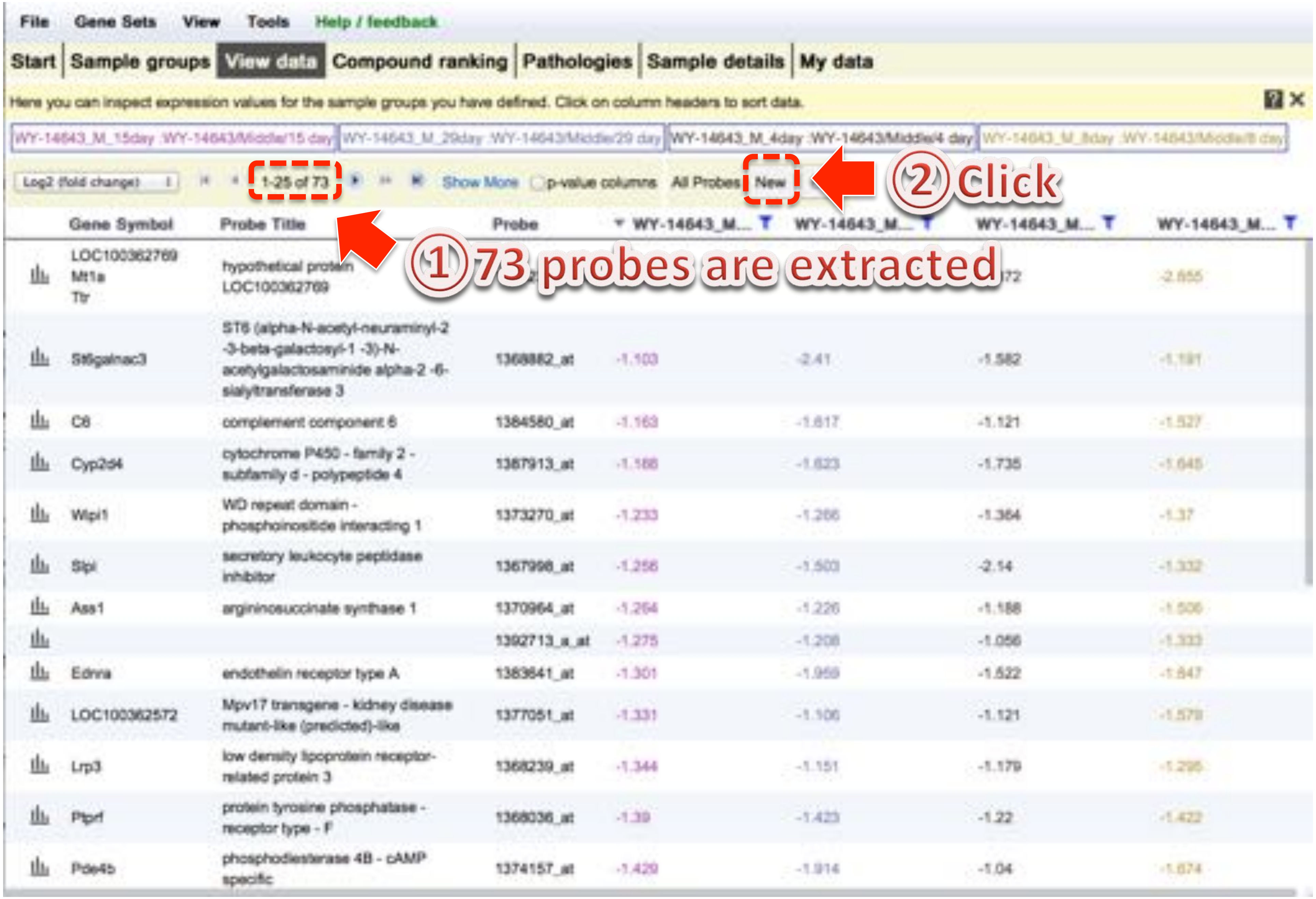

File
Gene Sets
View
Tools
Help / feedback
Start
Sample groups
View data
Compound ranking
Pathologies
Sample details
My data
Here you can inspect expression values for the sample groups you have defined. Click on column headers to sort data.
WY-14643_M_15day :WY-14643/Middle/15 day
WY-14643_M_29day :WY-14643/Middle/29 day
WY-14643_M_4day :WY-14643/Middle/4 day
WY-14643_M_8day :WY-14643/Middle/8 day
Log2 (fold change)
1-25 of 73
Show More
p-value columns
All Probes
New
②Click
①73 probes are extracted
Gene Symbol
Probe Title
Probe
WY-14643_M...
WY-14643_M...
WY-14643_M...
WY-14643_M...
LOC100362769 Mt1a Ttr hypothetical protein LOC100362769 -2.655
St6galnac3 ST6 (alpha-N-acetyl-neuraminyl-2 -3-beta-galactosyl-1 -3)-N-acetylgalactosaminide alpha-2 -6-sialyltransferase 3 1368882_at -1.103 -2.41 -1.582 -1.191
C6 complement component 6 1384580_at -1.163 -1.617 -1.121 -1.527
Cyp2d4 cytochrome P450 - family 2 - subfamily d - polypeptide 4 1387913_at -1.166 -1.623 -1.735 -1.645
Wipi1 WD repeat domain - phosphoinositide interacting 1 1373270_at -1.233 -1.266 -1.364 -1.37
Slpi secretory leukocyte peptidase inhibitor 1367998_at -1.256 -1.503 -2.14 -1.332
Ass1 argininosuccinate synthase 1 1370964_at -1.264 -1.226 -1.188 -1.506
1392713_a_at -1.275 -1.208 -1.056 -1.333
Ednra endothelin receptor type A 1383641_at -1.301 -1.959 -1.522 -1.847
LOC100362572 Mpv17 transgene - kidney disease mutant-like (predicted)-like 1377051_at -1.331 -1.106 -1.121 -1.579
Lrp3 low density lipoprotein receptor-related protein 3 1368239_at -1.344 -1.151 -1.179 -1.295
Ptprf protein tyrosine phosphatase - receptor type - F 1368036_at -1.39 -1.423 -1.22 -1.422
Pde4b phosphodiesterase 4B - cAMP specific 1374157_at -1.429 -1.914 -1.04 -1.674

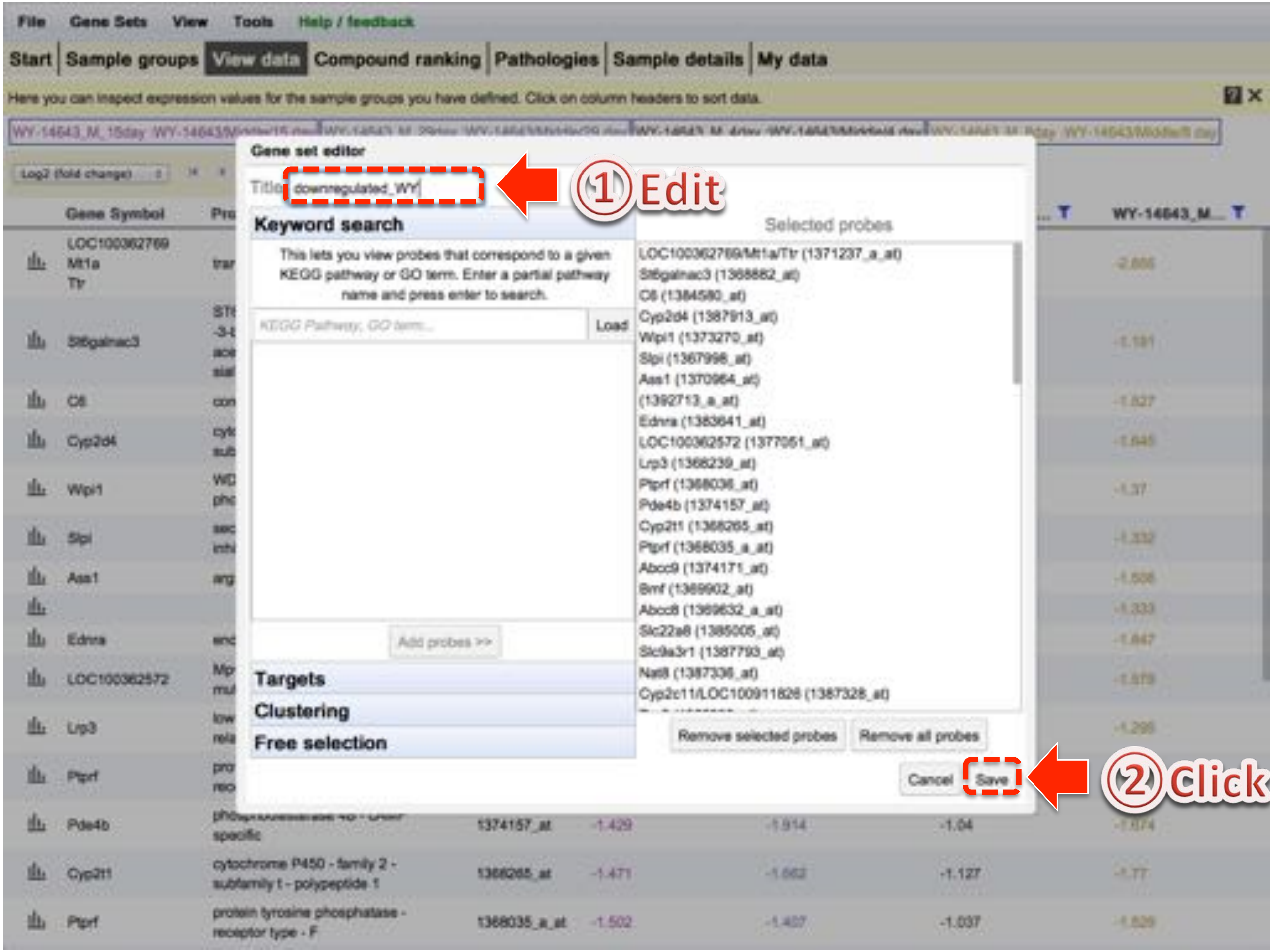
File
Gene Sets
View
Tools
Help / feedback
Start
Sample groups
View data
Compound ranking
Pathologies
Sample details
My data
Here you can inspect expression values for the sample groups you have defined. Click on column headers to sort data.
Gene set editor
Title
downregulated_WY
①Edit
Keyword search
This lets you view probes that correspond to a given KEGG pathway or GO term. Enter a partial pathway name and press enter to search.
Load
Add probes >>
Targets
Clustering
Free selection
Selected probes
LOC100362769/Mt1a/Ttr (1371237_a_at)
St6galnac3 (1368882_at)
C6 (1384580_at)
Cyp2d4 (1387913_at)
Wipi1 (1373270_at)
Slpi (1367998_at)
Aass1 (1370964_at)
(1392713_a_at)
Ednra (1383641_at)
LOC100362572 (1377051_at)
Lrp3 (1368239_at)
Ptprf (1368036_at)
Pde4b (1374157_at)
Cyp2t1 (1368265_at)
Ptprf (1368035_a_at)
Abcc9 (1374171_at)
Bmf (1369902_at)
Abcc8 (1369632_a_at)
Slc22a8 (1385005_at)
Slc9a3r1 (1387793_at)
Nat8 (1387336_at)
Cyp2c11/LOC100911826 (1387328_at)
Remove selected probes
Remove all probes
Cancel
Save
②Click
Gene Symbol
LOC100362769
Mt1a
Ttr
St6galnac3
C6
Cyp2d4
Wipi1
Slpi
Aass1
Ednra
LOC100362572
Lrp3
Ptprf
Pde4b
Cyp2t1
Ptprf
cytochrome P450 - family 2 - subfamily t - polypeptide 1
protein tyrosine phosphatase - receptor type - F
1374157_at
1368265_at
1368035_a_at

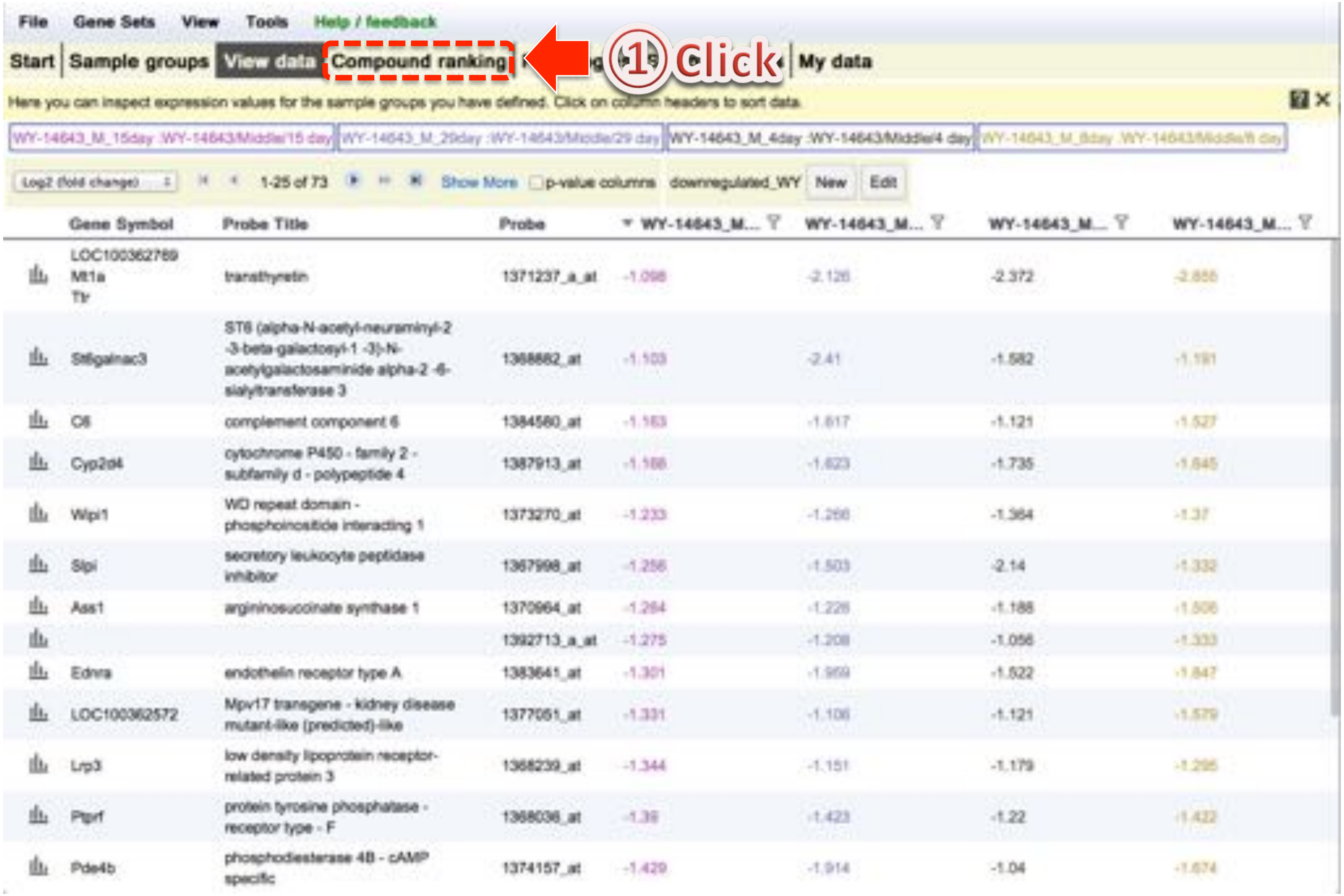

File
Gene Sets
View
Tools
Help / feedback
Start
Sample groups
View data
Compound ranking
①Click
My data
Here you can inspect expression values for the sample groups you have defined. Click on column headers to sort data.
WY-14643_M_15day :WY-14643/Middle/15 day
WY-14643_M_29day :WY-14643/Middle/29 day
WY-14643_M_4day :WY-14643/Middle/4 day
WY-14643_M_8day :WY-14643/Middle/8 day
Log2 (fold change)
1-25 of 73
Show More
p-value columns
downregulated_WY
New
Edit
Gene Symbol
Probe Title
Probe
WY-14643_M...
WY-14643_M...
WY-14643_M...
WY-14643_M...
LOC100362769 Mt1a Ttr | transthyretin | 1371237_a_at | -1.098 | -2.126 | -2.372 | -2.855
St6galnac3 | ST6 (alpha-N-acetyl-neuraminyl-2-3-beta-galactosyl-1-3)-N-acetylgalactosaminide alpha-2-6-sialyltransferase 3 | 1368882_at | -1.103 | -2.41 | -1.582 | -1.191
C6 | complement component 6 | 1384580_at | -1.163 | -1.617 | -1.121 | -1.527
Cyp2d4 | cytochrome P450 - family 2 - subfamily d - polypeptide 4 | 1387913_at | -1.188 | -1.623 | -1.735 | -1.845
Wipi1 | WD repeat domain - phosphoinositide interacting 1 | 1373270_at | -1.233 | -1.266 | -1.364 | -1.37
Slpi | secretory leukocyte peptidase inhibitor | 1367998_at | -1.256 | -1.503 | -2.14 | -1.332
Ass1 | argininosuccinate synthase 1 | 1370964_at | -1.284 | -1.226 | -1.188 | -1.506
1392713_a_at | -1.275 | -1.208 | -1.056 | -1.333
Ednra | endothelin receptor type A | 1383641_at | -1.301 | -1.969 | -1.522 | -1.847
LOC100362572 | Mpv17 transgene - kidney disease mutant-like (predicted)-like | 1377051_at | -1.331 | -1.106 | -1.121 | -1.579
Lrp3 | low density lipoprotein receptor-related protein 3 | 1368239_at | -1.344 | -1.151 | -1.179 | -1.295
Ptprf | protein tyrosine phosphatase - receptor type - F | 1368036_at | -1.39 | -1.423 | -1.22 | -1.422
Pde4b | phosphodiesterase 4B - cAMP specific | 1374157_at | -1.429 | -1.914 | -1.04 | -1.674

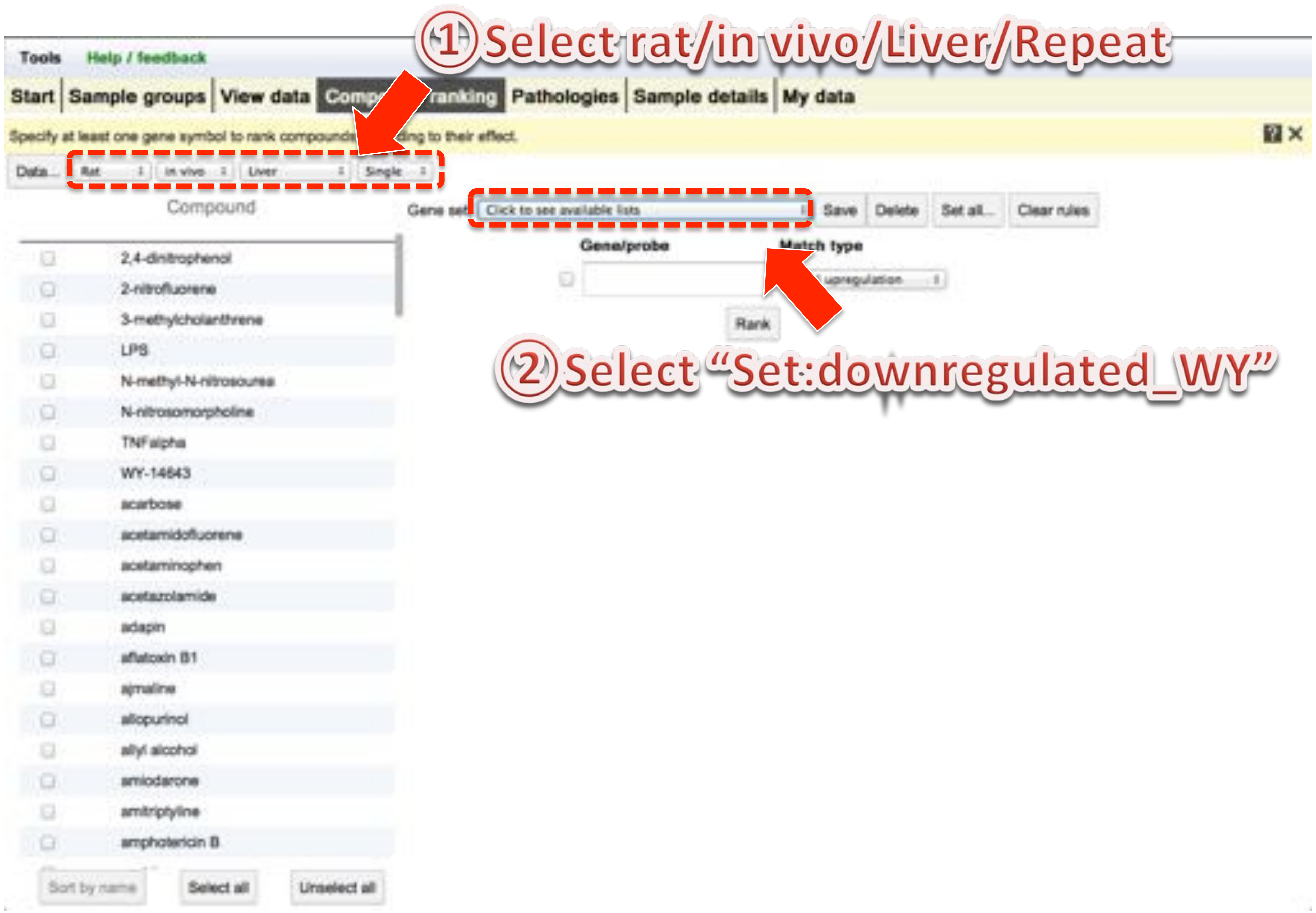

①Select rat/in vivo/Liver/Repeat
Tools
Help / feedback
Start
Sample groups
View data
ranking
Pathologies
Sample details
My data
Specify at least one gene symbol to rank compounds according to their effect.
Data...
Rat
in vivo
Liver
Single
Compound
Gene set
Click to see available lists
Save
Delete
Set all...
Clear rules
Gene/probe
Match type
upregulation
Rank
②Select "Set:downregulated_WY"
2,4-dinitrophenol
2-nitrofluorene
3-methylcholanthrene
LPS
N-methyl-N-nitrosourea
N-nitrosomorpholine
TNFalpha
WY-14643
acarbose
acetamidofluorene
acetaminophen
acetazolamide
adapin
aflatoxin B1
ajmaline
allopurinol
allyl alcohol
amiodarone
amitriptyline
amphotericin B
Sort by name
Select all
Unselect all

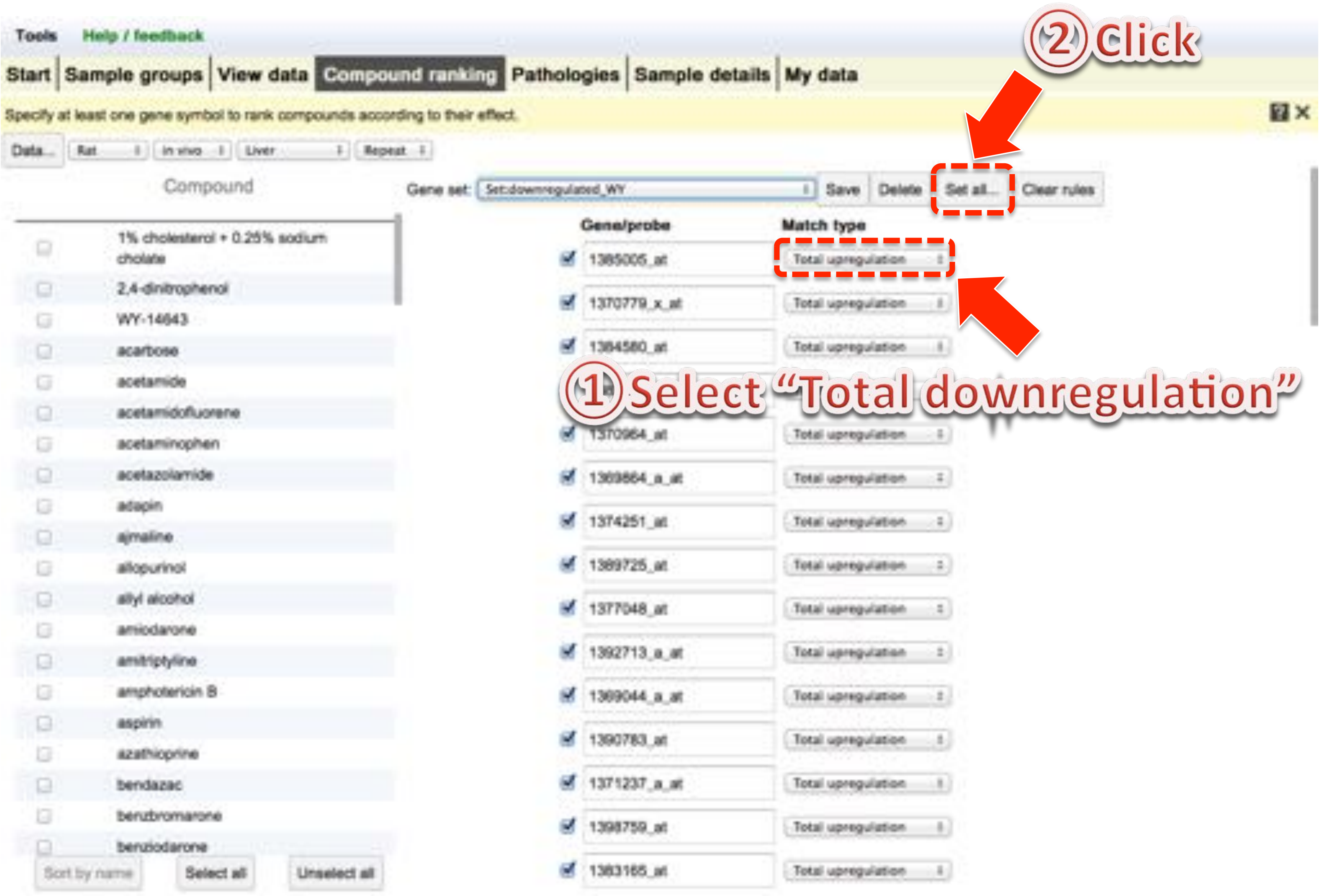
②Click
①Select "Total downregulation"
Tools
Help / feedback
Start
Sample groups
View data
Compound ranking
Pathologies
Sample details
My data
Specify at least one gene symbol to rank compounds according to their effect.
Data...
Rat
in vivo
Liver
Repeat
Compound
Gene set:
Set:downregulated_WY
Save
Delete
Set all...
Clear rules
Gene/probe
Match type
1% cholesterol + 0.25% sodium cholate
2,4-dinitrophenol
WY-14643
acarbose
acetamide
acetamidofluorene
acetaminophen
acetazolamide
adapin
ajmaline
allopurinol
allyl alcohol
amiodarone
amitriptyline
amphotericin B
aspirin
azathioprine
bendazac
benzbromarone
benziodarone
Sort by name
Select all
Unselect all
1385005_at
1370779_x_at
1384580_at
1370964_at
1369864_a_at
1374251_at
1389725_at
1377048_at
1392713_a_at
1369044_a_at
1390783_at
1371237_a_at
1398759_at
1383165_at
Total upregulation

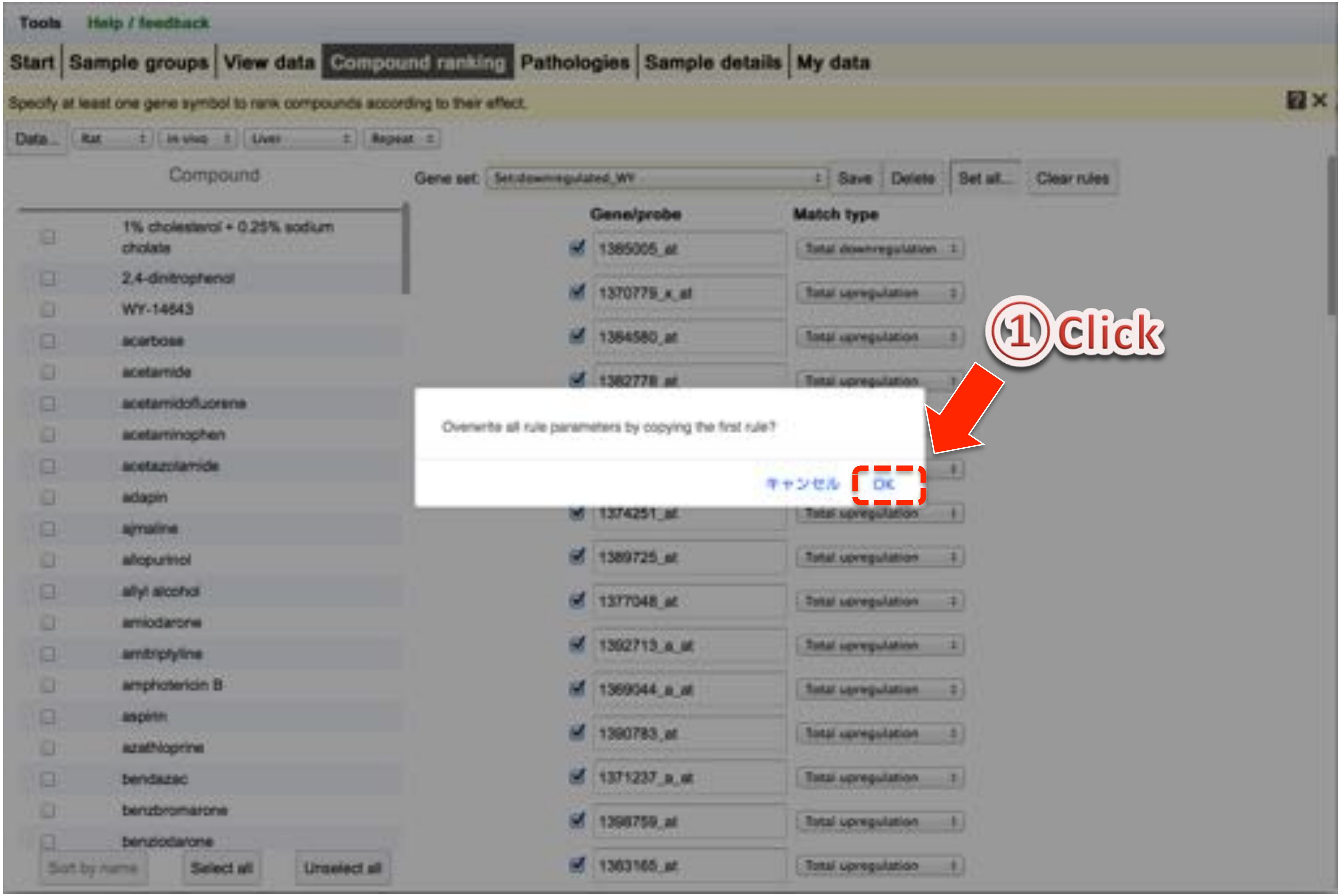
Tools
Help / feedback
Start
Sample groups
View data
Compound ranking
Pathologies
Sample details
My data
Specify at least one gene symbol to rank compounds according to their effect.
Compound
Gene set:
Save
Delete
Set all...
Clear rules
Gene/probe
Match type
Overwrite all rule parameters by copying the first rule?
キャンセル
OK
①Click
Sort by name
Select all
Unselect all

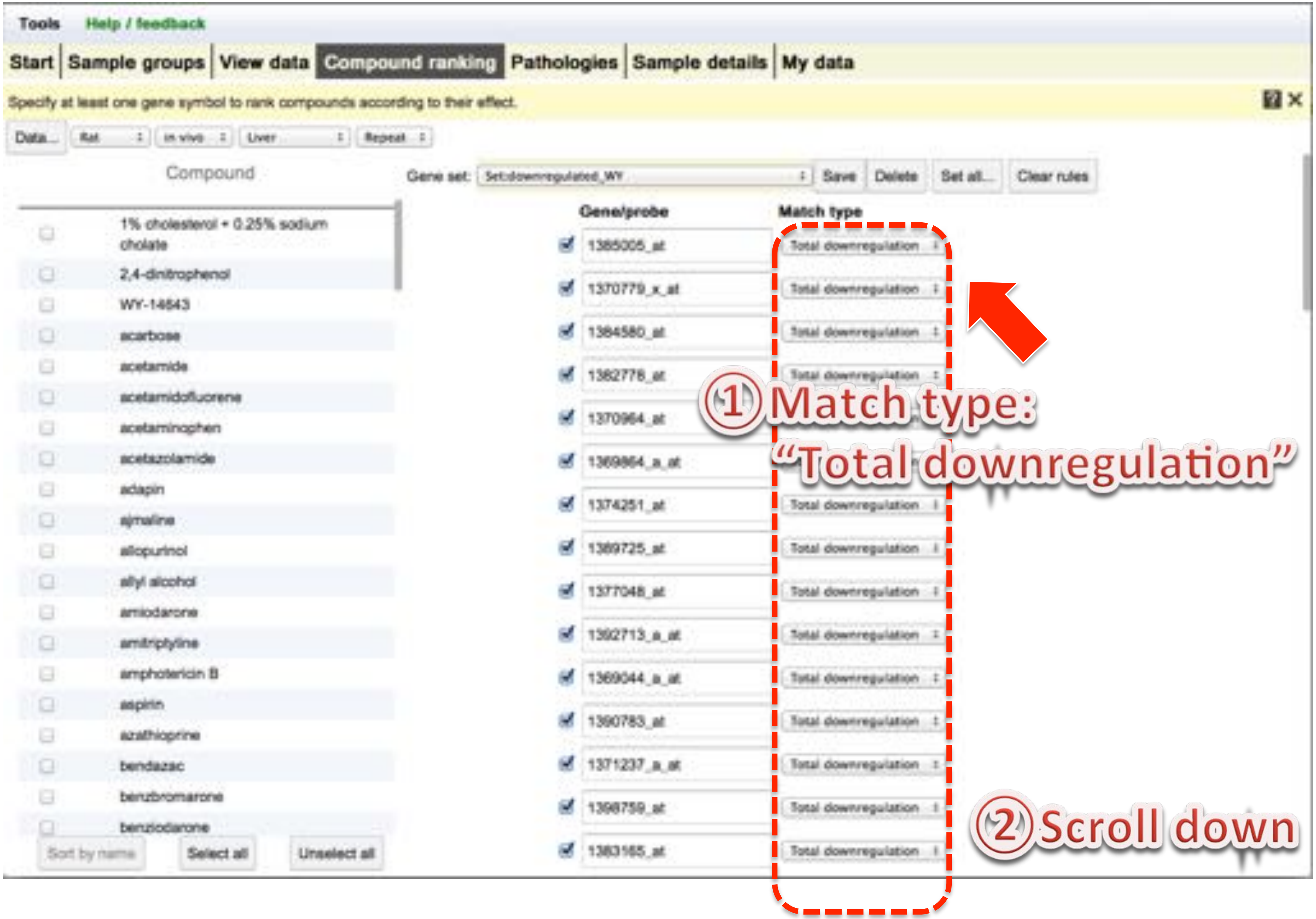

Tools
Help / feedback
Start
Sample groups
View data
Compound ranking
Pathologies
Sample details
My data
Specify at least one gene symbol to rank compounds according to their effect.
Compound
Gene set:
Save
Delete
Set all...
Clear rules
Gene/probe
Match type
Total downregulation
Sort by name
Select all
Unselect all
①Match type:
“Total downregulation”
②Scroll down

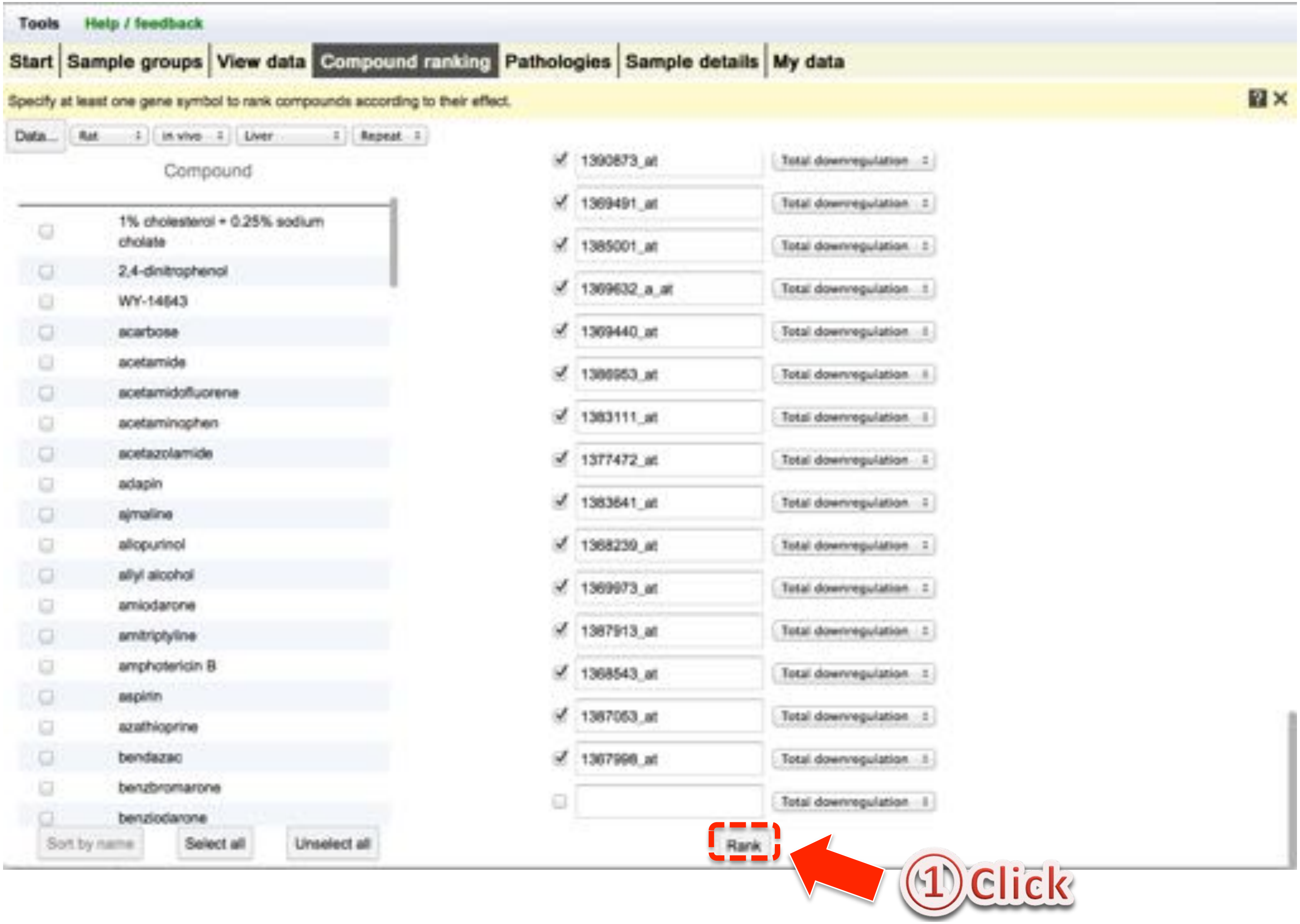
Tools
Help / feedback
Start
Sample groups
View data
Compound ranking
Pathologies
Sample details
My data
Specify at least one gene symbol to rank compounds according to their effect.
Data...
Rat
in vivo
Liver
Repeat
Compound
1% cholesterol + 0.25% sodium cholate
2,4-dinitrophenol
WY-14643
acarbose
acetamide
acetamidofluorene
acetaminophen
acetazolamide
adapin
ajmaline
allopurinol
allyl alcohol
amiodarone
amitriptyline
amphotericin B
aspirin
azathioprine
bendazac
benzbromarone
benziodarone
Sort by name
Select all
Unselect all
Total downregulation
Rank
①Click

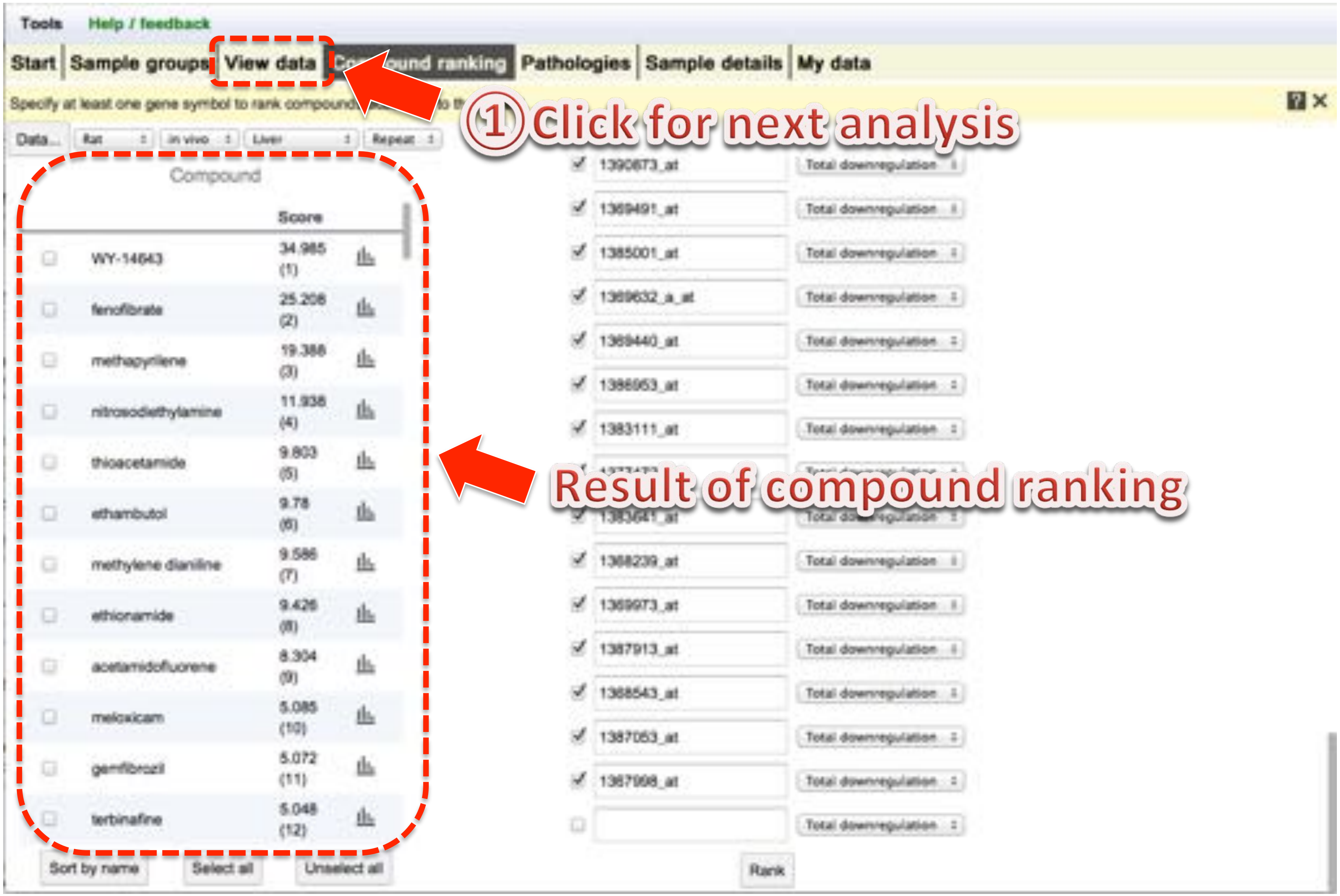
Tools
Help / feedback
Start
Sample groups
View data
Compound ranking
Pathologies
Sample details
My data
Specify at least one gene symbol to rank compound
①Click for next analysis
Data...
Rat
in vivo
Liver
Repeat
Compound
Score
WY-14643
34.985 (1)
fenofibrate
25.208 (2)
methapyrilene
19.388 (3)
nitrosodiethylamine
11.938 (4)
thioacetamide
9.803 (5)
ethambutol
9.78 (6)
methylene dianiline
9.586 (7)
ethionamide
9.426 (8)
acetamidofluorene
8.304 (9)
meloxicam
5.085 (10)
gemfibrozil
5.072 (11)
terbinafine
5.048 (12)
Sort by name
Select all
Unselect all
1390673_at
1369491_at
1385001_at
1369632_a_at
1369440_at
1386953_at
1383111_at
1383641_at
1368239_at
1369973_at
1387913_at
1388543_at
1387053_at
1387998_at
Total downregulation
Result of compound ranking
Rank

Welch's t-test filtering

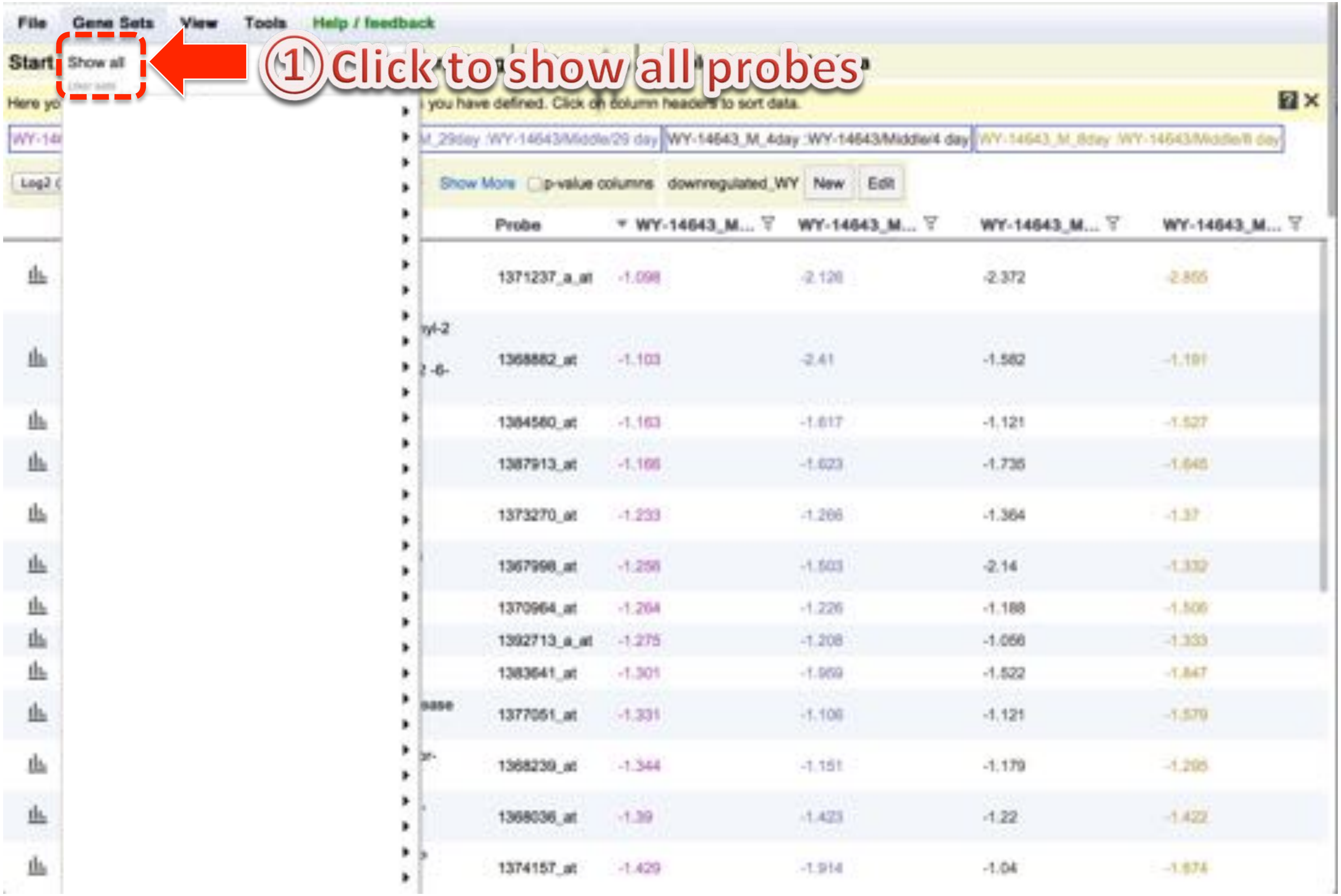

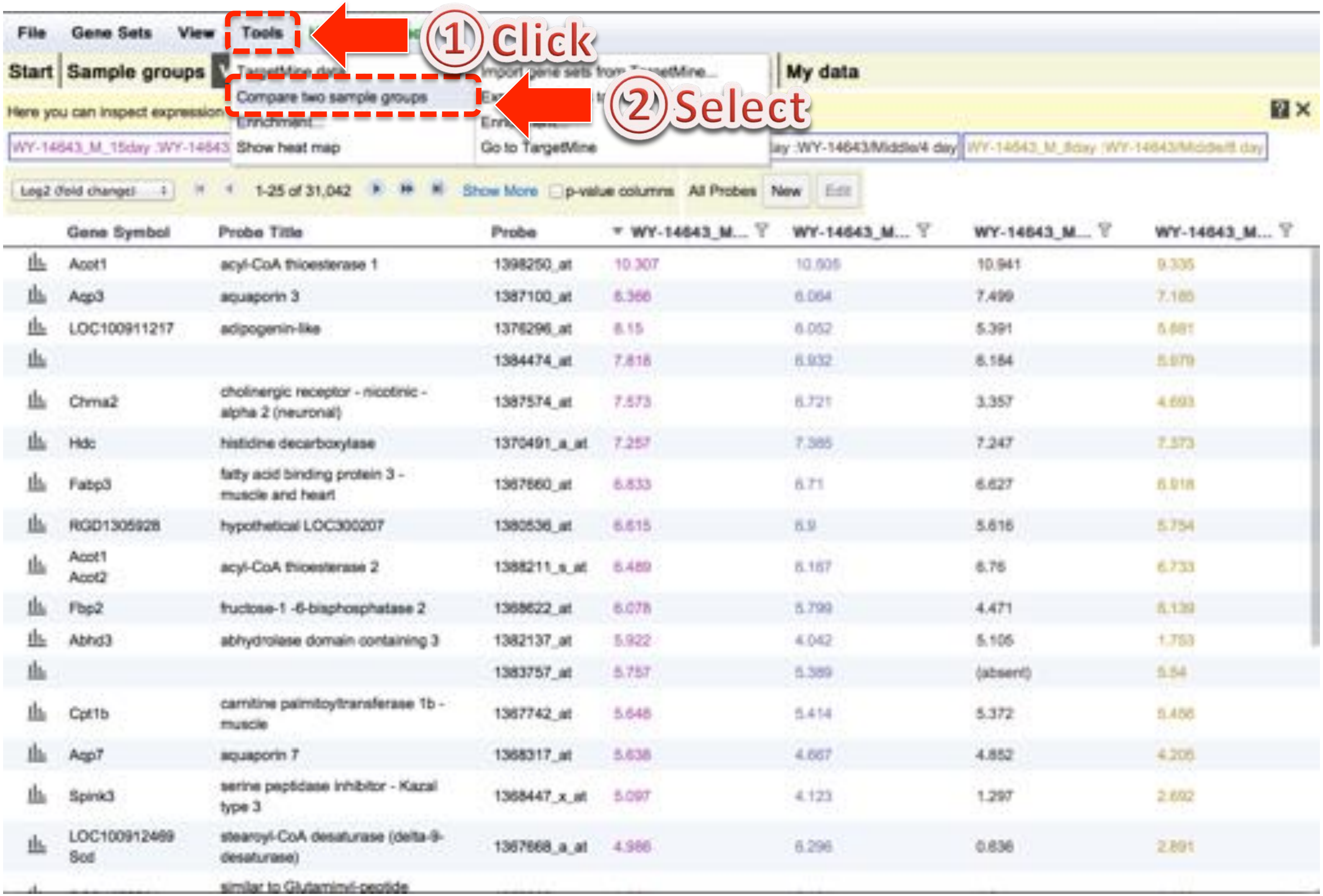
①Click
②Select
File
Gene Sets
View
Tools
Start
Sample groups
My data
Compare two sample groups
Enrichment...
Show heat map
Go to TargetMine
Here you can inspect expression
Log2 (fold change)
1-25 of 31,042
Show More
p-value columns
All Probes
New
Edit
Gene Symbol
Probe Title
Probe
WY-14643_M...
Acot1
acyl-CoA thioesterase 1
1398250_at
Aqp3
aquaporin 3
1387100_at
LOC100911217
adipogenin-like
1376296_at
1384474_at
Chrna2
cholinergic receptor - nicotinic - alpha 2 (neuronal)
1387574_at
Hdc
histidine decarboxylase
1370491_a_at
Fabp3
fatty acid binding protein 3 - muscle and heart
1367660_at
RGD1305928
hypothetical LOC300207
1380536_at
Acot1
Acot2
acyl-CoA thioesterase 2
1388211_s_at
Fbp2
fructose-1 -6-bisphosphatase 2
1368622_at
Abhd3
abhydrolase domain containing 3
1382137_at
1383757_at
(absent)
Cpt1b
carnitine palmitoyltransferase 1b - muscle
1367742_at
Aqp7
aquaporin 7
1368317_at
Spink3
serine peptidase inhibitor - Kazal type 3
1368447_x_at
LOC100912469
Scd
stearoyl-CoA desaturase (delta-9-desaturase)
1367668_a_at
similar to Glutaminyl-peptide

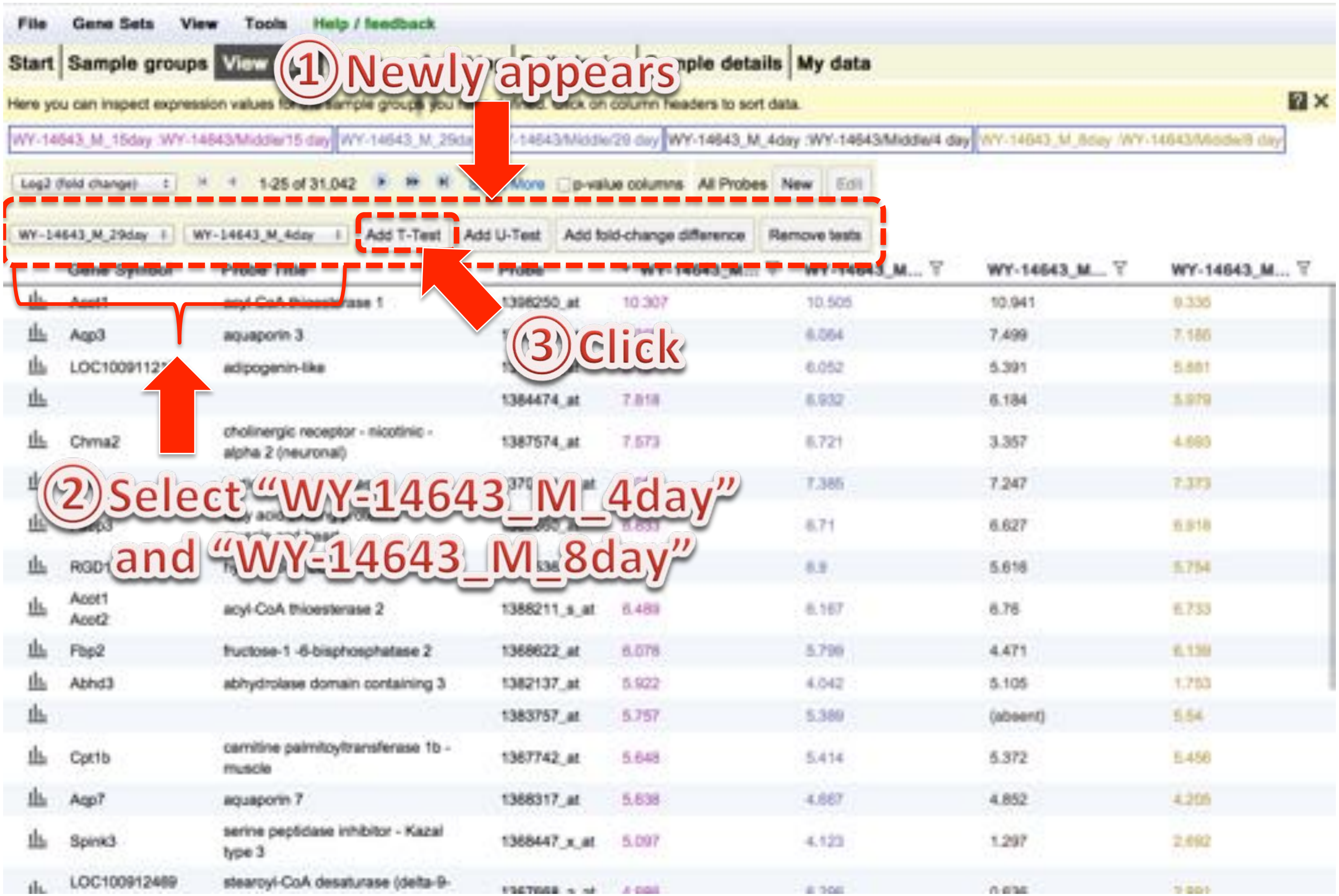
File
Gene Sets
View
Tools
Help / feedback
Start
Sample groups
Sample details
My data
①Newly appears
WY-14643_M_29day
WY-14643_M_4day
Add T-Test
Add U-Test
Add fold-change difference
Remove tests
③Click
②Select “WY-14643_M_4day”
and “WY-14643_M_8day”

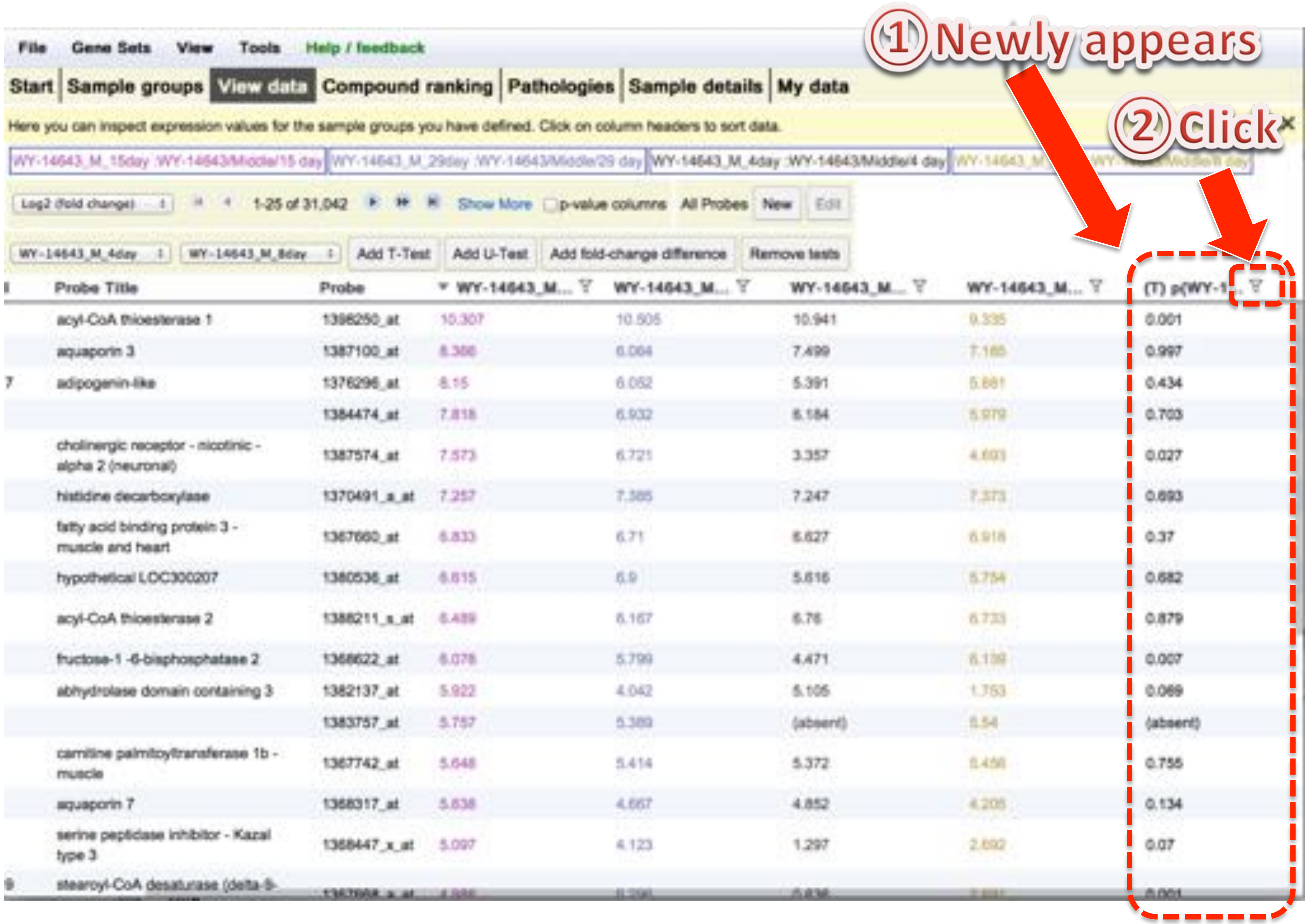
①Newly appears
②Click
File Gene Sets View Tools Help / feedback
Start Sample groups View data Compound ranking Pathologies Sample details My data
Here you can inspect expression values for the sample groups you have defined. Click on column headers to sort data.
1-25 of 31,042
Show More
p-value columns
All Probes
New
Edit
Add T-Test
Add U-Test
Add fold-change difference
Remove tests
Probe Title
Probe
(T) p(WY-1...
acyl-CoA thioesterase 1
aquaporin 3
adipogenin-like
cholinergic receptor - nicotinic - alpha 2 (neuronal)
histidine decarboxylase
fatty acid binding protein 3 - muscle and heart
hypothetical LOC300207
acyl-CoA thioesterase 2
fructose-1 -6-bisphosphatase 2
abhydrolase domain containing 3
carnitine palmitoyltransferase 1b - muscle
aquaporin 7
serine peptidase inhibitor - Kazal type 3

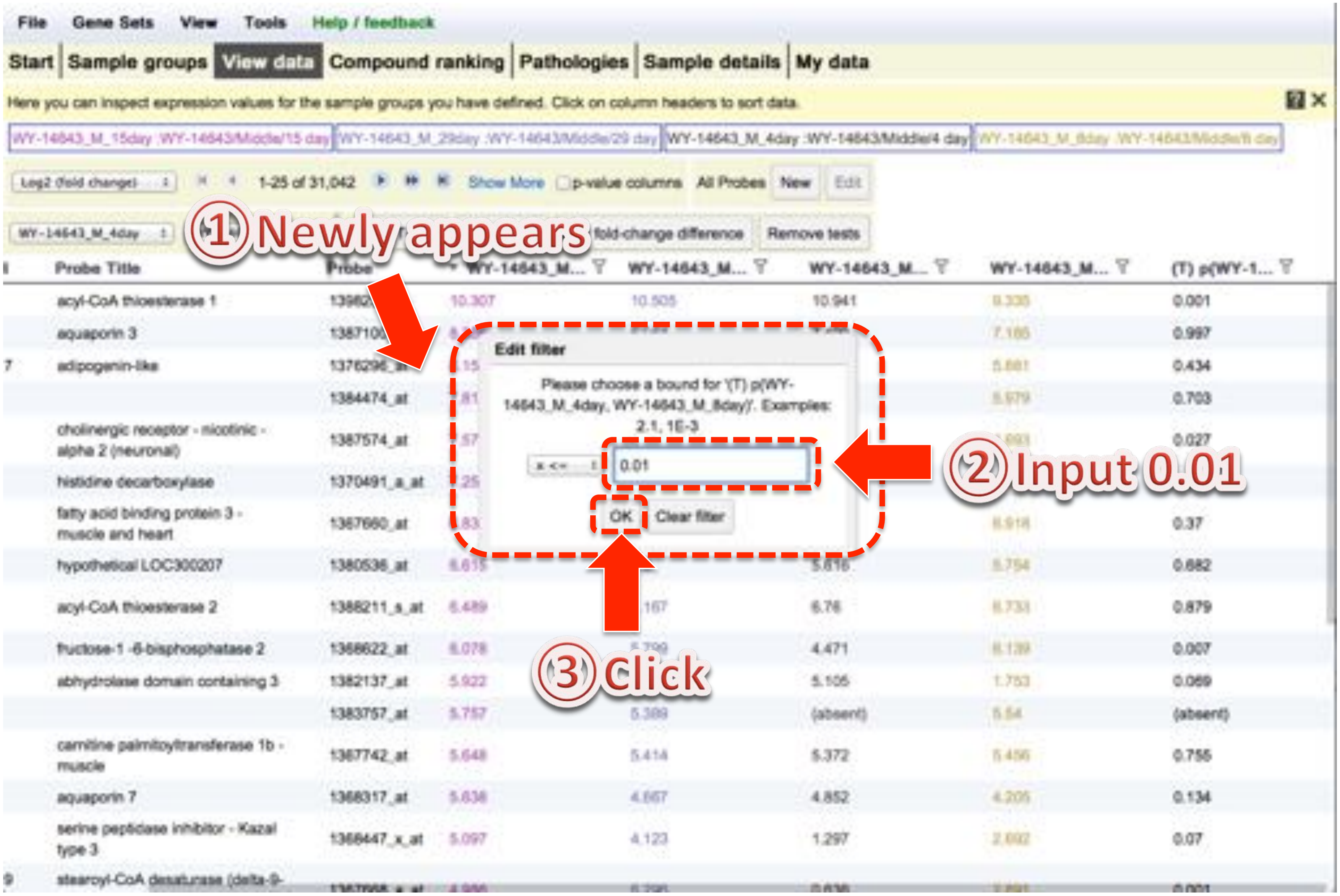
File Gene Sets View Tools Help / feedback
Start Sample groups View data Compound ranking Pathologies Sample details My data
Here you can inspect expression values for the sample groups you have defined. Click on column headers to sort data.
1-25 of 31,042
Show More
p-value columns
All Probes
New
Edit
fold-change difference
Remove tests
Probe Title
Probe
Edit filter
OK
Clear filter
①Newly appears
②Input 0.01
③Click

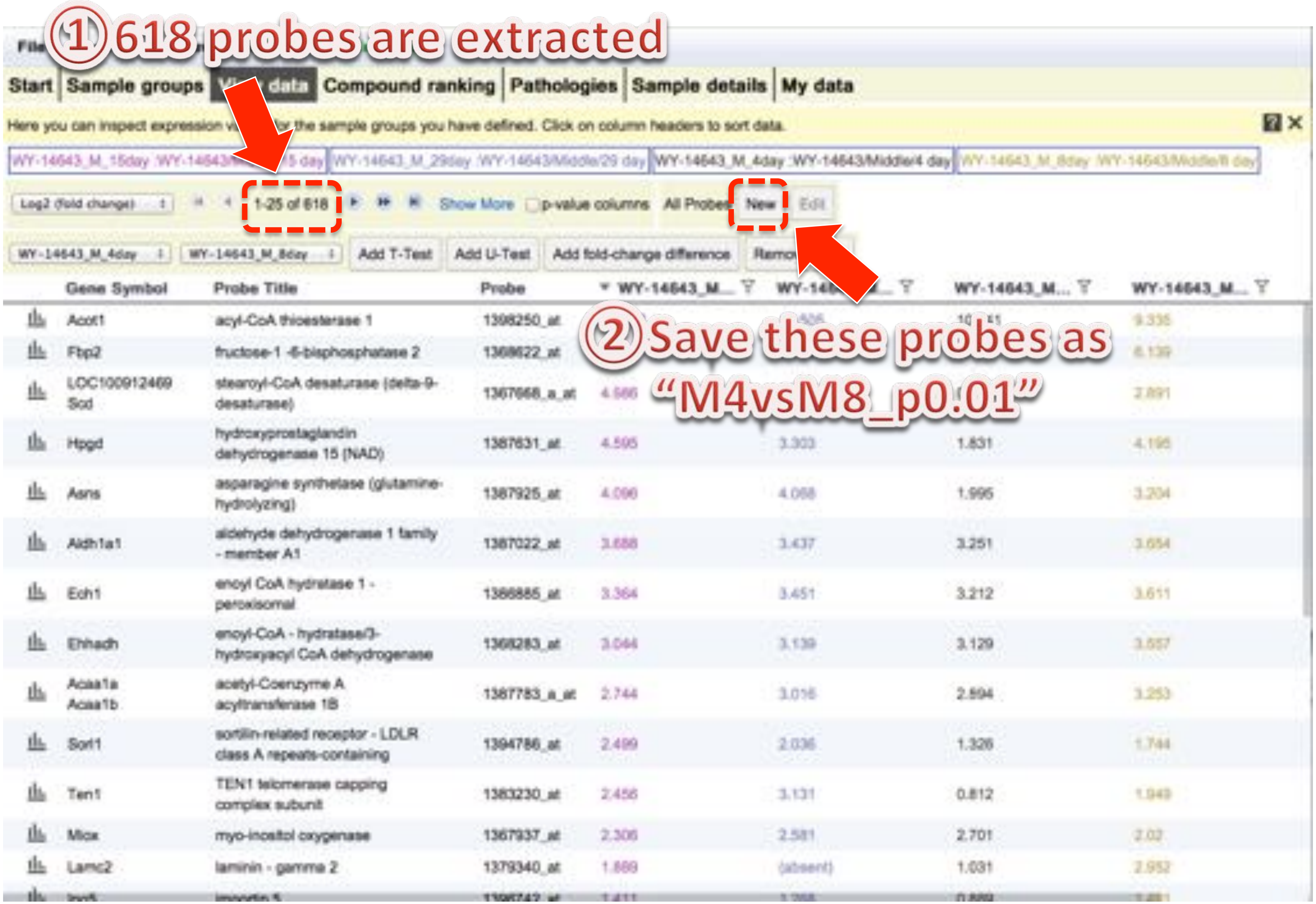
①618 probes are extracted
1-25 of 618
New
②Save these probes as
"M4vsM8_p0.01"
Start
Sample groups
Compound ranking
Pathologies
Sample details
My data
Add T-Test
Add U-Test
Add fold-change difference
Gene Symbol
Probe Title
Probe

Uploading user data to aid the investigation

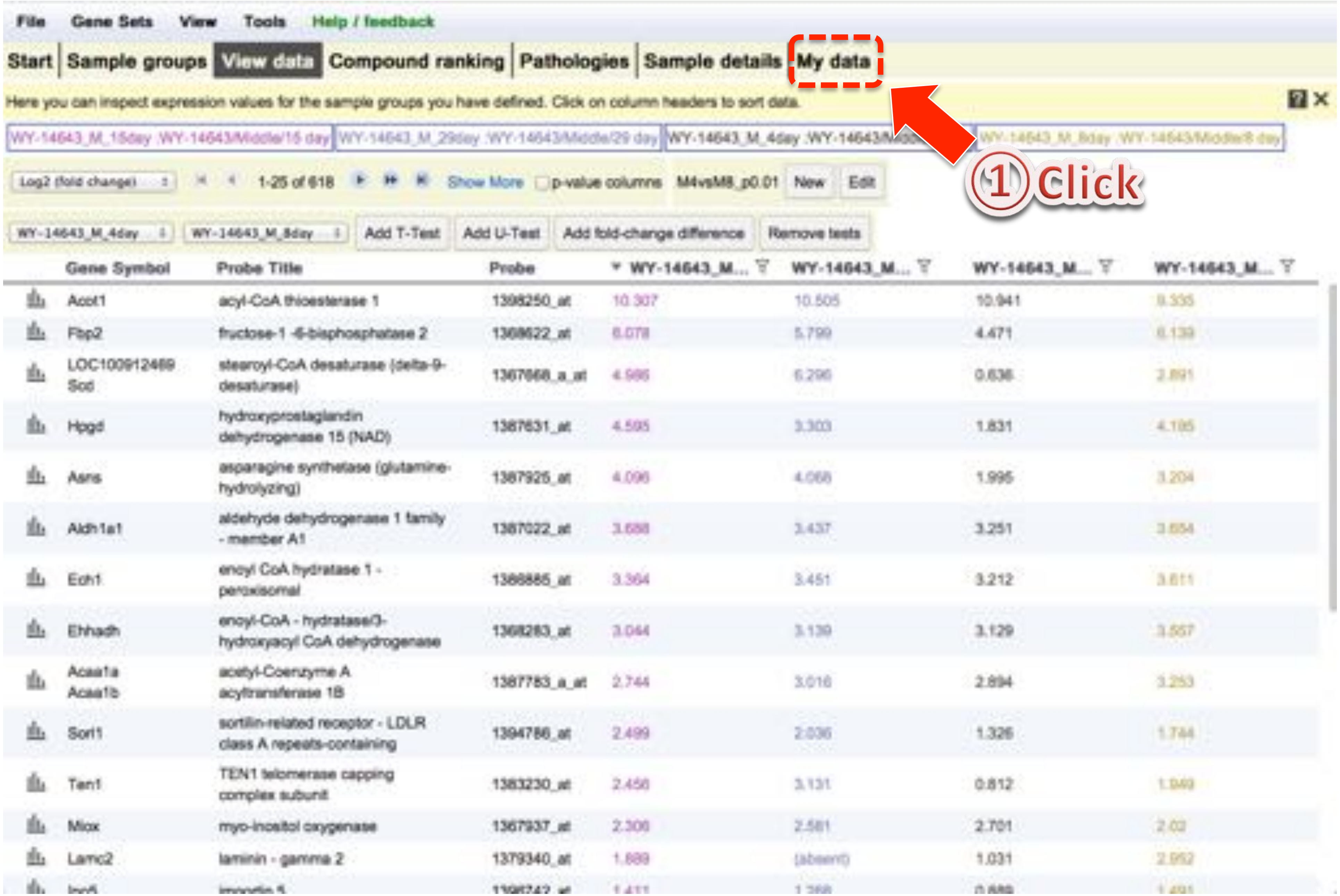

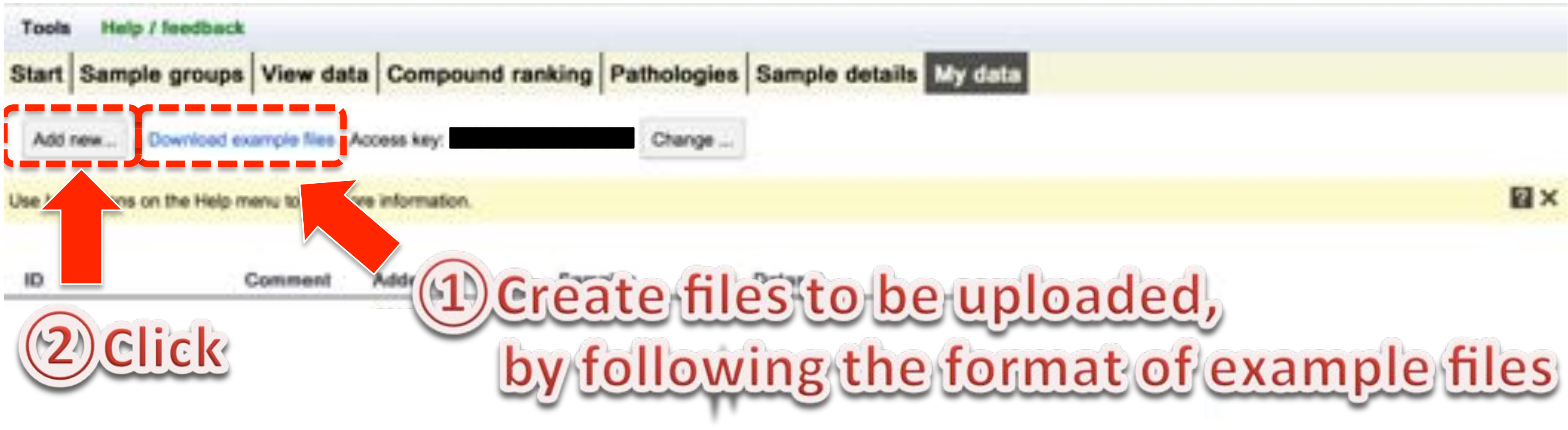
Tools
Help / feedback
Start
Sample groups
View data
Compound ranking
Pathologies
Sample details
My data
Add new...
Download example files
Access key: [redacted]
Change ...
ID
Comment
① Create files to be uploaded,
by following the format of example files
② Click

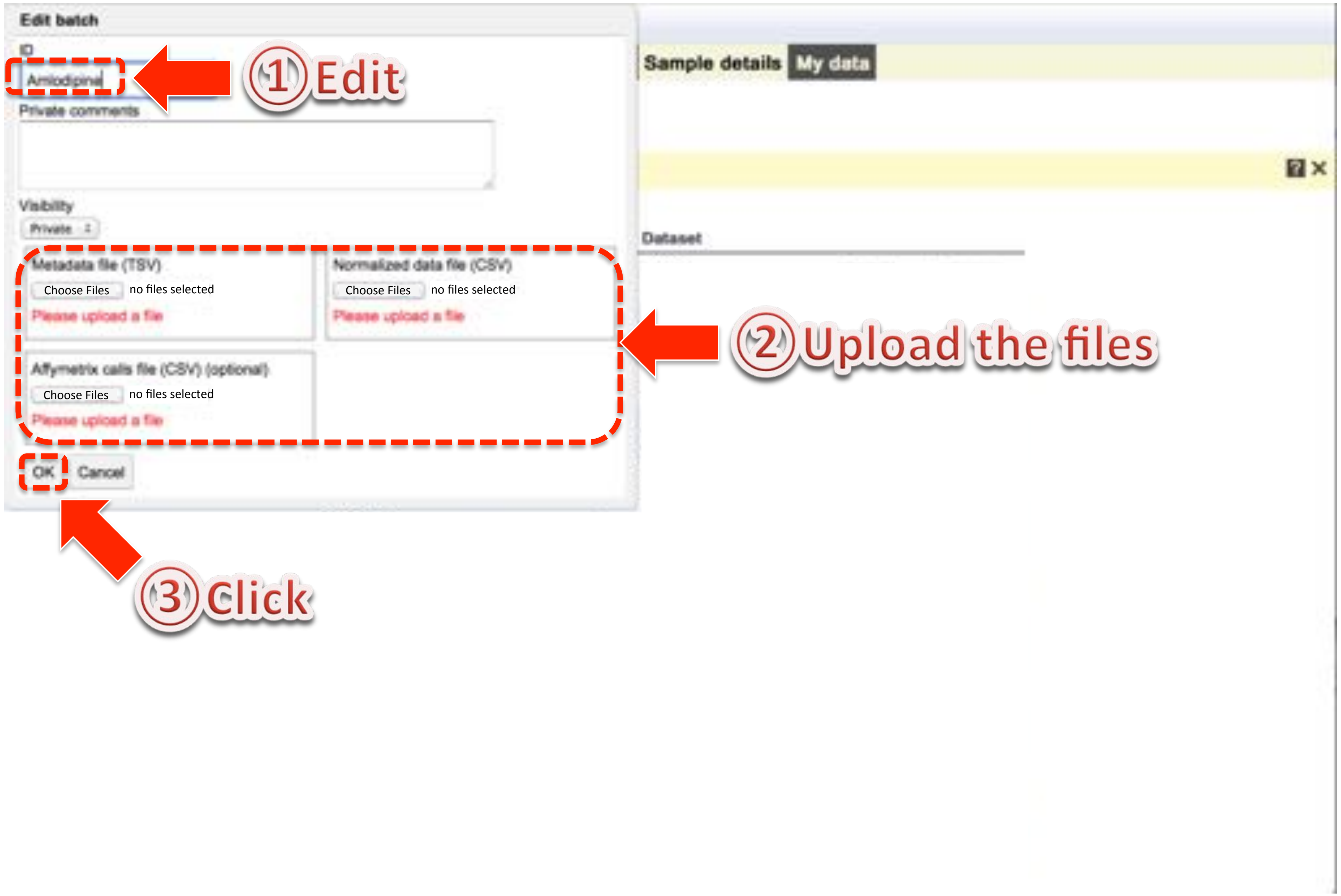
Edit batch
ID
Amlodipine
①Edit
Private comments
Visibility
Private
Metadata file (TSV)
Choose Files no files selected
Please upload a file
Normalized data file (CSV)
Choose Files no files selected
Please upload a file
Affymetrix calls file (CSV) (optional)
Choose Files no files selected
Please upload a file
OK
Cancel
②Upload the files
③Click
Sample details
My data
Dataset

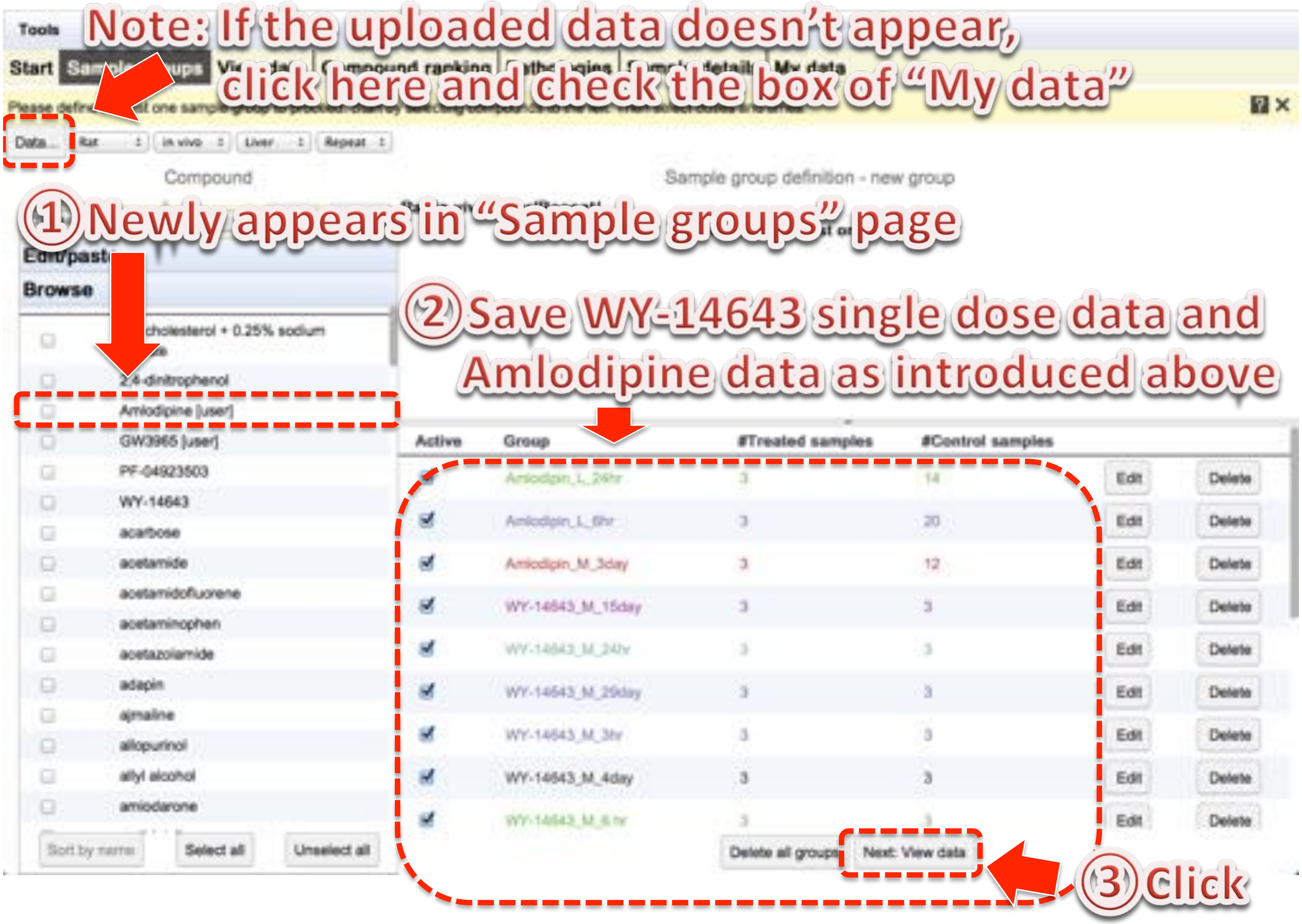
Note: If the uploaded data doesn't appear,
click here and check the box of "My data"
Tools
Start
Data...
Compound
Sample group definition - new group
①Newly appears in "Sample groups" page
Browse
2,4-dinitrophenol
Amlodipine [user]
GW3965 [user]
PF-04923503
WY-14643
acarbose
acetamide
acetamidofluorene
acetaminophen
acetazolamide
adapin
ajmaline
allopurinol
allyl alcohol
amiodarone
Sort by name
Select all
Unselect all
②Save WY-14643 single dose data and
Amlodipine data as introduced above
Active
Group
#Treated samples
#Control samples
Amlodipin_L_6hr
Amlodipin_M_3day
WY-14643_M_15day
WY-14643_M_24hr
WY-14643_M_29day
WY-14643_M_3hr
WY-14643_M_4day
Edit
Delete
Delete all groups
Next: View data
③Click

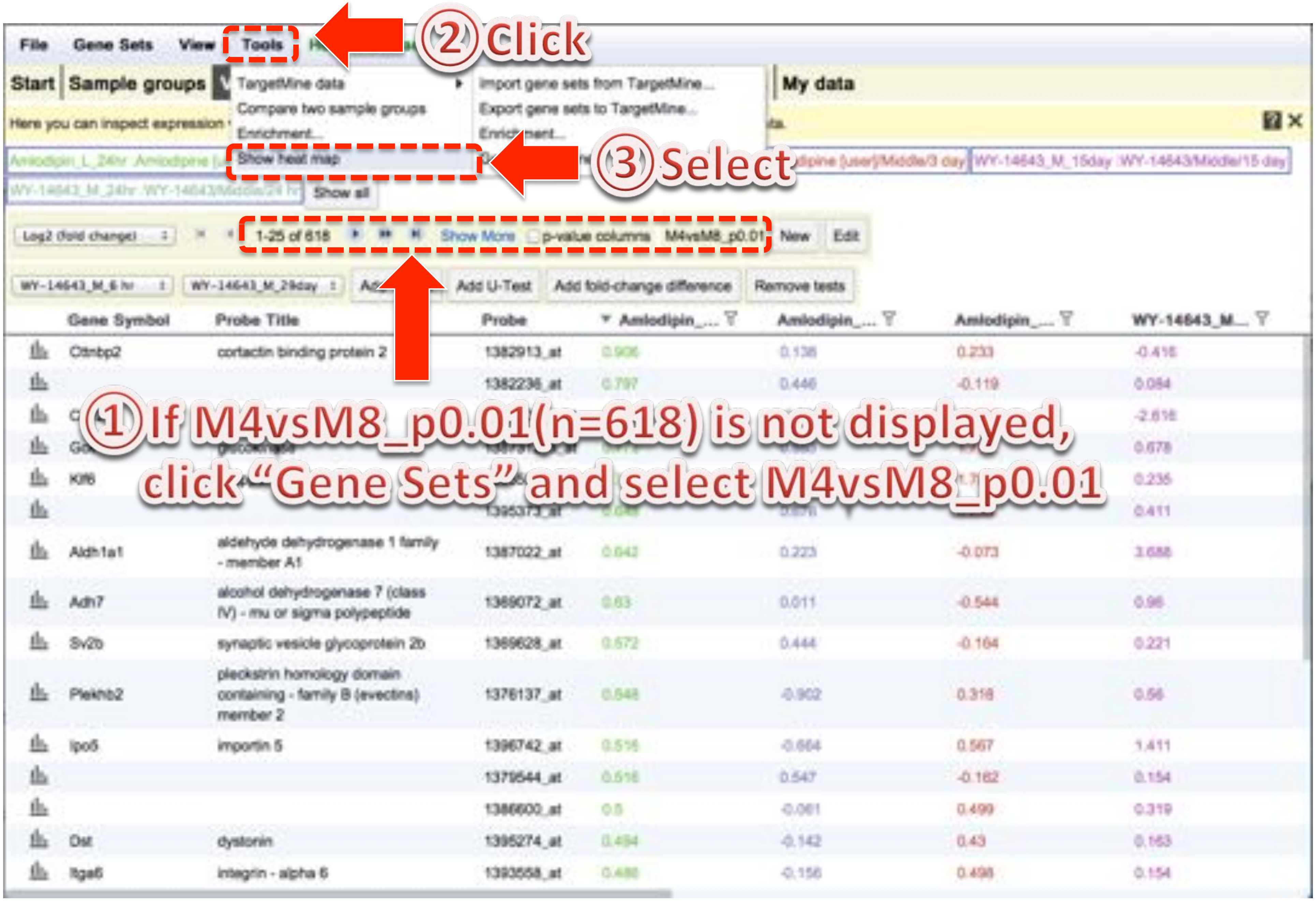
②Click
③Select
①If M4vsM8_p0.01(n=618) is not displayed,
click "Gene Sets" and select M4vsM8_p0.01
File
Gene Sets
View
Tools
Start
Sample groups
My data
TargetMine data
Compare two sample groups
Enrichment...
Show heat map
Import gene sets from TargetMine...
Export gene sets to TargetMine...
Enrichment...
Show all
1-25 of 618
Show More
p-value columns
M4vsM8_p0.01
New
Edit
Log2 (fold change)
Add U-Test
Add fold-change difference
Remove tests
Gene Symbol
Probe Title
Probe

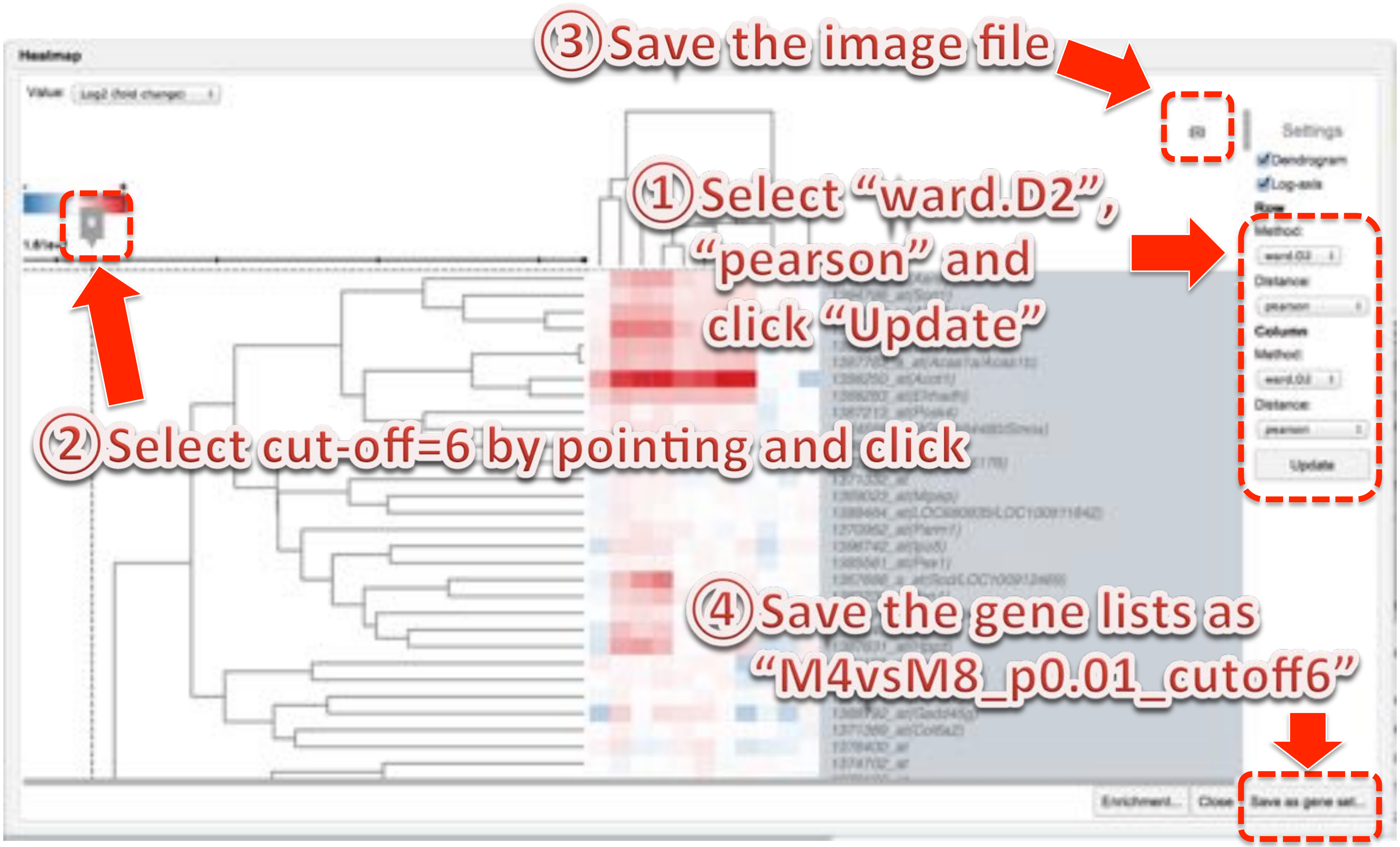
③Save the image file
①Select "ward.D2", "pearson" and click "Update"
②Select cut-off=6 by pointing and click
④Save the gene lists as "M4vsM8_p0.01_cutoff6"
Heatmap
Settings
Dendrogram
Log-axis
Row
Method:
Distance:
Column
Method:
Distance:
Update
Enrichment...
Close
Save as gene set...

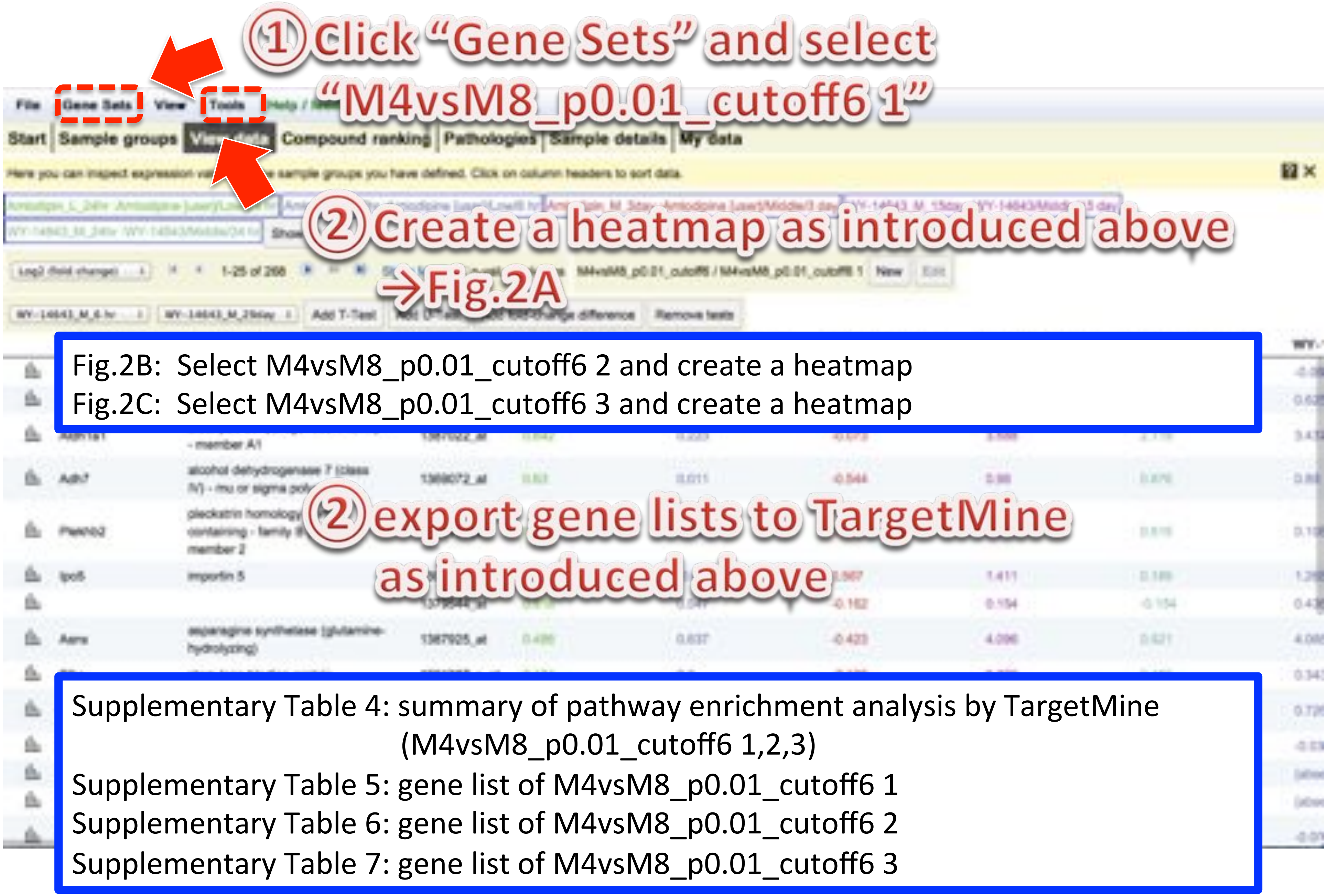

Fig.2B: Select M4vsM8_p0.01_cutoff6 2 and create a heatmap
Fig.2C: Select M4vsM8_p0.01_cutoff6 3 and create a heatmap

Supplementary Table 4: summary of pathway enrichment analysis by TargetMine (M4vsM8_p0.01_cutoff6 1,2,3)
Supplementary Table 5: gene list of M4vsM8_p0.01_cutoff6 1
Supplementary Table 6: gene list of M4vsM8_p0.01_cutoff6 2
Supplementary Table 7: gene list of M4vsM8_p0.01_cutoff6 3
